# Equilibrated Gas and Carbonate Standard-Derived Dual (Δ47 and Δ48) Clumped Isotope Values

**DOI:** 10.1029/2022gc010458

**Published:** 2022-09-27

**Authors:** Jamie K. Lucarelli, Hannah M. Carroll, Robert N. Ulrich, Ben M. Elliott, Tyler B. Coplen, Robert A. Eagle, Aradhna Tripati

**Affiliations:** 1 Department of Earth, Planetary, and Space Sciences, Department of Atmospheric and Oceanic Sciences, Center for Diverse Leadership in Science, Institute of the Environment and Sustainability, UCLA, Los Angeles, CA, USA; 2 US Geological Survey, Reston, VA, USA

## Abstract

Carbonate clumped isotope geochemistry has primarily focused on mass spectrometric determination of *m/z* 47 CO_2_ for geothermometry, but theoretical calculations and recent experiments indicate paired analysis of the *m/z* 47 (^13^C^18^O^16^O) and *m/z* 48 (^12^C^18^O^18^O) isotopologues (referred to as ${\text{Δ}}_{47}$ and ${\text{Δ}}_{48}$) can be used to study non-equilibrium isotope fractionations and refine temperature estimates. We utilize 5,448 ${\text{Δ}}_{47}$ and 3,400 ${\text{Δ}}_{48}$ replicate measurements of carbonate samples and standards, and 183 ${\text{Δ}}_{47}$ and 195 ${\text{Δ}}_{48}$ replicate measurements of gas standards from 2015 to 2021 from a multi-year and multi-instrument data set to constrain ${\text{Δ}}_{47}$ and ${\text{Δ}}_{48}$ values for 27 samples and standards, including Devils Hole cave calcite, and study equilibrium ${\text{Δ}}_{47}$–${\text{Δ}}_{48}$, ${\text{Δ}}_{47}$-temperature, and ${\text{Δ}}_{48}$-temperature relationships. We compare results to previously published findings and calculate equilibrium regressions based on data from multiple laboratories. We report acid digestion fractionation factors, $\text{Δ}{*}_{63-47}$ and $\text{Δ}{*}_{64-48}$, and account for their dependence on the initial clumped isotope values of the mineral.

## Plain Language Summary

A powerful tool for reconstructing past temperatures is based on the extent of carbonate ions in carbonate minerals with more than one heavy isotope substitution, termed carbonate clumped isotope thermometry. The use of the clumped isotope thermometer assumes that the mineral formed under isotopic equilibrium conditions, however, some carbonate minerals do not form at equilibrium. When carbonate minerals do not form at isotopic equilibrium, they are typically not useful for temperature reconstructions. It is now possible to simultaneously determine the extent of heavy isotope substitution in mass 47 and mass 48 CO_2_ isotopologues from carbonate minerals precipitated at equilibrium. This relationship is useful to identify kinetic effects in sample measurements and recover formation temperature. Here, we use experimental measurements and theory to constrain the equilibrium relationships between heavy isotope substitution in mass 47 and mass 48 CO_2_ isotopologues, and their relationships to formation temperature.

## 1. Introduction

Carbonate clumped isotope thermometry is a tool for paleotemperature reconstruction (Eiler, 2011) in the geosciences which is based on temperature dependent equilibrium constants for internal isotope exchange reactions in carbonate minerals (Ghosh et al., 2006; Schauble et al., 2006). For minerals that form in isotopic equilibrium, the frequency with which rare, heavy isotopes in carbonate minerals are bonded to each other (instead of bonded to much more common light isotopes) relative to a stochastic (random) distribution is proportional to precipitation temperature (Ghosh et al., 2006; Schauble et al., 2006).

There are multiple clumped isotopologues containing paired heavy isotopes in carbonate minerals that can potentially be used for geothermometry. The abundance of the dominant *m/z* 63 isotopologue (^13^C^18^O^16^O_2_) forms the basis of the most widely used thermometer. The acid digestion of minerals containing carbonate ion groups with *m/z* 63 yields *m/z* 47 CO_2_, which can be measured by isotope ratio mass spectrometry (Ghosh et al., 2006). Theory predicted that the lower abundance *m/z* 48 CO_2_ isotopologue derived from acid digestion of *m/z* 64 (^12^C^18^O_2_^16^O) carbonate ion groups could be used for geothermometry (Ghosh et al., 2006; Guo et al., 2009, 2019; Hill et al., 2014; Tripati et al., 2015) and this has recently been confirmed through experimentation (Bajnai et al., 2020; Fiebig et al., 2019, 2021; Swart et al., 2021).

The abundance of the ^13^C^18^O^16^O and ^12^C^18^O^18^O isotopologues is denoted with $\delta_{47}$, $\delta_{48}$, ${\text{Δ}}_{47}$, and ${\text{Δ}}_{48}$ notation (Eiler & Schauble, 2004). These are defined as:

(1) $$\delta_{47}=\left(\text{R}47_{\text{sample}}/\text{R}47_{\text{ref}.\;\text{gas}}-1\right)\times 1000$$

(2) $$\delta_{48}=\left(\text{R}48_{\text{sample}}/\text{R}48_{\text{ref}.\;\text{gas}}-1\right)\times 1000$$

(3) $${\text{Δ}}_{47}=\left(\text{R}47_{\text{sample}}/\text{R}47_{\text{stochastic}}-1\right)\times 1000$$

(4) $${\text{Δ}}_{48}=\left(\text{R}48_{\text{sample}}/\text{R}48_{\text{stochastic}}-1\right)\times 1000$$

where $Ri_{\text{sample}}$ is the measured ratio of *i*/44 CO_2_ isotopologues in the sample, $Ri_{\text{stochastic}}$ is the ratio of *i*/44 CO_2_ isotopologues that would be expected in a random distribution, and $Ri_{\text{ref}.\;\text{gas}}$ is the ratio of *i*/44 CO_2_ in a reference gas of known isotopic composition (Eiler, 2007; Schauble et al., 2006). The values are given in permil (‰). The most abundant *m/z* 48 CO_2_ isotopologue (^12^C^18^O^18^O) has two ^18^O substitutions and is therefore in extremely low abundance at 4.1 ppm in air, which is an order of magnitude lower than *m/z* 47 isotopologues at 45 ppm (Ghosh et al., 2006). The minor *m/z* 48 CO_2_ isotopologue (^13^C^18^O^17^O) has an abundance of 16.7 ppb (Ghosh et al., 2006).

The precise measurement of ${\text{Δ}}_{47}$ was enabled by modification of the Thermo MAT 253, specially configured for the digestion of carbonate minerals, purification of liberated CO_2_, and *m/z* 47–49 Faraday cups (Eiler & Schauble, 2004; Ghosh et al., 2006). On this instrument, *m/z* 48 isotopologues were used only to screen for contaminants. More precise measurements of ${\text{Δ}}_{48}$ have recently emerged due to the use of 10^13^ Ω resistors for *m/z* 47–49 Faraday cups in the Thermo MAT 253 Plus (Bajnai et al., 2020; Fiebig et al., 2019, 2021; Swart et al., 2021), and secondary electron suppression in the Nu Perspective IS. These advances contribute to increased accuracy and precision for determination of ${\text{Δ}}_{48}$ values, and paired ${\text{Δ}}_{47}$ and ${\text{Δ}}_{48}$ values.

A unique attribute of carbonate clumped isotope thermometry based on ${\text{Δ}}_{47}$ or ${\text{Δ}}_{48}$ is that it does not depend on the bulk oxygen isotope composition ($\delta^{18}\text{O}$) of the water the mineral precipitated from (Ghosh et al., 2006), unlike the more widely used oxygen isotope thermometer (Urey, 1947). Measurements of ${\text{Δ}}_{47}$ have been used for the reconstruction of numerous paleo-environmental parameters, including but not limited to land (Passey & Henkes, 2012) and ocean (Henkes et al., 2018; Tripati et al., 2015) paleotemperatures, paleoelevation (Huntington et al., 2010; Lechler et al., 2013), and dinosaur body temperature (Eagle et al., 2010), while simultaneously estimating water $\delta^{18}\text{O}$. Previous research has shown that kinetic isotope effects observed in abiotic and biogenic carbonate minerals, including speleothems (Affek et al., 2008; Daëron et al., 2011) and coral (Bajnai et al., 2020; Kimball et al., 2016; Saenger et al., 2012; Thiagarajan et al., 2011), may affect the accuracy of ${\text{Δ}}_{47}$-based temperature reconstructions. However, the paired analysis of ${\text{Δ}}_{47}$ and ${\text{Δ}}_{48}$ has been shown by theory (Guo, 2020; Hill et al., 2014, 2020; Schauble et al., 2006; Tripati et al., 2015) and experimentation (Bajnai et al., 2020; Fiebig et al., 2019, 2021; Swart et al., 2021) to have a characteristic equilibrium relationship to temperature which may be used to identify and study kinetic effects in carbonate minerals.

Several studies have proposed the use of new methods to advance the consistency of ${\text{Δ}}_{47}$ measurements between laboratories. Interlaboratory reproducibility of ${\text{Δ}}_{47}$ values was advanced by using accurately determined carbonate standard values that are anchored to the absolute reference frame, using a reference frame constructed using primary gas standards, secondary carbonate standards, or a mixture of gas and carbonate standards, detailed by Dennis et al. (2011). Recent work from Bernasconi et al. (2021) has proposed nominal carbonate standard ${\text{Δ}}_{47}$ values and the use of carbonate standards for data normalization in the 90°C reference frame. These advances form the foundation for the potential application of carbonate-based data normalization to yield reproducible ${\text{Δ}}_{48}$ values, and paired ${\text{Δ}}_{47}$ and ${\text{Δ}}_{48}$ values, on the absolute reference frame.

Here, we utilize data collected over multiple years on multiple instruments to determine if carbonate-based data normalization produces reproducible ${\text{Δ}}_{48}$ values, and examine if widely used carbonate standards, in-house standards, and a suite of both biogenic and abiogenic samples of varying minerology deviate significantly from equilibrium. We used both equilibrated gas and carbonate-based data normalization to report the isotopic composition of 27 samples of varying mineralogy, including standards and 4 Devils Hole calcite samples. We determine acid digestion fractionation factors, $\text{Δ}{*}_{63-47}$ and $\text{Δ}{*}_{64-48}$, that account for the dependence on the mineral ${\text{Δ}}_{63}$ and ${\text{Δ}}_{64}$ values, respectively.

## 2. Materials and Methods

### 2.1. Samples

In total, 27 different samples were analyzed for clumped and bulk isotope compositions on mass spectrometers in the Tripati Lab at University of California, Los Angeles. Table 1 contains a description of the mineralogy and origin of all samples. These materials were chosen for analysis because many of them are standards used widely among clumped isotope laboratories, such as ETH-1, ETH-2, ETH-3, ETH-4, Carrara Marble, IAEA-C1, IAEA-C2, and Mallinckrodt. Others are used commonly in a certain region or country, such as ISTB-1, TB-1, and TB-2, which are clumped isotope standards from the China University of Geosciences. Additionally, this suite of samples includes biogenic materials (47407 Coral), and carbonates of different mineralogies (calcite, aragonite, dolomitic limestone, calcitic marble, travertine). Many also have >50 replicate analyses on one or multiple instruments that can be used to provide robust values for ${\text{Δ}}_{47}$ and ${\text{Δ}}_{48}$ measurements.

### 2.2. Devils Hole Calcite

We analyzed four Devils Hole (Amargosa Desert, Nevada) mammillary calcite samples from core DH-2 for paired Δ_47_ and Δ_48_ values, including DH-2–10 (172 ± 4 ka), DH-2–11 (163 ± 5 ka), DH-2–12 (57 ± 5 ka), and DH-2–13 (151 ± 4 ka) (Winograd et al., 1992), that previously were measured on a Thermo MAT 253 for ${\text{Δ}}_{47}$ in Tripati et al. (2015). The samples were re-analyzed on Nu Perspective mass spectrometers. Devils Hole calcite is assumed to have precipitated near isotopic equilibrium due to an extremely slow precipitation rate (0.1–0.8 μm year^−1^) in water with a low calcite saturation index (0.16–0.21) (Coplen, 2007; Kluge et al., 2014). Devils Hole is thought to have had a stable temperature of 33.7 (±0.8) °C throughout the Holocene (Coplen, 2007; Kluge et al., 2014; Winograd et al., 1988, 1992).

### 2.3. Instrumentation

Standards and samples were analyzed on three mass spectrometers using five configurations (Table 2), including Nu Perspective-EG, Nu Perspective-1, Nu Perspective-1a, Nu-Perspective-2, and MAT 253. Nu Perspective-EG is the only configuration that analyzed equilibrated gases. On both the MAT 253 and Nu Perspective mass spectrometers, the detectors for *m/z* 44, 45, and 46 are registered through 3 × 10^8^, 3 × 10^10^, and 10^11^ Ω resistors, respectively, while detectors for *m/z* 47 through 49 are registered through 10^12^ Ω resistors.

The most notable difference between the Nu Instruments Perspective and the more widely used older generation Thermo Fisher MAT 253 is the implementation in the former of electrostatic analyzers (ESAs) before the *m/*z 47–49 detectors. These ESAs consist of two curved plates with a voltage difference placed directly in front of each of the Faraday collectors. The addition of the ESAs as well as ion lenses following the magnetic sector of the flight tube removes secondary ion and electron signals from the mass detection. This removal results in a drastic reduction in the interfering signals on all masses (*m/z* 44–49) during operation, producing flatter and more stable baselines, relative to the older MAT 253 (Figure S2 in Supporting Information S1). In addition, the lowered interference, which is largely comprised of signals from secondary electrons, in the Nu Perspectives results in greater intensities and lowered noise in the signals from the higher masses, especially *m/*z 48 and 49. This advancement has contributed to a ${\text{Δ}}_{47}$ non-linearity slope for the Nu Perspective (median slope observed was −0.00005) that ranges from one to two orders of magnitude less than the MAT 253 (median slope observed was −0.007), and a ${\text{Δ}}_{48}$ non-linearity slope for the Nu Perspective (median slope observed was −0.004) that is an order of magnitude less than the MAT 253 (median slope observed was −0.013).

The Thermo Fisher MAT 253 used an autosampler similar to what is described in Passey et al. (2010) with a 105 weight % phosphoric acid bath held at 90°C. After calcite samples of 5 mg were digested, CO_2_ (g) was cryogenically purified through traps containing dry ice-cooled ethanol and liquid nitrogen, which remove low vapor pressure gases such as H_2_O (g). The CO_2_ passed through elemental silver wool (Sigma Aldrich) to remove sulfur compounds, followed by a −20°C gas chromatograph (GC) that contains Porapak Type-Q^™^ 50/80 mesh column pack material with He carrier gas. The *m/z* 44 beam intensity is 16 V. Data are acquired in 9 blocks of 10 cycles, with each consisting of 8 s of integration and 16 s of changeover delay, for a total integration time of 720 s.

Nu Perspective-EG, Nu Perspective-1, and Nu Perspective-1a used the same mass spectrometer and a similar autosampler setup as the MAT 253. Samples weighing 5 mg were analyzed in bellows on the Nu Perspective-EG and Nu Perspective-1 in 4 blocks of 20 cycles, including 8 s of changeover delay and 20 s of integration per cycle, with a total integration time of 1,600 s. The *m/z* 44 ion beam intensity was 24 V before 6/2017 and 18 V thereafter. Nu Perspective-1a analyzed 0.5 mg samples, with sample and working gas volumes depleted in microvolume mode at precisely matched rates, with *m/z* 44 ranging from 24–9 V during sample acquisition. Microvolume mode allows for a full hour-long measurement per sample. Data were taken in 3 blocks of 20 cycles, including 8 s of changeover delay and 20 s of integration per cycle, for a total integration time of 1,200 s. The sample preparation system was operated by software in Labview that controls the sampler, GC column, cryogenic dewar lifters, and valves. The Labview software is integrated with the Perspective Stable Gas Control software interface that controls the Nu Perspective mass spectrometer.

Nu Perspective-2 used a Nu Carb Sample Digestion System instead of a common acid bath, where 0.5 mg of carbonate mineral was digested at 70°C in individual glass vials with 105 wt% phosphoric acid. The sample gas was cryogenically purified in liquid nitrogen-cooled tubes called coldfingers before passing through a relatively short GC column packed with Porapak Type-Q^™^ 50/80 and silver wool. This instrument operates under vacuum pressure and does not use a carrier gas. The sample and working gas volumes were matched precisely during depletion into the mass spectrometer, with *m/z* 44 ranging from 24–9 V. Sample data were analyzed in 3 blocks of 20 cycles, with each cycle integrating for 20 s, for a total integration time of 1,200 s.

### 2.4. Equilibrated Gas Standards

We analyzed equilibrated gas standards on Nu Perspective-EG (Table 2). We utilized two gases with differing bulk isotope values, with a ∼60 ‰ difference in $\delta_{47}$ values, prepared using standard procedures (Dennis et al., 2011; Ghosh et al., 2006). The heavy isotope depleted $\delta_{47}$ gas was from an Airgas CO_2_ gas cylinder and was equilibrated with 5–10 mL of 25°C deionized (DI) water. The heavy isotope enriched $\delta_{47}$ gas was produced by phosphoric acid digestion of Carrara Marble. The resulting CO_2_ was equilibrated with evaporated DI water held at 25°C. Aliquots of the two 25°C gases were re-equilibrated at 1000°C by heating the gases in quartz tubes inside a muffle furnace for >1 hr, and then flash cooling the tubes, to produce gases with near stochastic clumped isotope values.

### 2.5. Data Processing and Normalization

Raw data files from all instrument configurations were transferred into Easotope (John & Bowen, 2016) (64-bit version from release 20201231), where corrections and final ${\text{Δ}}_{47}$ and ${\text{Δ}}_{48}$ values for replicate analyses were calculated. All data used the IUPAC parameter set (Brand et al., 2010; Daëron et al., 2016). The ${\text{Δ}}_{47}$ and ${\text{Δ}}_{48}$ data from Nu Perspective-EG is reported on the Carbon Dioxide Equilibrium Scale (CDES 90) (Dennis et al., 2011), meaning it was normalized to CO_2_ equilibrated at 25 and 1000°C with samples digested at 90°C. The ${\text{Δ}}_{47}$ data from Nu Perspective-1, Nu Perspective-1a, Nu Perspective-2, and MAT 253 is reported on the InterCarb-Carbon Dioxide Equilibrium Scale (I-CDES) (Bernasconi et al., 2021), meaning it was normalized to carbonate standards including ETH-1, ETH-2, and ETH-3 at an acid digestion temperature of 90°C. Note that the I-CDES and CDES 90 reference frames should be equivalent if standard values are accurately determined. The ${\text{Δ}}_{48}$ data for Nu Perspective-1, Nu Perspective-1a, Nu Perspective-2, and MAT 253 are reported using CDES 90, normalized to carbonate standard values determined on Nu Perspective-EG in this study, including ETH-1, ETH-2, and ETH-3, at an acid digestion temperature of 90°C. Since it is currently convention to describe ${\text{Δ}}_{48}$ values digested at 90°C as CDES 90 whether they are normalized to equilibrated CO_2_ or carbonate standard values originally determined using equilibrated CO_2_, we want to note again that the only instrument here that used equilibrated CO_2_ normalization was Nu Perspective-EG, while the others use carbonate standard-based transfer functions for normalization (Table 2).

**Figure 1.**
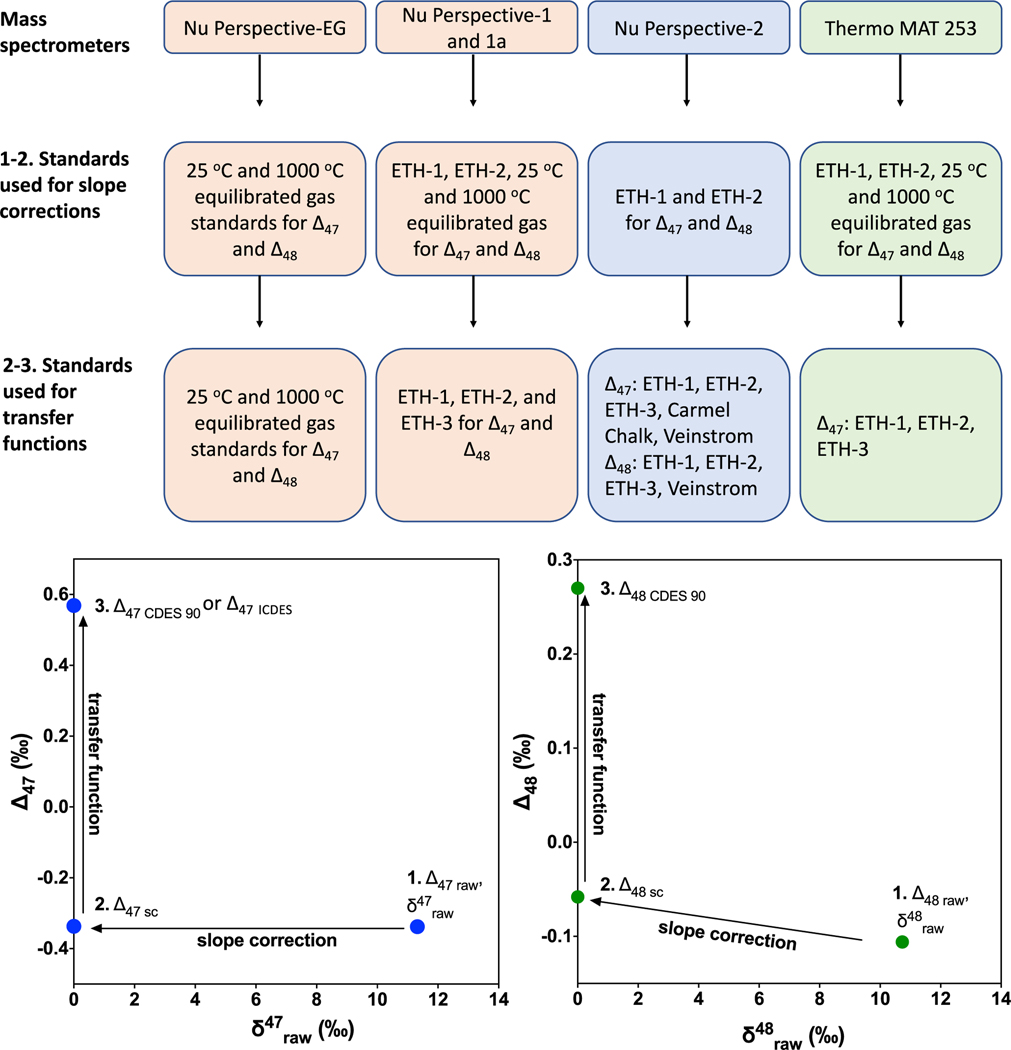
Flow chart indicating which standards are used for data normalization in each instrumental configuration, and how data are transformed at each step (following Dennis et al., 2011, Bernasconi et al., 2018, 2021).

Figure 1 contains a flow chart detailing the standards used in data normalization for each instrument configuration. Methods detailed in Dennis et al. (2011) were used to normalize data to the CDES 90 and I-CDES reference frames, including a nonlinearity correction and transfer function (Figure 1, Figure S3 in Supporting Information S1). We do not perform pressure baseline corrections; however, a background correction is performed for all masses (*m/z* 44–49) on all instruments before any further data normalization. The background is measured (in amps on the Nu Perspective instruments; mV on the MAT 253) at the start of an analysis and is subtracted from the measurement. For the nonlinearity slope correction, a slope was determined over a ±10-replicate moving average for the regression lines between $\delta_{47\;\text{raw}}$, and ${\text{Δ}}_{47\;\text{raw}}$, and $\delta_{48\;\text{raw}}$ and ${\text{Δ}}_{48\;\text{raw}}$ values for CO_2_ gas standards equilibrated at 25 and 1000°C and/or ETH-1 and ETH-2 (Figure S3 in Supporting Information S1). Nonlinearity slope corrections were applied to all analyses using Equations 5 and 6:

(5) $${\text{Δ}}_{47\;\text{sc}}={\text{Δ}}_{47\;\text{raw}}-m_{47}\times \delta_{47\;\text{raw}}$$

(6) $${\text{Δ}}_{48\;\text{sc}}={\text{Δ}}_{48\;\text{raw}}-m_{48}\times \delta_{48\;\text{raw}}$$

where ${\text{Δ}}_{47\;\text{sc}}$ and ${\text{Δ}}_{48\;\text{sc}}$ values are the nonlinearity slope-corrected values, and $m_{47}$ and $m_{48}$ are the regression slopes, with nomenclature adapted from Fiebig et al. (2019). For the ${\text{Δ}}_{47}$ transfer functions, the ±10-replicate moving average slope and intercept was determined for the linear relationship between either theoretically calculated ${\text{Δ}}_{47}$ values for 25 and 1000°C, 0.925 ‰ (Wang et al., 2004) and 0.027 ‰ (Dennis et al., 2011), respectively, or carbonate standard values, and ${\text{Δ}}_{47\;\text{sc}}$ values (Figure S3 in Supporting Information S1). Where carbonate standards were used, ${\text{Δ}}_{47}$ values determined in Bernasconi et al. (2021) of 0.2052 ‰, 0.2085 ‰, and 0.6132 ‰ were used as standard values for ETH-1, ETH-2, and ETH-3, respectively. For Nu Perspective-2, the additional in-house standards Carmel Chalk and Veinstrom were used, with ${\text{Δ}}_{47}$ values of 0.674 ‰ and 0.715 ‰, respectively. Before Carmel Chalk and Veinstrom were used in data normalization, their long-term average values were determined on Nu Perspective-1 and MAT 253. For ${\text{Δ}}_{48}$ data normalization, the ±10-replicate moving average slope and intercept was determined for the linear relationship between either theoretically calculated ${\text{Δ}}_{48}$ values for 25 and 1000°C of 0.345 ‰ (Wang et al., 2004) and 0.000 ‰ (Fiebig et al., 2019), respectively, or carbonate standards, and ${\text{Δ}}_{48\;\text{sc}}$. Where carbonate standards were used, the ${\text{Δ}}_{48}$ values determined on Nu Perspective-EG for ETH-1, ETH-2, ETH-3, and Veinstrom (Table 3) were used for standard values. The slope and intercept from these regressions were used to create transfer functions, which are applied to all ${\text{Δ}}_{47\;\text{sc}}$ and ${\text{Δ}}_{48\;\text{sc}}$ values, and yields the fully corrected ${\text{Δ}}_{47}$ and ${\text{Δ}}_{48}$ values using Equations 7 and 8:

(7) $${\text{Δ}}_{47\;\text{I}-\text{CDES};\text{CDES}\;90}={\text{Δ}}_{47\;\text{sc}}\times \text{TF}\;\text{slope}+\text{TF}\;\text{intercept}$$

(8) $${\text{Δ}}_{48\;\text{CDES}\;90}={\text{Δ}}_{48\;\text{sc}}\times \text{TF}\;\text{slope}+\text{TF}\;\text{intercept}$$

where ${\text{Δ}}_{47\;\text{I-CDES};\;\text{CDES}\;90}$ and ${\text{Δ}}_{48\;\text{CDES}\;90}$ values are the fully corrected values in the I-CDES or CDES 90 reference frame, ${\text{Δ}}_{47\;\text{sc}}$ and ${\text{Δ}}_{48\;\text{sc}}$ values are the slope corrected values from Equations 5 and 6, TF slope is the transfer function slope, and TF intercept is the transfer function intercept.

### 2.6. Use of Statistical Methods for Determination of ${\text{Δ}}_{47}$ and ${\text{Δ}}_{48}$ Values

**Figure 2.**
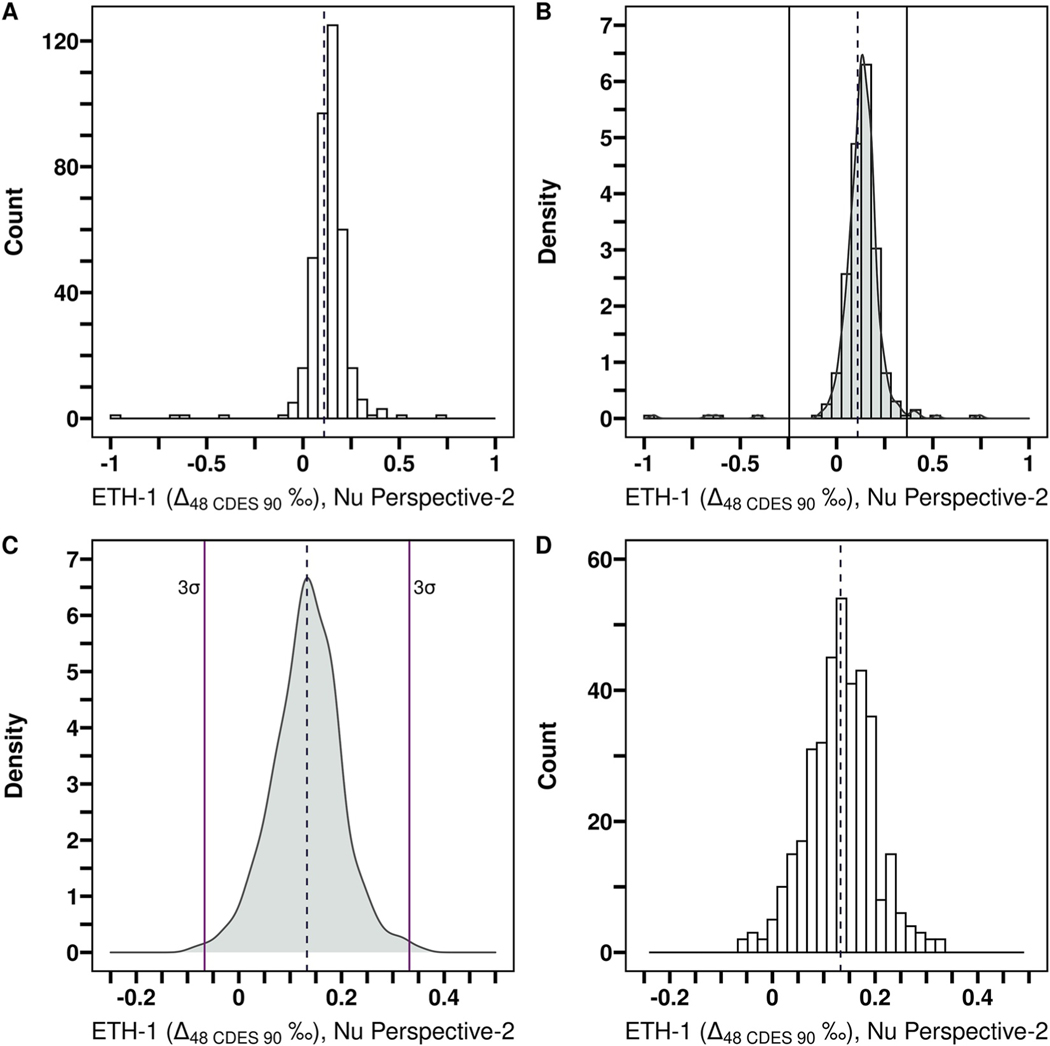
The ${\text{Δ}}_{48}$ replicate pool for carbonate standard ETH-1 is used as an example to demonstrate how the final replicate pool was determined for each standard and sample. In all panels, the dashed vertical line represents the mean value of sample replicates. (a) Histogram of the initial replicate pool (N = 389, where N is the number of sample replicates). (b) Density plot (gray shaded region) overlaid with a histogram (black rectangles) of the initial replicate pool and first outlier removal for extreme outliers (black vertical lines). (c) Density plot of the replicate pool following initial exclusions (N = 378) with replicates exceeding 3σ shown (solid purple vertical lines). (d) Histogram of the final replicate pool following a 3σ exclusion (mean = 0.133 ‰, SD = 0.065, N = 376). Note that the *x* and *y* axis scales differ between plots.

To streamline data processing and ensure all replicate data were handled identically, we developed an R script that automated outlier identification, calculation of sample replicate pool average ${\text{Δ}}_{47}$, ${\text{Δ}}_{48}$, $\delta^{18}\text{O}$, and $\delta^{13}\text{C}$ values, total number of replicates (N), replicate pool standard deviation (SD), replicate pool standard error (SE), and normality of the replicate data distribution. A density function was determined for each sample and standard replicate pool on every instrumental configuration after an initial removal of very large outliers (Figure 2b). A 3σ or 5σ (3 SD or 5 SD from the mean) cut was then made for each density function (Figure 2c) to yield the final replicate pool. This method is particularly useful for datasets with a large number of replicates where data processing can be time intensive; it also helps reduce potential human bias. We do not recommend this method for samples with less than 12 replicates, as this was the smallest number of replicates we successfully tested the method on. The sample error reported here as SD and SE, which is typical for clumped isotope measurements, does not fully account for additional error associated with standardizing raw data into the final ${\text{Δ}}_{47}$ values, described as “allogenic” errors by Daëron (2021). These errors likely play a larger role for ${\text{Δ}}_{48}$ given larger measurement uncertainties. However, we report the minimum error contribution from standardization to ${\text{Δ}}_{47}$ (Daëron, 2021) and Δ_48_ values for each instrument configuration.

For data pooling between instrumental configurations, the ${\text{Δ}}_{47}$ and ${\text{Δ}}_{48}$ replicate distributions for standards and samples run on multiple instrument configurations (consistency standards) were directly compared. If no statistically significant differences were observed between configurations, replicates were pooled to calculate a combined average. The ${\text{Δ}}_{48}$ replicate values from the MAT 253 were not pooled with replicate values from the Nu Perspective instruments.

In the Supporting Information S1, we provide a detailed description of this method for replicate-level outlier identification and data pooling from multiple instruments. The R script is publicly available at https://doi.org/10.5281/zenodo.7311624.

### 2.7. Calculation of ${\text{Δ}}_{47}$-*T* and ${\text{Δ}}_{48}$-*T* Equilibrium Relationships Using Acid Fractionation Factors

When carbonate minerals are digested in phosphoric acid, the removal of oxygen atoms from ${\text{CO}}_{3}^{2-}$ depends on the temperature of the reaction and the clumped isotope composition of the reactant mineral (Guo et al., 2009). This removal of oxygen atoms results in a significant increase of the ${\text{Δ}}_{47}$ and ${\text{Δ}}_{48}$ values of the liberated CO_2_ versus the initial ${\text{Δ}}_{63}$ and ${\text{Δ}}_{64}$ values of the mineral (Guo et al., 2009). To account for this difference and its dependence on the clumped isotope composition of the reactant mineral, we determined regression-form AFFs, $\text{Δ}{*}_{63-47}$ and $\text{Δ}{*}_{64-48}$, for when calcite is digested in phosphoric acid at 90°C. The AFFs were determined by first calculating the difference between measured ${\text{Δ}}_{47}$ and ${\text{Δ}}_{48}$ values for samples with known precipitation temperatures at 600 and 33.7°C and theoretical equilibrium ${\text{Δ}}_{63}$ and ${\text{Δ}}_{64}$ values for calcite at 600 and 33.7°C (Hill et al., 2014; Tripati et al., 2015), respectively. The dependence of the AFFs on the initial clumped isotope composition of the mineral was determined by calculating linear regressions between the calculated $\text{Δ}{*}_{63-47}$ and $\text{Δ}{*}_{64-48}$ values for 600 and 33.7°C and the corresponding theoretically predicted ${\text{Δ}}_{63}$ and ${\text{Δ}}_{64}$ values for 600 and 33.7°C (Hill et al., 2014; Tripati et al., 2015), respectively. The measured ${\text{Δ}}_{47}$ and ${\text{Δ}}_{48}$ values used for 600°C were the pooled replicate values for ETH-1 and ETH-2 (Bernasconi et al., 2018), and the values used for 33.7°C were the pooled replicate values for Devils Hole calcite (Coplen, 2007).

The temperature-dependent equilibrium ${\text{Δ}}_{47}$ and ${\text{Δ}}_{48}$ values were then calculated using Equations 9 and 10,

(9) $${\text{Δ}}_{47\;\text{I}-\text{CDES}\;\text{EQ}}={\text{Δ}}_{63}+\text{Δ}{*}_{63-47}$$

(10) $${\text{Δ}}_{48\;\text{CDES}\;90\;\text{EQ}}={\text{Δ}}_{64}+\text{Δ}{*}_{64-48}$$

where ${\text{Δ}}_{63}$ and ${\text{Δ}}_{64}$ values are theoretical equilibrium values for calcite from 0 to 1,000°C (Hill et al., 2014; Tripati et al., 2015), and $\text{Δ}{*}_{63-47}$ and $\text{Δ}{*}_{64-48}$ are the AFFs determined here. A detailed description of this calculation is in Section S3 in Supporting Information S1.

## 3. Results

### 3.1. Statistical Methods

We found no evidence of statistically significant differences in the ${\text{Δ}}_{47}$ or ${\text{Δ}}_{48}$ values of samples analyzed on multiple configurations (Figure S4 in Supporting Information S1; Tables S1 and S2 in Supporting Information S2), thus, replicate analyses from the Nu Perspective instruments were pooled. However, due to higher error, lower precision, and offsets in ${\text{Δ}}_{48}$ values for ETH-1 and ETH-2 that did not exist in data from the Nu Perspective instruments (Table S3 in Supporting Information S2), ${\text{Δ}}_{48}$ replicate data from the MAT 253 was not pooled with Nu Perspective replicate data. Additionally, we have not combined replicate values produced using equilibrated gas-based data normalization with replicate values produced using carbonate-based data normalization.

We found there was a negligible difference in the number of replicates removed when a 3σ versus 5σ cutoff was used for outliers due to narrow peak widths for sample replicate distributions (Figure S5 in Supporting information S1; Table S4 in Supporting Information S2). To further ensure the accuracy of the data presented here, we compared our final ${\text{Δ}}_{47}$ values to Upadhyay et al. (2021) which presented a subset of the data reported here using other methods for outlier removal and data processing (Table S5 in Supporting Information S2). The datasets are in good agreement, with an average offset of 0.011 ‰, despite the ${\text{Δ}}_{47}$ data from their study being normalized differently than the data here, and then being transferred into the I-CDES reference frame using an Equation from Appendix A in Bernasconi et al. (2021).

### 3.2. ${\text{Δ}}_{47}$ and ${\text{Δ}}_{48}$ Results

The ${\text{Δ}}_{47}$ and ${\text{Δ}}_{48}$ values were determined for 7 standards using equilibrated gas-based data normalization, with replicate analyses performed from May 2015 to May 2017 (Table 3). Additionally, ${\text{Δ}}_{47}$ values were determined for 27 standards and samples, and ${\text{Δ}}_{48}$ values for 24 standards and samples using carbonate-based data normalization, with replicate analyses performed from May 2015 to February 2021 (Table 4). All ${\text{Δ}}_{47}$ replicate-level data were normally distributed, with the exception of ETH-3 analyzed on the MAT 253 (Table S6 in Supporting Information S2). All ${\text{Δ}}_{48}$ replicate-level data were normally distributed, with the exception of ETH-1 analyzed on the MAT 253 (Table S7 in Supporting Information S2). We observed that the MAT 253 produced similar sample average ${\text{Δ}}_{48}$ values for the majority of samples, with larger SD and SE than the Nu Perspective instruments (Table S3 in Supporting Information S2; Figure S4 in Supporting Information S1). The δ^18^O and δ^13^C results are presented in Table S8 in Supporting Information S2.

For 1 replicate, the shot noise limits (Huntington et al., 2009; Merritt & Hayes, 1994; Petersen & Schrag, 2014) for ${\text{Δ}}_{47}$ and ${\text{Δ}}_{48}$ values determined on Nu Perspective-EG and Nu Perspective-1 were 0.008 ‰ and 0.027 ‰, respectively. The ${\text{Δ}}_{47}$ and ${\text{Δ}}_{48}$ shot noise limits on Nu Perspective-1a and Nu Perspective-2 range from 0.008–0.013 ‰ and 0.027–0.044 ‰, respectively. The ${\text{Δ}}_{47}$ and ${\text{Δ}}_{48}$ shot noise limits for the MAT 253 were 0.013 ‰ and 0.042 ‰, respectively. Considering the total integration time for each standard and sample (total integration time = number of replicates × integration time), all ${\text{Δ}}_{47}$ and ${\text{Δ}}_{48}$ values determined on the Nu Perspective mass spectrometers had errors (1 SE) that were within ≤0.006 ‰ and ≤0.009 ‰, respectively, of the shot noise limit. The ${\text{Δ}}_{47}$ and ${\text{Δ}}_{48}$ values determined on the MAT 253 had errors (1 SE) that were within ≤0.008 ‰ and ≤0.037 ‰ of the shot noise limit, respectively.

For the Nu Perspective mass spectrometers, ∼9 replicates were required to reach a ${\text{Δ}}_{48}$ mean value within the shot noise limit bounds (Figure S6 in Supporting Information S1). For the MAT 253, ∼25 replicates were required to reach a ${\text{Δ}}_{48}$ mean value within the shot noise limit bounds (Figure S6 in Supporting Information S1). Additionally, the minimum error contribution to ${\text{Δ}}_{47}$ values from standardization (Daëron, 2021) were 0.000 ‰, 0.002 ‰, 0.001 ‰, 0.001 ‰ for Nu Perspective-EG, Nu Perspective-1, Nu Perspective-2, and MAT 253, respectively. The minimum error contribution to ${\text{Δ}}_{48}$ values from standardization were 0.005 ‰, 0.004 ‰, 0.002 ‰, 0.004 ‰ for Nu Perspective-EG, Nu Perspective-1, Nu Perspective-2, and MAT 253, respectively.

#### 3.2.1. Experimentally Determined ${\text{Δ}}_{47}$–${\text{Δ}}_{48}$ Regression

**Figure 3.**
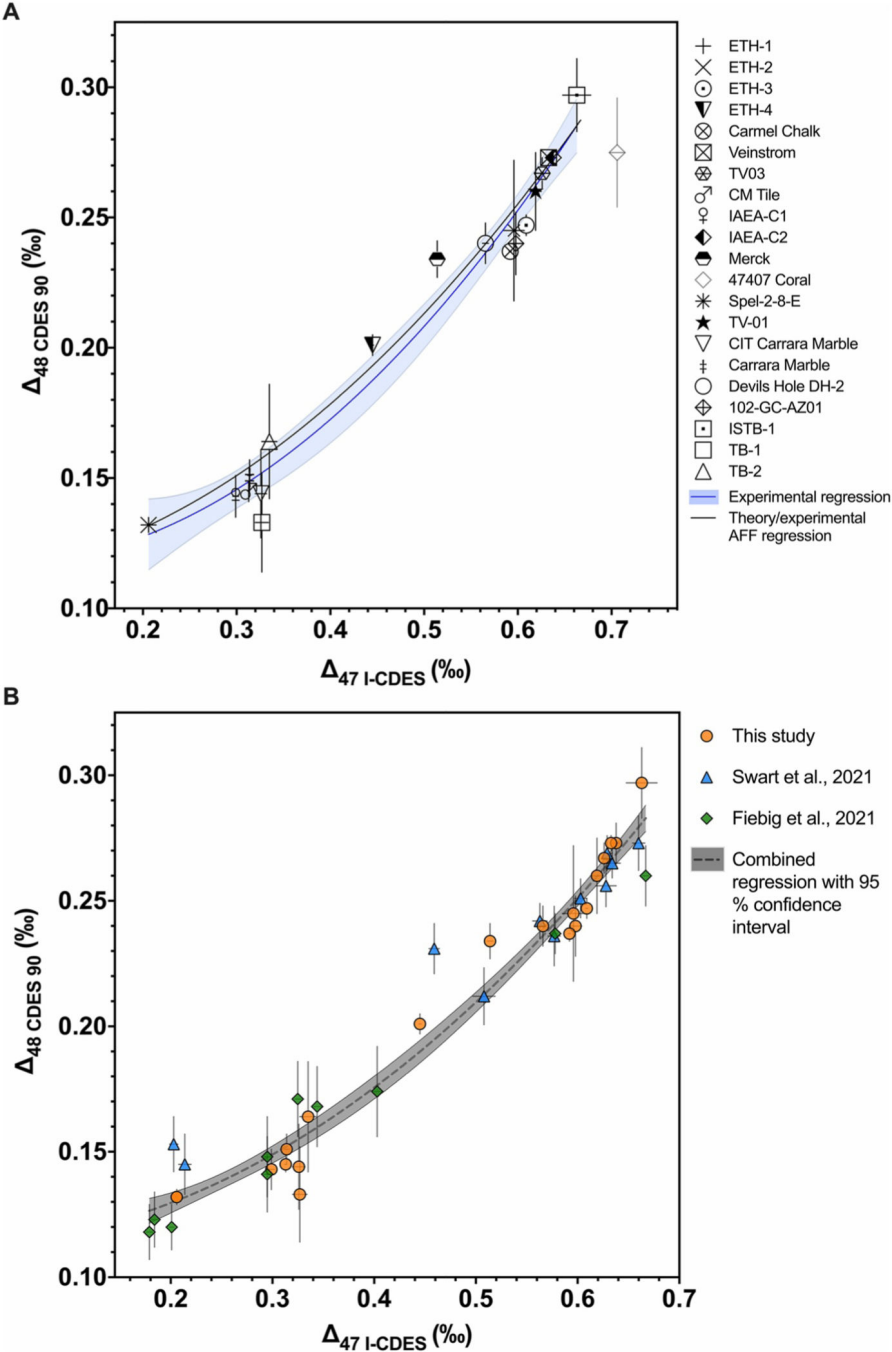
(a) Plot showing ${\text{Δ}}_{47}$–${\text{Δ}}_{48}$ values for 21 samples including standards and Devils Hole (DH-2) cave calcite. A polynomial (blue line) was fitted through all samples and standards, with the exception of 47407 Coral, which may express kinetic bias. The light blue shading indicates the 95% confidence interval. Also shown is a calculated equilibrium regression (black line) determined using theoretical calcite equilibrium ${\text{Δ}}_{63}$ and ${\text{Δ}}_{64}$ values (Hill et al., 2014; Tripati et al., 2015) combined with AFFs to determine ${\text{Δ}}_{47}$–${\text{Δ}}_{48}$ values. Error bars indicate 1 SE. (b) Experimental ${\text{Δ}}_{47}$–${\text{Δ}}_{48}$ data from this study (orange circles), Swart et al. (2021) (blue triangles), and Fiebig et al. (2021) (green diamonds). The data from this study are the same as for panel A, with the exception of 47407 Coral. A combined data regression (gray dashed line) was determined including all three datasets. The gray shading indicates the 95% confidence interval.

A polynomial, Equation 11 (*r*^2^ = 0.97), was fit through experimentally determined ${\text{Δ}}_{47}$ and ${\text{Δ}}_{48}$ values for 20 standards and samples, including Devils Hole calcite (Figure 3a).


(11) $${\text{Δ}}_{48\;\text{CDES}\;90}=(0.1179\pm 0.0266)-(0.0398\pm 0.1332){\text{Δ}}_{47\;\text{I}-\text{CDES}}+(0.4407\pm 0.1490){{\text{Δ}}_{47\;\text{I}-\text{CDES}}}^{2}$$

All ${\text{Δ}}_{47}$ and ${\text{Δ}}_{48}$ values used to calculate Equation 11 can be found in Table 4. Of the 21 total samples in Figure 3a, all lie within 1 SE of the 95% confidence interval of the regression, with the exception of Merck, Carmel Chalk, and 47407 Coral. 47407 Coral was the only sample excluded from Equation 11 due to the apparent influence of kinetic isotope effects on the ${\text{Δ}}_{47}$ and ${\text{Δ}}_{48}$ values, which resulted in an offset of >1 SD from the regression.

### 3.3. Calculated ${\text{Δ}}_{47}$-*T*, ${\text{Δ}}_{48}$-*T*, and ${\text{Δ}}_{47}$–${\text{Δ}}_{48}$ Regressions

The calculated equilibrium ${\text{Δ}}_{47}$ and ${\text{Δ}}_{48}$ values for 0–1,000°C are in Table S9 in Supporting Information S2. The ${\text{Δ}}_{47}$–${\text{Δ}}_{48}$ relationship (black line, Figure 3a) is represented by Equation 12.


(12) $${\text{Δ}}_{48\;\text{CDES}\;90\;\text{EQ}}=0.1123+0.01971{\text{Δ}}_{47\text{I}-\text{CDES}\;\text{EQ}}+0.364{{\text{Δ}}_{47\text{I}-\text{CDES}\;\text{EQ}}}^{2}$$

The ${\text{Δ}}_{47}$-*T* and ${\text{Δ}}_{48}$-*T* relationships (Figure 6) are described by Equations 13 (r^2^ = 0.99) and 14 (r^2^ = 0.99).

(13) $${\text{Δ}}_{47\;\text{I}-\text{CDES}\;\text{EQ}}=0.1491-0.1308(1/T)+39102{\left(1/T\right)}^{2}$$

(14) $${\text{Δ}}_{48\;\text{CDES}\;90\;\text{EQ}}=0.1715-62.3(1/T)+25590{\left(1/T\right)}^{2}$$

where temperature is in Kelvin.

The AFFs for the compositionally-dependent fractionation of O isotopes during phosphoric acid digestion of carbonate minerals (Figure S1 in Supporting Information S1) are represented by Equations 15 and 16,

(15) $${\text{Δ}}_{63-47}^{*}=0.0190\times {\text{Δ}}_{47\;\text{I}-\text{CDES}}+0.1842$$

(16) $${\text{Δ}}_{64-48}^{*}=0.0077\times {\text{Δ}}_{48\;\text{CDES}\;90}+0.1290$$

where $\text{Δ}{*}_{63-47}$ and $\text{Δ}{*}_{64-48}$ are the AFFs.

## 4. Discussion

### 4.1. Comparison of ${\text{Δ}}_{47}$ and ${\text{Δ}}_{48}$ Values Determined With Equilibrated Gas-Based Data Normalization to Previously Published Results

**Figure 4.**
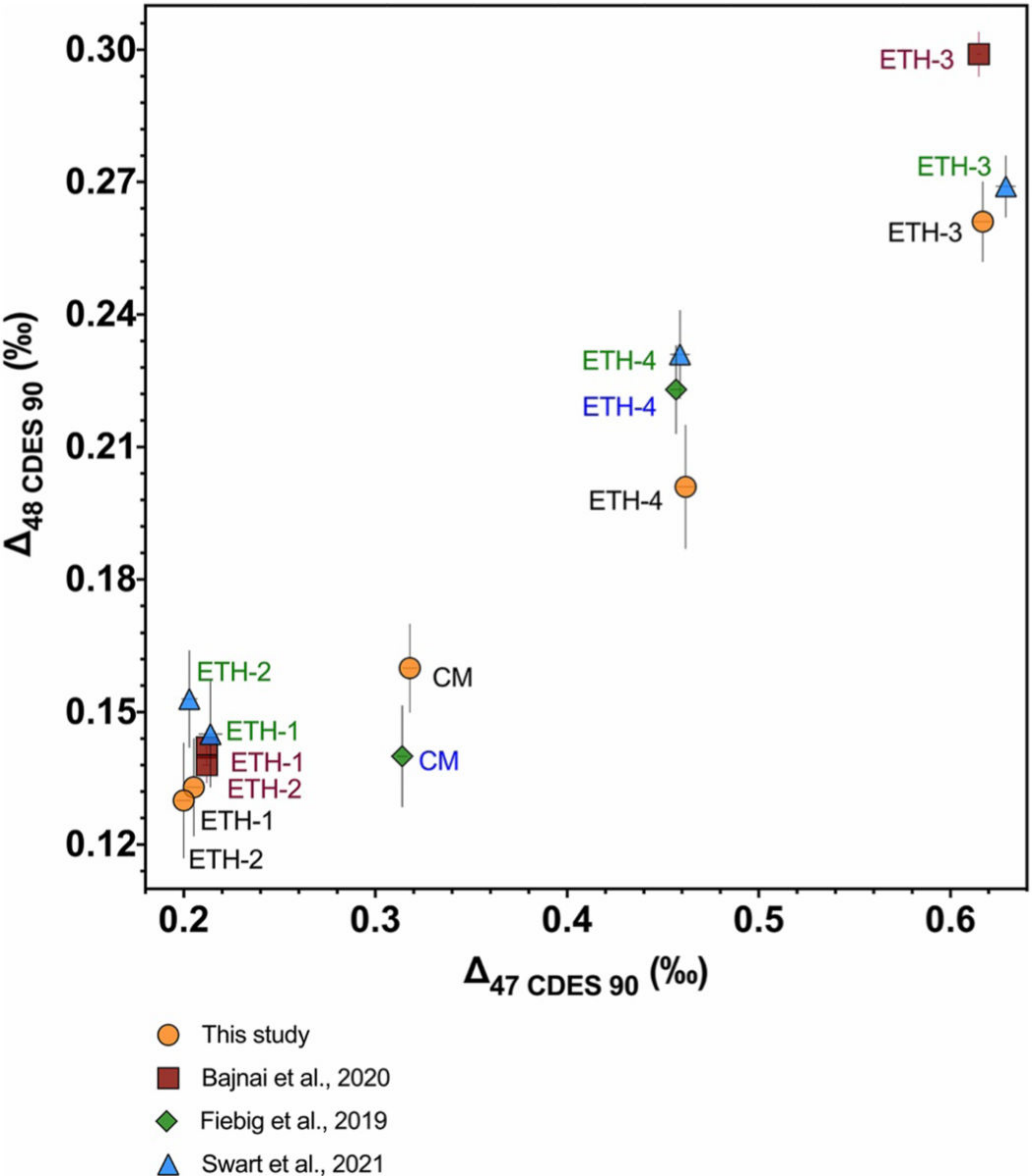
Plot showing the comparison between ${\text{Δ}}_{47}$–${\text{Δ}}_{48}$ values determined with 25 and 1,000°C equilibrated gas-based data normalization for ETH-1, ETH-2, ETH-3, ETH-4, and Carrara Marble (CM) from this study (orange circles), Fiebig et al. (2019) (green diamonds), Bajnai et al. (2020) (dark red squares), and Swart et al. (2021) (blue triangles). Error bars indicate 1 SE.

Since the accurate determination of ${\text{Δ}}_{48}$ is a relatively new method, the development of robust standard values is of the utmost importance to ensure intra- and inter-laboratory reproducibility. To establish carbonate standard ${\text{Δ}}_{48}$ values that can be used in data normalization for unknown samples, ${\text{Δ}}_{48}$ values for carbonate standards must first be determined relative to equilibrated gases. We have compared our ${\text{Δ}}_{47}$ and ${\text{Δ}}_{48}$ values for carbonate standards determined using equilibrated gas-based data normalization to other recently published datasets with paired clumped isotope values for ETH standards, including Fiebig et al. (2019), Bajnai et al. (2020), and Swart et al. (2021) (Figure 4, Table 3). There is good interlaboratory agreement for ${\text{Δ}}_{47}$ values, with a range of 0.002–0.012 ‰ for ${\text{Δ}}_{47}$ offsets for replicated samples. The ${\text{Δ}}_{47}$ error, reported as 1 SE, was similar (0.001–0.006 ‰) for all studies. When the ${\text{Δ}}_{47}$ values for carbonate standards determined in these studies were compared to the multi-laboratory study from Bernasconi et al. (2021) which determined nominal ${\text{Δ}}_{47}$ values for carbonate standards, there was similar agreement between laboratories, with offsets from 0.000–0.012 ‰ for replicated samples (Table 3). The interlaboratory ${\text{Δ}}_{48}$ offsets were larger, with a range of 0.008–0.038 ‰ for replicated samples, although the majority of replicated samples were with within 1 SE of each other (Table 3, Figure 4). The ${\text{Δ}}_{48}$ error reported in Bajnai et al. (2020) of 0.004–0.005 ‰ was lower than that for the other studies which have error ranging from 0.007–0.014 ‰. Bajnai et al. (2020) used larger sample size (10 mg compared to 5 mg in this study), and longer mass spectrometric integration times than what was used here, which result in better counting statistics. Fiebig et al. (2019) reported a shot noise limit of 0.027 ‰ for ${\text{Δ}}_{48}$ for conditions similar to what was used in Bajnai et al. (2020), while the ${\text{Δ}}_{48}$ shot noise limit for the Nu Instruments in this study range from 0.027–0.044 ‰. Further, the average interlaboratory ${\text{Δ}}_{48}$ offset was 0.019 ‰ (taken as the average of the absolute value of offsets of replicated samples in Table 3). These offsets are likely from random error, considering that the *m/z* 48 isotopologue is an order of magnitude lower in abundance than the *m/z* 47 isotopologue (Ghosh et al., 2006), and the offsets are within the shot noise limits.

The use of equilibrated gases for data normalization has been shown to be a potential source of error and interlaboratory offsets since the sample undergoes acid digestion and the gas standard does not, different laboratories use different setups to produce gas standards, and fractionations may occur from quenching during the production of heated gas standards (Bernasconi et al., 2018). However, interlaboratory ${\text{Δ}}_{47}$ offsets up to 0.024 ‰ in Bernasconi et al. (2021) were determined to be the result of random error which may be amplified during data normalization. The range in ${\text{Δ}}_{47}$ offsets observed here are smaller than what was observed between laboratories reported in Bernasconi et al. (2021), possibly from overall high replication.

### 4.2. Carbonate-Based Data Normalization of ${\text{Δ}}_{47}$–${\text{Δ}}_{48}$ Measurements

Previously, important contributions have demonstrated that carbonate standard-based data normalization that uses readily available materials can produce robust ${\text{Δ}}_{47}$ values and yield interlaboratory discrepancies that are consistent with analytical uncertainties (Bernasconi et al., 2018, 2021; Meckler et al., 2014). We applied this approach, using ETH-1, ETH-2, and ETH-3 as carbonate standards on multiple instruments in our laboratory for the paired analysis of ${\text{Δ}}_{47}$–${\text{Δ}}_{48}$. The combined instrument average from this study (Table 4) and Bernasconi et al. (2021) had excellent agreement between ${\text{Δ}}_{47}$ values for samples used as unknowns in both studies, with offsets of 0.005‰, 0.003‰, 0.003‰, 0.001‰ for ETH-4, IAEA-C1, IAEA-C2, and Merck, respectively. This is likely because the nominal ${\text{Δ}}_{47}$ values determined in Bernasconi et al. (2021) for ETH-1, ETH-2, and ETH-3 were used here in transfer functions for data normalization, adding supporting evidence for the importance of laboratories using common standard values to improve reproducibility.

**Figure 5.**
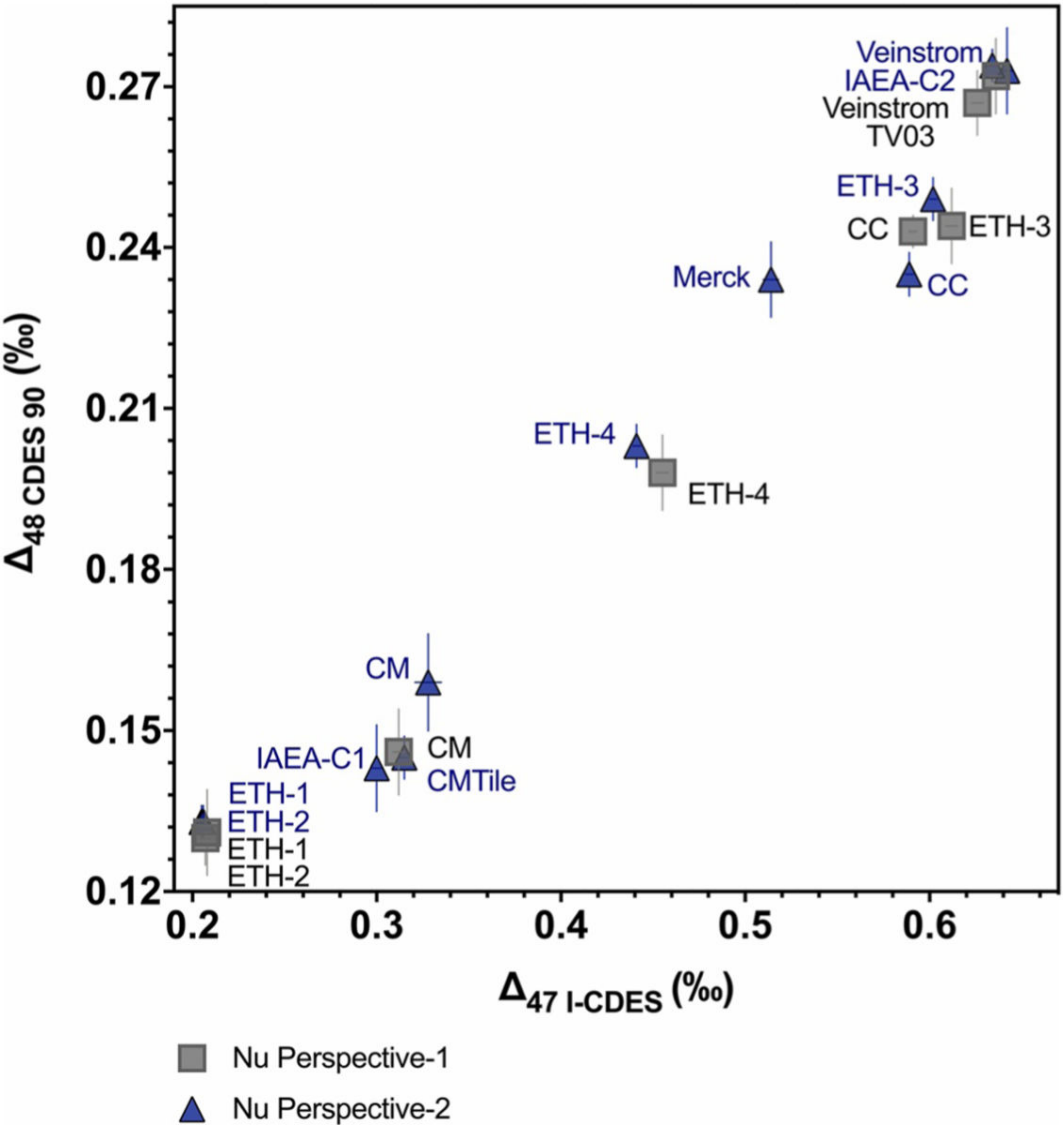
The ${\text{Δ}}_{47}$–${\text{Δ}}_{48}$ values for standards and samples determined using carbonate-based data normalization on two mass spectrometers, Nu Perpective-1 (gray squares) and Nu Perspective-2 (blue triangles). CC is Carmel Chalk, CM is Carrara Marble, and CM Tile is Carrara Marble Tile. Error bars indicate 1 SE.

Similarly, carbonate standard-based data normalization yielded reproducible ${\text{Δ}}_{48}$ results across two Nu Perspective instruments, Nu Perspective-1 and Nu Perspective-2 (Figure 5, Table S3 in Supporting Information S2). Consistent with Daëron (2021) and Kocken et al. (2019), we recommend a 50:50 sample to standard ratio, which was what was utilized here. The ${\text{Δ}}_{48}$ offsets between instruments ranged from 0.004–0.013 ‰ for the 3 samples treated as unknowns (consistency standards), Carrara Marble, CM Tile, and ETH-4. These offsets were reduced compared to interlaboratory ${\text{Δ}}_{48}$ offsets observed for ETH standards and Carrara Marble determined using equilibrated gas-based data normalization (average: 0.018 ‰; minimum: 0.008 ‰; maximum: 0.038 ‰).

We also present ${\text{Δ}}_{48}$ data determined on the older generation Thermo MAT 253. We decided to include these data due to the large amount of clumped isotope data produced on this instrument going back to 2014 and given comments from J. Eiler (pers. comm.) indicating these instruments may produce useable ${\text{Δ}}_{48}$ data. We sought to test as to whether this instrument, with sufficient replication and quality control, could yield reproducible ${\text{Δ}}_{48}$ values. The MAT 253 produced similar sample average ${\text{Δ}}_{48}$ values when compared to the Nu Perspective Instruments for the majority of samples (Table S3 in Supporting Information S2). The decision was made to not pool the ${\text{Δ}}_{48}$ values produced on the MAT 253 due to lower external precision relative to the Nu Instruments (average 1 SD error for MAT 253 = 0.105 ‰; average 1 SD error for Nu Perspective instruments = 0.056 ‰), more noise and smaller overall peaks observed in the ${\text{Δ}}_{48}$ peak-shapes relative to the Nu Perspective instruments (Figure S2 in Supporting Information S1), and the large offset (0.017 ‰) between the ${\text{Δ}}_{48}$ values for ETH-1 and ETH-2 determined on the MAT 253 (Table S3 in Supporting Information S2), which was not observed on the Nu Perspective instruments. However, it may be worth mining past MAT 253 datasets to examine ${\text{Δ}}_{48}$ depending on the reproducibility of measurements, although newer generation instrumentation is preferable for the measurement of ${\text{Δ}}_{48}$ values due to significantly improved precision.

### 4.3. ${\text{Δ}}_{47}$–${\text{Δ}}_{48}$ Equilibrium Regression Using Samples and Standards

We report a ${\text{Δ}}_{47}$–${\text{Δ}}_{48}$ regression (Equation 11) for 20 carbonate standards and samples (combined average values in Table 4). To have a constraint as to whether the materials included in the regression achieved quasi-equilibrium clumped isotope values, we compared the experimental regression to a regression based on theoretical calcite equilibrium (Figure 3a). The theoretical regression for ${\text{Δ}}_{63}$–${\text{Δ}}_{64}$ equilibrium was transferred into ${\text{Δ}}_{47}$–${\text{Δ}}_{48}$ space using AFFs (Equations 15 and 16). When the experimental regression was compared to the theoretically based regression, they were found to be statistically indistinguishable (*P* = 0.39; *F* = 1.03; Table S10 in Supporting Information S2). This supports the assumption that the materials used in the experimental regression have achieved quasi-equilibrium clumped isotope values.

All sample and standard ${\text{Δ}}_{47}$ and ${\text{Δ}}_{48}$ values are within 1 SE of the 95% confidence interval of the regression (Equation 11; Figure 3a), with the exception of Merck, Carmel Chalk, and 47407 Coral. The 47407 Coral was the only sample not included in the regression. The possibility that Merck, an ultra-pure synthetic calcite, and Carmel Chalk, a natural calcite chalk, are exhibiting subtle clumped isotope disequilibrium cannot be excluded. However, 47407 Coral is a deep-sea coral of the genus *Desmophyllum* with an estimated growth temperature of 4.2°C (Thiagarajan et al., 2011). Guo (2020) used model estimates to predict a negative correlation between ${\text{Δ}}_{47}$ and ${\text{Δ}}_{48}$ values for cold-water corals, with kinetic effects causing enrichments in ${\text{Δ}}_{47}$ values and depletions in ${\text{Δ}}_{48}$ values. We determined that the 47407 Coral exhibits an enrichment of 0.030 ‰ in ${\text{Δ}}_{47}$ and depletion of −0.018 ‰ in ${\text{Δ}}_{48}$ by defining nominal equilibrium as the regression through the remaining samples, and the offsets were determined by using a kinetic slope for CO_2_ absorption in corals of −0.6 (Bajnai et al., 2020; Guo, 2020). Bajnai et al. (2020) also measured ${\text{Δ}}_{47}$ and ${\text{Δ}}_{48}$ values for a coral of the same genus (*Desmophyllum*) and a brachiopod (*Magellania venosa*) and observed similar enrichments in ${\text{Δ}}_{47}$ (0.038–0.069 ‰) and depletions in ${\text{Δ}}_{48}$ (−0.0004 to −0.095 ‰).

### 4.4. Constraining Equilibrium ${\text{Δ}}_{47}$–${\text{Δ}}_{48}$

The equilibrium ${\text{Δ}}_{47}$–${\text{Δ}}_{48}$ relationship is of recent interest due to the potential for use in identifying kinetic effects in biotic and abiogenic carbonate minerals that are or could be used for paleotemperature reconstructions. A study (Bajnai et al., 2020) used a kinetic slope calculated relative to a proposed equilibrium ${\text{Δ}}_{47}$–${\text{Δ}}_{48}$ regression to recover temperature signals in kinetically controlled samples. To further develop the use of ${\text{Δ}}_{47}$–${\text{Δ}}_{48}$ equilibrium as a proxy to identify kinetic effects, the ${\text{Δ}}_{47}$–${\text{Δ}}_{48}$ equilibrium relationship must be well constrained. Thus, we compared the experimentally determined ${\text{Δ}}_{47}$–${\text{Δ}}_{48}$ regressions for quasi-equilibrium materials determined here (Equation 11) to those from Swart et al. (2021) and Fiebig et al. (2021) using a sum-of-squares F test (Table S10 in Supporting Information S2). This compares the fit of a regression through all datasets to the fit of individual regressions for each data set, and tests whether the datasets differ sufficiently from each other to warrant separate regressions. The data set from Swart et al. (2021) contains 7 calcite precipitations in 5°C increments from 5–65°C and carbonate standards ETH-1, ETH-2, ETH-3, and ETH-4. The data set from Fiebig et al. (2021) includes 16 samples, some of which are combined into averages, yielding 10 samples that are used for comparison here, including lake calcite, Devils Hole calcite, calcite precipitations, and calcite equilibrated at high temperatures, with crystallization temperatures for all samples ranging from 8–1,100°C. We found no evidence of statistically significant differences between the individual regressions (*P* = 0.86; *F* = 0.43; Table S10 in Supporting Information S2), and we therefore produced a combined regression, described by Equation 17, which is composed of 41 samples that are believed to have achieved quasi-equilibrium clumped isotope values (Figure 3b).


(17) $${\text{Δ}}_{48\;\text{CDES}\;90}=(0.1132\pm 0.010)+(0.008\pm 0.055){\text{Δ}}_{47\;\text{CDES}\;90}+(0.3692\pm 0.065){{\text{Δ}}_{47\;\text{CDES}\;90}}^{2}$$

Of the 41 samples used in Equation 17, 35 are within 1 SE of the 95% confidence interval. The samples outside of this threshold include Carmel Chalk, ETH-4, and Merck from this study; ETH-2 and ETH-4 from Swart et al. (2021); and a cave calcite sample from Fiebig et al. (2021). It is unlikely that ETH-2 is exhibiting kinetic effects since it has an equilibration temperature of 600°C (Bernasconi et al., 2018), and has near stochastic isotopic values (Müller et al., 2017). The cave calcite sample from Fiebig et al. (2021) is from Laghetto Basso, Italy with a precipitation temperature of 7.9 ± 0.2°C. Fiebig et al. (2021) and Daëron et al. (2019) argued that this sample precipitated close to equilibrium due to long residence times of water in the lake, low calcite saturation index (<0.3), slow precipitation rate (0.3 μm/yr), and consistent $\delta^{18}\text{O}$ values for contemporaneously deposited calcite layers. It cannot be ruled out that ETH-4, the same commercially available calcite as ETH-2 but unheated (Bernasconi et al., 2018), exhibits subtle kinetic effects. The ETH-4 sample from this study is much closer to the equilibrium regression than the ETH-4 sample from Swart et al. (2021), mostly due to offsets in the ${\text{Δ}}_{48}$ value (0.030 ‰) between the studies, which is larger than the offset for ${\text{Δ}}_{47}$ (0.014 ‰). Both the ${\text{Δ}}_{47}$ and ${\text{Δ}}_{48}$ offsets between the studies are within the threshold of observed scatter from random error (Bernasconi et al., 2021). It is also possible that different data normalization methods, carbonate-based here and equilibrated gas-based in Swart et al. (2021), contribute to the difference in ETH-4 values. As discussed above in Section 4.3, it also cannot be ruled out that Carmel Chalk from this study exhibits subtle kinetic effects. However, the scatter for all samples and standards around the equilibrium line are well within what is expected from random error (Bernasconi et al., 2021). Further, the lack of statistical differences between the combined experimental regression (Equation 17) and the theoretically based equilibrium regression (Equation 12), support that Equation 17 is a robust experimental representation of ${\text{Δ}}_{47}$–${\text{Δ}}_{48}$ equilibrium. Samples with ${\text{Δ}}_{47}$–${\text{Δ}}_{48}$ values that deviate significantly from this relationship may have non-equilibrium clumped isotope signatures.

### 4.5. Acid Digestion Fractionation Factors

The values for AFFs, $\text{Δ}{*}_{63-47}$ and $\text{Δ}{*}_{64-48}$, for when O atoms are cleaved from ${\text{CO}}_{3}^{2-}$ during phosphoric acid digestion at 90°C, are useful for comparison of measured ${\text{Δ}}_{47}$ and ${\text{Δ}}_{48}$ values and theoretical ${\text{Δ}}_{63}$ and ${\text{Δ}}_{64}$ values. The direct measurement of carbonate mineral ${\text{Δ}}_{63}$ and ${\text{Δ}}_{64}$ is currently not possible. The AFFs can be used to estimate calcite ${\text{Δ}}_{63}$ and ${\text{Δ}}_{64}$ values via Equations 9 and 10. Model calculations from Guo et al. (2009) predicted that these AFFs should depend on the ${\text{Δ}}_{63}$ and ${\text{Δ}}_{64}$ values of the reactant carbonate mineral. Our data indicates that the use of a regression-form AFF versus a constant AFF may be important for ${\text{Δ}}_{47}$ and has only a negligible effect on ${\text{Δ}}_{48}$, as there is a ∼0.009 ‰ difference in $\text{Δ}{*}_{63-47}$ from 0–600°C, while there is only a ∼0.001 ‰ difference in $\text{Δ}{*}_{64-48}$ over the same temperature range (Table S9 in Supporting Information S2).

### 4.6. Constraining Equilibrium ${\text{Δ}}_{47}$-*T* and ${\text{Δ}}_{48}$-*T*

To date, 3 groups have published relationships for both ${\text{Δ}}_{47}$-*T* and ${\text{Δ}}_{48}$-*T*. The regressions from Swart et al. (2021) and Fiebig et al. (2021) are based on measured values from calcite precipitated/equilibrated at quasi-equilibrium, while the regressions from this study and Bajnai et al. (2020) are based on a combination of theoretical calcite mineral ${\text{Δ}}_{63}$–${\text{Δ}}_{64}$ equilibrium values, which were transformed into ${\text{Δ}}_{47}$–${\text{Δ}}_{48}$ space using AFFs (see Methods 2.7). The ${\text{Δ}}_{47}$-*T* and ${\text{Δ}}_{48}$-*T* regressions from Bajnai et al. (2020) were calculated for 0–40°C, while the experimentally based regressions from Swart et al. (2021) are for 0–65°C. In this study we calculated ${\text{Δ}}_{47}$-*T* and ${\text{Δ}}_{48}$-*T* values from 0–1,000°C, and Fiebig et al. (2021) has experimentally constrained values from 8–1,100°C. Due to the regressions from this study and Bajnai et al. (2020) being theoretically based and therefore difficult to accurately provide an error calculation, we were unable to perform the same type of statistical analysis to compare regressions, as we did for the experimental ${\text{Δ}}_{47}$–${\text{Δ}}_{48}$ regressions. Instead, we have compared the absolute difference between the regressions over a wide temperature range, at 0 and 600°C, and compared this difference to measurement error observed in standards replicated between the laboratories, as well as offsets expected from random error. We used these metrics to determine if it was appropriate to determine a combined regression. For ${\text{Δ}}_{47}$-*T*, the largest offset at 0°C was 0.002 ‰ between this study and Bajnai et al. (2020). The offset at 600°C was 0.005 ‰ between this study and Fiebig et al. (2021). For ${\text{Δ}}_{48}$-*T*, the largest offset at 0°C was 0.012 ‰ between this study and Bajnai et al. (2020). The offset at 600°C was 0.007 ‰ between this study and Fiebig et al. (2021).

**Figure 6.**
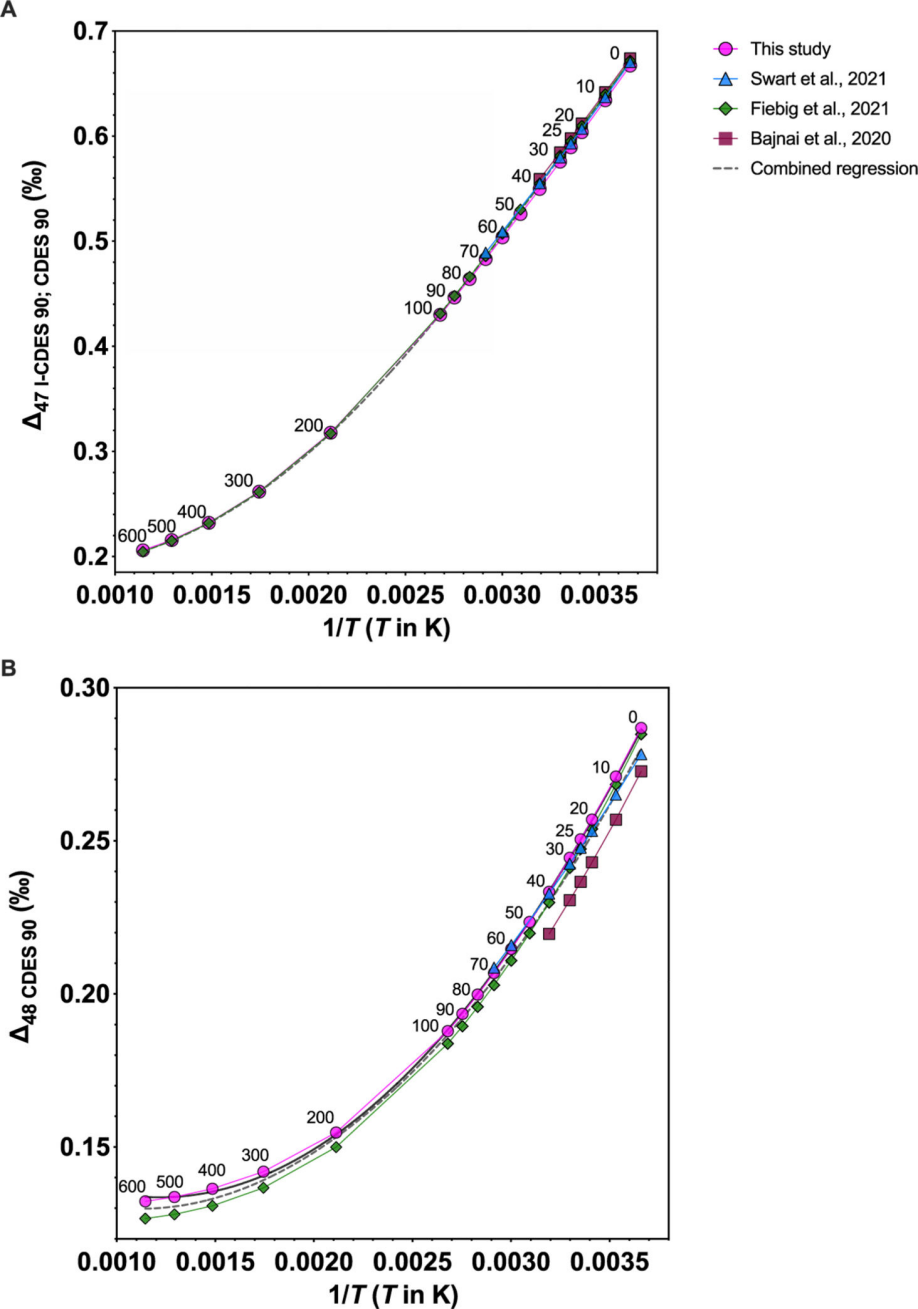
(a) ${\text{Δ}}_{47}$-*T* and (b) ${\text{Δ}}_{48}$-*T* regressions from this study (pink line), Bajnai et al. (2020) (dark red line), Swart et al. (2021) (blue line), and Fiebig et al. (2021) (green line). A combined regression for all datasets is represented by the gray dashed line. Numbers by the calculated data points indicate temperature in Celsius.

These offsets are well within the bounds of what we observed when comparing differences between ETH standard ${\text{Δ}}_{47}$ and ${\text{Δ}}_{48}$ values between laboratories (Figure 4). This is a good metric for interlaboratory analytical error due to large numbers of replicates of ETH standards in all groups. The offsets are also within the bounds expected from random error in ${\text{Δ}}_{47}$ measurements (Bernasconi et al., 2021). Therefore, we determined combined regressions for ${\text{Δ}}_{47}$-*T* and ${\text{Δ}}_{48}$-*T* (Figure 6), represented by Equations 18 (r^2^ = 0.99) and 19 (r^2^ = 0.99),

(18) $${\text{Δ}}_{47\;\text{I}-\text{CDES};\text{CDES}\;90}=0.2017-36.2\times (1/T)+16822\times {\left(1/T\right)}^{2}+18878240\times {\left(1/T\right)}^{3}-3064202063\times {\left(1/T\right)}^{4}$$

(19) $${\text{Δ}}_{48\;\text{CDES}\;90}=0.1642-64.1\times (1/T)+32920\times {\left(1/T\right)}^{2}-3140075\times {\left(1/T\right)}^{3}+354396957\times {\left(1/T\right)}^{4}$$

where *T* is in Kelvin. We also report the inverse of the relationships for ease of use for samples with unknown precipitation temperature in Equations 20 and 21.


(20) $$1/T=-0.003728+0.04027\;{\text{Δ}}_{47\;\text{I}-\text{CDES};\text{CDES}\;90}-0.1048{{\text{Δ}}_{47\;\text{I}-\text{CDES};\text{CDES}\;90}}^{2}\;+0.134{{\text{Δ}}_{47\;\text{I}-\text{CDES};\text{CDES}\;90}}^{3}-0.06386{{\text{Δ}}_{47\;\text{I}-\text{CDES};\text{CDES}\;90}}^{4}$$

(21) $$1/T=-0.02296+0.425\;{\text{Δ}}_{48\;\text{CDES}\;90}-2.718{{\text{Δ}}_{48\;\text{CDES}\;90}}^{2}+7.936{{\text{Δ}}_{48\;\text{CDES}\;90}}^{3}-8.704{{\text{Δ}}_{48\;\text{CDES}\;90}}^{4}$$

To further check the robustness of the combined equilibrium relationships, we solved the ${\text{Δ}}_{47}$-*T* equation (Equation 18) for 10°C, yielding a ${\text{Δ}}_{47}$ value of 0.639 ‰. Then, solved the ${\text{Δ}}_{47}$–${\text{Δ}}_{48}$ regression (Equation 17) for ${\text{Δ}}_{48}$ using 0.639 ‰ as the input ${\text{Δ}}_{47}$. This returned a ${\text{Δ}}_{48}$ value of 0.269 ‰, which is offset by 0.004 ‰ from the ${\text{Δ}}_{48}$ value obtained when solving the ${\text{Δ}}_{48}$-*T* equation (Equation 17) for 10°C. While it may seem obvious that these equations would have good agreement, this may not necessarily have been the case given the large amount of data determined here for samples and standards that contributed to the experimental ${\text{Δ}}_{47}$–${\text{Δ}}_{48}$ regression not having constrained relationships to temperature, and were therefore not used in the ${\text{Δ}}_{47}$-*T* and ${\text{Δ}}_{48}$-*T* regressions. Additionally, the theoretically-based ${\text{Δ}}_{47}$ and ${\text{Δ}}_{48}$ values from Bajnai et al. (2020) and this study were not included in the experimentally based ${\text{Δ}}_{47}$–${\text{Δ}}_{48}$ regression. Thus, the excellent agreement between the combined-laboratory regressions for ${\text{Δ}}_{47}$–${\text{Δ}}_{48}$, ${\text{Δ}}_{47}$-*T*, and ${\text{Δ}}_{48}$-*T* provides evidence that Equations 17–21 are robust representations of clumped isotope equilibrium relationships.

### 4.7. Comparison of Devils Hole ${\text{Δ}}_{47}$ and ${\text{Δ}}_{48}$

There are multiple lines of evidence that Devils Hole calcite has achieved quasi-equilibrium oxygen and clumped isotope values (Bajnai et al., 2021; Coplen, 2007). It has a well-constrained precipitation temperature of 33.7 ± 0.2°C (Dudley & Larson, 1976; Hoffman, 1988; Miller, 1948; Plummer et al., 2000). Therefore, samples from Devils Hole have been used to anchor clumped isotope equilibrium regressions (Bajnai et al., 2020; Fiebig et al., 2021; Tripati et al., 2015), including in this study. To further constrain and compare ${\text{Δ}}_{47}$ and ${\text{Δ}}_{48}$ values for Devils Hole calcite from multiple cores, replicate-level values from this study were compared to previously published replicate-level values from Bajnai et al. (2021) and Fiebig et al. (2021). This study used 4 samples from core DH-2, spanning 146–176 ka (Winograd et al., 1992). Bajnai et al. (2021) used 10 samples from cores DH-11, DHC2–8, and DHC2–3, spanning 4.5–508 ka. Fiebig et al. (2021) used 4 samples from core DHC2–8, all of which were dated to 4.5–16.9 ka. The replicate pools from these three studies were compared using an ANOVA (Table S11 in Supporting Information S2), which is a statistical test comparing whether population means are significantly different. The mean Δ_48_ values from all three studies were statistically indistinguishable (*P* = 0.71; *F* = 0.34; Table S11 in Supporting Information S2). In contrast, the mean ${\text{Δ}}_{47}$ values from this study and Bajnai et al. (2021) were significantly different (*P* < 0.0001), as were mean ${\text{Δ}}_{47}$ values from this study and Fiebig et al. (2021) (*P* < 0.0001). The Devils Hole mean ${\text{Δ}}_{47}$ value offset between this study and Bajnai et al. (2021) is 0.012 ‰, and 0.018 ‰ between this study and Fiebig et al. (2021) (Table 5). For comparison, the largest offset in replicated standard values between this study, Fiebig et al. (2019), and Bajnai et al. (2020) was 0.012 ‰ (Table 3), thus, the observed offset in Devils Hole ${\text{Δ}}_{47}$ values could be from analytical error.

**Figure 7.**
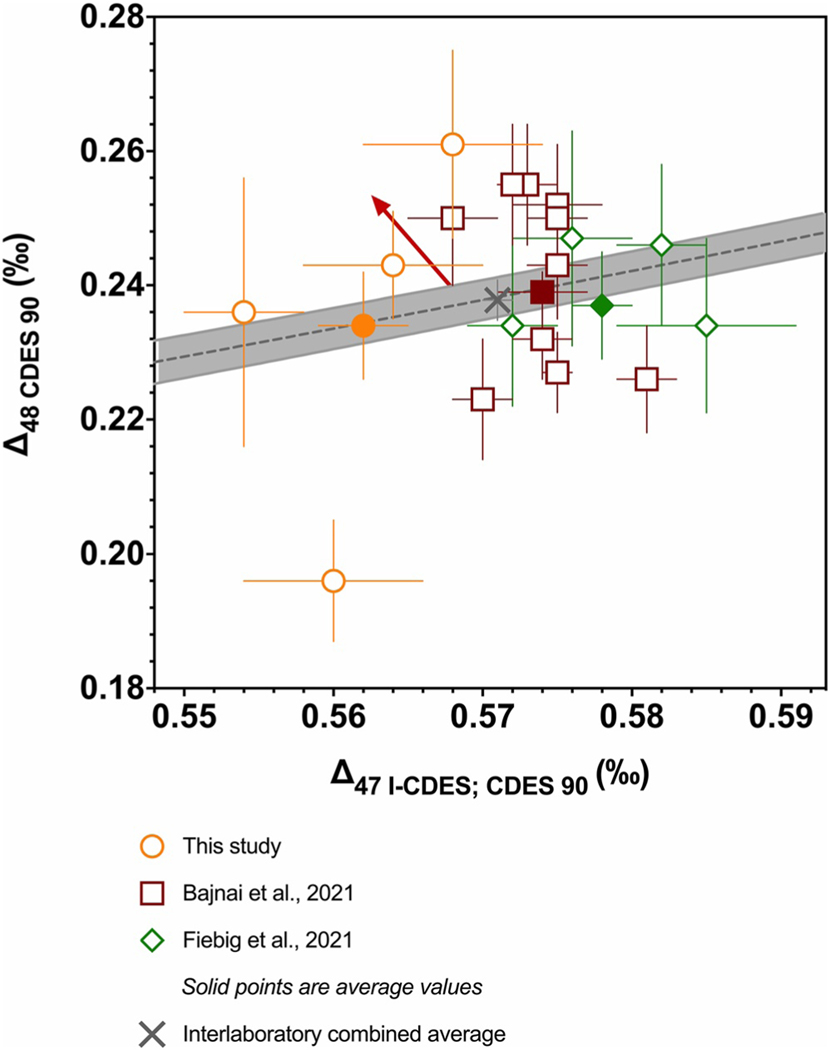
Plot showing ${\text{Δ}}_{47}$–${\text{Δ}}_{48}$ values for Devils Hole cave calcite determined in this study (orange circles), Bajnai et al. (2021) (dark red squares), and Fiebig et al. (2021) (green diamonds). The open points indicate individual samples, and solid points are the overall average from each study. The gray X is the combined average from all datasets. The gray dashed line is the combined experimental equilibrium regression (Equation 17), with the 95% confidence interval indicated by the gray band. Errors bars indicate 1 SE.

It is unlikely that the offsets are the result of Devils Hole samples exhibiting kinetic effects from CO_2_ degassing from groundwater, which is observed in other speleothems (Affek et al., 2008; Affek & Zaarur, 2014; Daëron et al., 2011; Guo, 2020; Guo & Zhou, 2019; Kluge & Affek, 2012). Clumped isotope values that exhibit kinetic effects from degassing result in decreased ${\text{Δ}}_{47}$ values and increased ${\text{Δ}}_{48}$ values, with an approximately linear early departure from equilibrium that has a slope of ∼−0.793 (Bajnai et al., 2020; Guo, 2020). The samples from Devils Hole do not follow this trend, as was concluded in Bajnai et al. (2021) and here (red arrow in Figure 7). Although we cannot preclude the possibility there are small, yet resolvable differences in Devils Hole clumped isotope values from samples of different ages given that these studies did not measure the same samples, the evidence here does not provide sufficient support for such a conclusion. It is noteworthy that the average ${\text{Δ}}_{47}$ and ${\text{Δ}}_{48}$ values from each study are within error of the interlaboratory ${\text{Δ}}_{47}$–${\text{Δ}}_{48}$ equilibrium regression presented in Equation 17 (Figure 7).

The combined average ${\text{Δ}}_{47}$ value from all replicates from samples in this study, Bajnai et al. (2021), and Fiebig et al. (2021), yielded a ${\text{Δ}}_{47}$ value of 0.571 ± 0.001 ‰, which yields a temperature value of 33.9 ± 0.3°C from Equation 20. The combined average ${\text{Δ}}_{48}$ value of 0.238 ± 0.007 ‰ yields a temperature value of 30.8 ± 6.8°C when input into Equation 21. Both the ${\text{Δ}}_{47}$ and ${\text{Δ}}_{48}$ reconstructed temperatures are consistent with measured temperature values from Devils Hole ranging from 32.8 to 34.3°C (Dudley & Larson, 1976; Hoffman, 1988; Miller, 1948; Plummer et al., 2000). This further supports the long-term temperature stability of Devils Hole and indicates that the equilibrium clumped isotope relationships reported here are robust.

## 5. Conclusions

This study contributes to establishing ${\text{Δ}}_{48}$ standard values that can be used in carbonate standard-based data normalization; however, further analyses of carbonate standard ${\text{Δ}}_{48}$ values may increase interlaboratory agreement. Our data supports previous research (Bernasconi et al., 2018, 2021; Dennis et al., 2011; Upadhyay et al., 2021) that carbonate-based data normalization is a robust technique for ${\text{Δ}}_{47}$, and demonstrates that it also produces statistically indistinguishable ${\text{Δ}}_{48}$ data on varying instrumentation. Carbonate-based standardization allows workers to use routinely analyzed standards for both ${\text{Δ}}_{47}$ and ${\text{Δ}}_{48}$ analyses and applies similar correction schemes to raw ${\text{Δ}}_{47}$ and ${\text{Δ}}_{48}$ values, reducing standardization error.

We have further constrained the ${\text{Δ}}_{47}$–${\text{Δ}}_{48}$, ${\text{Δ}}_{47}$-*T*, and ${\text{Δ}}_{48}$-*T* equilibrium relationships with experimental values for standards and samples, and theoretical equilibrium values, and formed regressions using data from this study and previously published work. These regressions are useful for determining if unknown samples precipitated at isotopic equilibrium and can therefore be used in accurate temperature reconstructions, or potentially to recover primary temperatures by using ${\text{Δ}}_{47}$–${\text{Δ}}_{48}$ slopes determined for various kinetic and mixing processes.

Additionally, we compared ${\text{Δ}}_{47}$ and ${\text{Δ}}_{48}$ values for Devils Hole calcite from this study and previously published work. We determined AFFs for use in transferring measured ${\text{Δ}}_{47}$ and ${\text{Δ}}_{48}$ values to ${\text{Δ}}_{63}$ and ${\text{Δ}}_{64}$ values. These AFFs are regression-form and account for the dependence of AFFs on mineral clumped isotope values. These were determined to increase accuracy when comparing measured and theoretical values.

## Supplementary Material

readme

inster-instrumental comparisons

custom R functions

analyses

user manual

Supporting Information Tables S1 - S27

Text S1 to S3; Figures S1 to S6

## Figures and Tables

**Table 1:** Description of the Mineralogy and Origin for Samples and Standards Analyzed in This Study (Bernasconi et al., 2018; Upadhyay et al., 2021; Chang et al., 2020), Including 4 Samples of Devils Hole Calcite

Standard	Mineralogy	Origin

102-GC-AZ01	Calcite	Vein carbonate from Grand Canyon, Arizona
Carmel Chalk	Calcite	Chalk
Carrara Marble, Carrara Marble CIT	Calcite	Collected in Carrara, Tuscany, Italy.
CM Tile	Calcite	Homogenized version of Carrara Marble (UCLA)
47407 Coral	Aragonite	Deep sea coral, *Desmophyllum*
DH-2-10	Calcite	Devils Hole - U.S. Geological Survey, Ash Meadows, Nevada. Core DH-2. 172 ± 4 ka
DH-2-11	Calcite	Devils Hole - U.S. Geological Survey, Ash Meadows, Nevada. Core DH-2. 163 ± 5 ka
DH-2-12	Calcite	Devils Hole - U.S. Geological Survey, Ash Meadows, Nevada. Core DH-2. 157 ± 5 ka
DH-2-13	Calcite	Devils Hole - U.S. Geological Survey, Ash Meadows, Nevada. Core DH-2. 151 ± 4 ka
ETH-1	Calcite	Carrara Marble, heated to 600°C at 155 MPa for 10 hr, sent from ETH Zurich
ETH-2	Calcite	Reagent grade synthetic, subjected to same treatment as ETH-1, sent from ETH Zurich
ETH-3	Calcite	Upper Cretaceous chalk (mostly coccoliths), Isle of Rügen, Germany, sent from ETH Zurich
ETH-4	Calcite	Same reagent grade synthetic as ETH-2, but unheated, sent from ETH Zurich
IAEA-C1	Calcite	Carrara Marble, from International Atomic Energy Agency
IAEA-C2	Travertine	Collected in Bavaria. From International Atomic Energy Agency
ISTB-1	Calcite	Speleothem from Yichang, Hubei province, China
Mallinckrodt	Calcite	Synthetic, from Mallinckrodt Baker, Inc.
MERCK	Calcite	Synthetic, from International Atomic Energy Agency
NBS 19	Calcitic Marble	Carrara Marble, from National Bureau of Standards
Spel 2-8-E	Calcite	Speleothem
SRM 88B	Dolomitic Limestone	Collected from mine site near Skokie, Illinois, USA
TB-1	Marble	Marble rock of marine origin from Quyang, Hebei province, China
TB-2	Calcite	Hydrothermal calcite from Yanji, Jilin province, China
TV01	Calcite	Travertine tile
TV03	Calcite	Travertine tile
Veinstrom	Calcite	Shallow carbonate vein collected from Tempiute Mountain, Nevada

*Note*. Uranium-series ages for Devils Hole calcite were determined by Winograd et al. (2006).

**Table 2:** Description of Mass Spectrometer Configurations Used in This Study

Configuration	Mass spectrometer model	Acid digestion temperature	Acid digestion system, sample size	*m/z* 44 ion beam intensity	Integration time	Method of data normalization

Nu Perspective-EG	Nu Instruments Perspective	90°C	Common acid bath, 5 mg	24 V before 6/2017, 18 V after 6/2017	1,600 s	25 and 1,000°C equilibrated gases
Nu Perspective-1	Nu Instruments Perspective	90°C	Common acid bath, 5 mg	24 V before 6/2017, 18 V after 6/2017	1,600 s	carbonate standard based
Nu Perspective-1a	Nu Instruments Perspective	90°C	Common acid bath, 0.5 mg	24-9 V	1,200 s	carbonate standard based
Nu Perspective-2	Nu Instruments Perspective	70°C	Nu Carb, 0.5 mg	24-9 V	1,200 s	carbonate standard based
MAT 253	Thermo Finnigan MAT 253	90°C	Common acid bath, 5 mg	16 V	720 s	carbonate standard based

**Table 3:** The ${\text{Δ}}_{47}$ and ${\text{Δ}}_{48}$ Values for Samples and Standards From This Study, Bernasconi et al. (2021), Fiebig et al. (2019), Bajnai et al. (2020), and Swart et al. (2021)

	This study, Nu Perspective-EG						Bernasconi et al. (2021)			Fiebig et al. (2019)					Fiebig et al. (2019) and Bajnai et al. (2020)					Swart et al. (2021 )				
					
Sample	N	${\text{Δ}}_{47\;\text{CDES}\;90}$ (‰)	${\text{Δ}}_{47}$ SE	N	${\text{Δ}}_{48\;\text{CDES}\;90}$ (‰)	${\text{Δ}}_{48}$ SE	N	${\text{Δ}}_{47\;\text{CDES}\;90}$ (‰)	${\text{Δ}}_{47}$ SE	N	${\text{Δ}}_{47\;\text{CDES}\;90}$ (‰)	${\text{Δ}}_{47}$ SE	${\text{Δ}}_{48\;\text{CDES}\;90}$ (‰)	${\text{Δ}}_{48}$ SE	N	${\text{Δ}}_{47\;\text{CDES}\;90}$ (‰)	${\text{Δ}}_{47}$ *SE*	${\text{Δ}}_{48\;\text{CDES}\;90}$ (‰)	${\text{Δ}}_{48}$ SE	N	${\text{Δ}}_{47\;\text{CDES}\;90}$ (‰)	${\text{Δ}}_{47}$ SE	${\text{Δ}}_{47\;\text{CDES}\;90}$ (‰)	${\text{Δ}}_{48}$SE

Carrara Marble	62	0.318	0.004	64	0.160	0.010				12	0.314	0.003	0.140	0.012										
ETH-1	36	0.205	0.004	44	0.133	0.011	232	0.205	0.002						78	0.212	0.001	0.142	0.004	19	0.214	0.006	0.145	0.012
ETH-2	30	0.200	0.004	36	0.130	0.013	215	0.209	0.002						71	0.212	0.002	0.138	0.004	14	0.203	0.004	0.153	0.011
ETH-3	35	0.617	0.003	45	0.261	0.009	264	0.613	0.001						74	0.615	0.001	0.299	0.005	20	0.629	0.005	0.269	0.007
ETH-4	36	0.462	0.004	45	0.201	0.014	162	0.451	0.002	11	0.457	0.003	0.223	0.010						14	0.459	0.005	0.231	0.010
TV03	55	0.638	0.005	55	0.269	0.007																		
Veinstrom	69	0.643	0.004	74	0.263	0.010																		

*Note*. All data in this table was normalized using only 25 and 1,000*°*C equilibrated gases.

**Table 4:** The Combined Average ${\text{Δ}}_{47}$ and ${\text{Δ}}_{48}$ Values for All Samples and Standards Analyzed in This Study

	Combined average (Nu Perspective-1, Nu Perspective-1a, Nu Perspective-2, MAT 253)				Combined average (Nu Perspective-1, Nu Perspective-1a, Nu Perspective-2)			
		
Standard	${\text{Δ}}_{47\;\text{I}-\text{CDES}}$(‰)	*N*	${\text{Δ}}_{47}$SD	${\text{Δ}}_{47}$SE	${\text{Δ}}_{48\;\text{CDES}\;90}$(‰)	*N*	${\text{Δ}}_{48}$SD	${\text{Δ}}_{48}$SE

102-GC-AZ01	0.598	24	0.028	0.006	0.240	24	0.057	0.012
Carmel Chalk	0.592	624	0.025	0.001	0.237	319	0.056	0.003
Carrara Marble	0.314	280	0.030	0.002	0.151	135	0.079	0.006
Carrara Marble CIT	0.326	21	0.027	0.006	0.144	24	0.081	0.017
CMTile	0.313	463	0.026	0.001	0.145	309	0.059	0.003
47407 Coral	0.707	9	0.025	0.008	0.275	11	0.071	0.021
DH-2-10	0.554	11	0.013	0.004	0.236	16	0.082	0.020
DH-2-11	0.560	19	0.027	0.006	0.196	17	0.035	0.009
DH-2-12	0.564	18	0.025	0.006	0.243	16	0.032	0.008
DH-2-13	0.568	17	0.027	0.006	0.261	19	0.063	0.014
DH-2 Combined	0.562	65	0.024	0.003	0.234	68	0.058	0.007
ETH-1	0.206	771	0.023	0.001	0.132	464	0.062	0.003
ETH-2	0.206	726	0.025	0.001	0.132	439	0.058	0.003
ETH-3	0.609	463	0.025	0.001	0.247	236	0.057	0.004
ETH-4	0.445	463	0.023	0.001	0.201	257	0.058	0.004
IAEA-C1	0.299	83	0.024	0.003	0.143	49	0.056	0.008
IAEA-C2	0.638	74	0.025	0.003	0.273	59	0.062	0.008
ISTB-1	0.663	15	0.059	0.015	0.297	12	0.047	0.014
Mallinckrodt	0.465	16	0.042	0.011				
Merck	0.514	81	0.030	0.003	0.234	59	0.055	0.007
NBS 19	0.316	8	0.025	0.009				
SPEL-2-8-E	0.596	11	0.035	0.011	0.245	11	0.089	0.027
SRM88B	0.528	11	0.017	0.005				
TB-1	0.327	21	0.034	0.007	0.133	23	0.089	0.019
TB-2	0.335	19	0.035	0.008	0.164	19	0.095	0.022
TV01	0.619	22	0.028	0.006	0.260	25	0.077	0.015
TV03	0.626	127	0.019	0.002	0.267	58	0.043	0.006
Veinstrom	0.633	728	0.026	0.001	0.273	436	0.061	0.003

**Table 5:** The ${\text{Δ}}_{47}$ and ${\text{Δ}}_{48}$ Values for Devils Hole Cave Calcite From This Study, Bajnai et al. (2021), and Fiebig et al. (2021)

	Sample	Age (ka)	*N*	${\text{Δ}}_{47\;\text{l}-\text{CDES}}$ (‰)	${\text{Δ}}_{47\;\text{CDES}\;90}$ (‰)	${\text{Δ}}_{47}$ SE	*N*	${\text{Δ}}_{48\;\text{CDES}\;90}$ (‰)	${\text{Δ}}_{48}$ SE

This study	DH-2-10	168-176	11	0.554		0.004	16	0.236	0.020
	DH-2-11	159-167	19	0.560		0.006	17	0.196	0.009
	DH-2-12	152-162	18	0.564		0.006	16	0.243	0.008
	DH-2-13	146-156	17	0.568		0.006	19	0.261	0.014
	Average		65	0.562		0.003	68	0.234	0.007
Bajnai et al. (2021)	DHC2-8	4.5-16.9	14		0.573	0.002	9	0.255	0.009
	DHC2-3	32.2-39.8	9		0.575	0.003	*N* is the same as for ${\text{Δ}}_{47}$	0.252	0.009
	DH-11 19.7	86.4-94.3	9		0.572	0.001		0.255	0.009
	DH-11 44.5	121.8-123.7	12		0.581	0.002		0.226	0.008
	DH-11 73.0	176.1–184.8	9		0.575	0.002		0.250	0.008
	DH-11 109.4	232.8–240.5	23		0.575	0.001		0.227	0.006
	DH-11 141.6	291.3–299.0	9		0.570	0.002		0.223	0.009
	DH-11 189.9	353.0–358.3	14		0.574	0.002		0.232	0.006
	DH-11 201.3	371.7–388.4	9		0.568	0.002		0.250	0.010
	DH-11 296.6	485.5–507.8	8		0.575	0.002		0.243	0.008
	Average		116		0.574	0.003	111	0.239	0.003
Fiebig et al. (2021)	DVH-2	4.5–16.9	9	0.582		0.003	*N* is the same as for ${\text{Δ}}_{47}$	0.246	0.012
	DHC2–8	4.5–16.9	8	0.585		0.006		0.234	0.013
	DHC2–8	4.5–16.9	9	0.572		0.003		0.234	0.012
	DHC2–8	4.5–16.9	5	0.576		0.004		0.247	0.016
	Average		31	0.580		0.002	31	0.237	0.008
Combined average	Average		212	0.571		0.001	210	0.238	0.007

*Note*. Please note that ${\text{Δ}}_{48}$ values in this table from Bajnai et al. (2021) were taken from their Supporting Information data, which provided values determined using carbonate standard based data normalization.

## Data Availability

All code used in analyses are available for review at https://doi.org/10.5281/zenodo.7311624. All replicate data are available in Supporting Information S2.

## Key Points:

- Reproducible Δ_47_ and Δ_48_ values were determined for 27 samples and standards
- Equilibrium dual clumped isotope relationships and compositionally dependent acid fractionation factors were determined
