# Supplementary material for: Equilibrated Gas and Carbonate Standard-Derived Dual (Δ47 and Δ48) Clumped Isotope Values
Source: Geochem Geophys Geosyst. Author manuscript; Available in PMC 2023 Oct 12. (PMC10569407; doi:10.1029/2022gc010458)
Supplement: readme [file NIHMS1842612-supplement-readme.rtf]

# Lucarelli-et-alCode to accompany Equilibrated gas and carbonate standard-derived dual (Δ7 and Δ8) clumped isotope valuesThe R script findcutpoints.R contains the functions that should be loaded into memory ahead of data quality control.Analyses_Lucarelli_et_al.R contains script for each data cleaning step and instructions for exporting final data to spreadsheets.Inter-instrumental_comparisons_Lucarelli_et_al.R contains inter-instrumental comparisons and power analyses.
