# Supplementary material for: Equilibrated Gas and Carbonate Standard-Derived Dual (Δ47 and Δ48) Clumped Isotope Values
Source: Geochem Geophys Geosyst. Author manuscript; Available in PMC 2023 Oct 12. (PMC10569407; doi:10.1029/2022gc010458)
Supplement: inster-instrumental comparisons [file NIHMS1842612-supplement-inster-instrumental_comparisons.rtf]

# Inter-instrumental comparisonslibrary(tidyverse)library(readxl)library(nlme)library(emmeans)library(pwr)library(rstatix)library(pracma) # Note - the pracma implementation of standard error seems to be less finicky than the base function# D48sheets <- excel_sheets("Final D48 Data Config 2 All Standards.xlsx")list_all <- lapply(sheets, function(x) read_excel("Final D48 Data Config 2 All Standards.xlsx", sheet = x))str(list_all)finalconfig2dat <- do.call("rbind", list_all)str(finalconfig2dat)sheets2 <- excel_sheets("Final D48 Data Config 3 All Standards.xlsx")list_all2 <- lapply(sheets2, function(x) read_excel("Final D48 Data Config 3 All Standards.xlsx", sheet = x))finalConfig3dat <- do.call("rbind", list_all2)sheets3 <- excel_sheets("Final Data Config 1a D48.xlsx")list_all3 <- lapply(sheets3, function(x) read_excel("Final Data Config 1a D48.xlsx", sheet = x))finalConfig1adat <- do.call("rbind", list_all3)allD48dat <- merge(finalConfig2dat, finalConfig3dat, all = TRUE)allD48dat <- merge(allD48dat, finalConfig1adat, all = TRUE)# D48 comparisonsallD48dat <- allD48dat %>%  group_by(Mass_Spec, Standard) %>%  mutate(D48CDESse = std.error(D48CDES_Final),         D47CDESse = std.error(D47CDES_Final))# Set up lmelme_D48 <- lme(D48CDES_Final ~ Standard + Mass_Spec,                data=allD48dat, random = ~1|D48CDESse, method = "REML")summary(lme_D48)# Get pairwise contrastsmodpairwise <- emmeans(lme_D48, pairwise ~ Mass_Spec)# All D47 - easier to pull from a truncated file of the supplementary tablessheets <- excel_sheets("Supplementary Tables.xlsx")list_all <- lapply(sheets, function(x) read_excel("Supplementary Tables.xlsx", sheet = x))str(list_all)finaldatall <- do.call(dplyr::bind_rows, list_all)str(finaldatall)names(finaldatall)finaldatall <- finaldatall %>%rename(  D48.CDES.90  = `Δ8 CDES 90 (‰)`,  D47.ICDES = `Δ7I-CDES (‰)`,  D47.CDES.90 = `Δ7CDES 90 (‰)`,  D47.CDES.70 = `Δ7CDES 70 (‰)`,  MassSpec = `Mass Spectrometer`,  SampleName = `Sample Name`)finaldatall <- finaldatall %>%  mutate(Standard = case_when(    !grepl("ETH", SampleName) ~ SampleName,    grepl("ETH-1", SampleName) ~ "ETH-1",    grepl("ETH-2", SampleName) ~ "ETH-2",     grepl("ETH-3", SampleName) ~ "ETH-3",     grepl("ETH-4", SampleName) ~ "ETH-4"  )  )finaldatall <- finaldatall %>%  group_by( MassSpec, Standard, Analysis) %>%  mutate(D48CDES90se = pracma::std_err(D48.CDES.90),         D47ICDESse = pracma::std_err(D47.ICDES),         D47CDES90se = pracma::std_err(D47.CDES.90),         D47CDES70se = pracma::std_err(D47.CDES.70)  )D47ICDES <- finaldatall[finaldatall$Analysis == "D47 I-CDES",]D47CDES90 <- finaldatall[finaldatall$Analysis == "D47 CDES 90",]D48CDES90 <- finaldatall[finaldatall$Analysis == "D48 CDES 90",]unique(finaldatall$Analysis)# Set up lmelme_D47ICDES <- lme(D47.ICDES ~ MassSpec+Standard,                data=D47ICDES, random = ~1|D47ICDESse, method = "REML", na.action = na.omit)summary(lme_D47ICDES)modpairwise1 <- emmeans(lme_D47ICDES, pairwise ~ MassSpec)modpairwise2 <- emmeans(lme_D47ICDES, pairwise ~ MassSpec|Standard)plot(modpairwise1,comparisons = TRUE, adjust = "mvt",      horizontal = FALSE, colors = "darkgreen") + theme_bw() +  xlab("Estimated marginal means") + ylab("")ggsave("D47ICDES_emmeansplot.tiff", dpi = 800, compression = "lzw")write.csv(modpairwise1, file = "D47ICDES_emmeans.csv", row.names = FALSE)lme_D48CDES90 <- lme(D48.CDES.90 ~ MassSpec+Standard,                     data=D48CDES90, random = ~1|D48CDES90se, method = "REML", na.action = na.omit)summary(lme_D48CDES90)modpairwise3 <- emmeans(lme_D48CDES90, pairwise ~ MassSpec)modpairwise4 <- emmeans(lme_D48CDES90, pairwise ~ Standard+MassSpec)write.csv(modpairwise4, file = "D48CDES90_emmeans_bystandard.csv", row.names = FALSE)plot(modpairwise3 ,comparisons = TRUE, adjust = "mvt",      horizontal = FALSE, colors = "darkgreen") + theme_bw()+  xlab("Estimated marginal means") + ylab("")ggsave("D48CDES90_emmeansplot.tiff", dpi = 800, compression = "lzw", scale = 1.25)plot(modpairwise4 ,comparisons = TRUE, adjust = "mvt",      horizontal = FALSE, colors = "darkgreen") + theme_bw()+  xlab("Estimated marginal means") + ylab("")ggsave("D48CDES90_emmeansplot.tiff", dpi = 800, compression = "lzw", scale = 1.25)# lme_D47CDES90 <- lme(D47.CDES.90 ~ Standard,                      data=D47CDES90, random = ~1|D47CDES90se, method = "REML", na.action = na.omit)summary(lme_D48CDES90)modpairwise5 <- emmeans(lme_D48CDES90, pairwise ~ Standard)# Power analysis# ETH-1pwr.t.test(d = (mean(D48CDES90$D48.CDES.90[D48CDES90$Standard == "ETH-1"& D48CDES90$MassSpec == "Configuration 1a"])-sd(D48CDES90$D48.CDES.90[D48CDES90$Standard == "ETH-1"& D48CDES90$MassSpec == "Configuration 1a"]))/sd(D48CDES90$D48.CDES.90[D48CDES90$Standard == "ETH-1"& D48CDES90$MassSpec == "Configuration 1a"]), sig.level = 0.05, power = 0.95, type = "two.sample")# n = 40, length = 44# ETH-2pwr.t.test(d = (mean(D48CDES90$D48.CDES.90[D48CDES90$Standard == "ETH-2"& D48CDES90$MassSpec == "Configuration 1a"])-sd(D48CDES90$D48.CDES.90[D48CDES90$Standard == "ETH-2"& D48CDES90$MassSpec == "Configuration 1a"]))/sd(D48CDES90$D48.CDES.90[D48CDES90$Standard == "ETH-2"& D48CDES90$MassSpec == "Configuration 1a"]),            sig.level = 0.05, power = 0.95, type = "two.sample")# n = 116, length = 38# ETH-3pwr.t.test(d = (mean(D48CDES90$D48.CDES.90[D48CDES90$Standard == "ETH-3"& D48CDES90$MassSpec == "Configuration 1a"])-sd(D48CDES90$D48.CDES.90[D48CDES90$Standard == "ETH-3"& D48CDES90$MassSpec == "Configuration 1a"]))/sd(D48CDES90$D48.CDES.90[D48CDES90$Standard == "ETH-3"& D48CDES90$MassSpec == "Configuration 1a"]),            sig.level = 0.05, power = 0.95, type = "two.sample")# n = 4, length = 45# ETH-4pwr.t.test(d = (mean(D48CDES90$D48.CDES.90[D48CDES90$Standard == "ETH-4"& D48CDES90$MassSpec == "Configuration 1a"])-sd(D48CDES90$D48.CDES.90[D48CDES90$Standard == "ETH-4"& D48CDES90$MassSpec == "Configuration 1a"]))/sd(D48CDES90$D48.CDES.90[D48CDES90$Standard == "ETH-4"& D48CDES90$MassSpec == "Configuration 1a"]),            sig.level = 0.05, power = 0.95, type = "two.sample")# n = 21, length = 45# TV03pwr.t.test(d = (mean(D48CDES90$D48.CDES.90[D48CDES90$Standard == "TV03"& D48CDES90$MassSpec == "Configuration 1a"])-sd(D48CDES90$D48.CDES.90[D48CDES90$Standard == "TV03"& D48CDES90$MassSpec == "Configuration 1a"]))/sd(D48CDES90$D48.CDES.90[D48CDES90$Standard == "TV03"& D48CDES90$MassSpec == "Configuration 1a"]),            sig.level = 0.05, power = 0.95, type = "two.sample")# n = 4, length = 55# Veinstrompwr.t.test(d = (mean(D48CDES90$D48.CDES.90[D48CDES90$Standard == "Veinstrom"& D48CDES90$MassSpec == "Configuration 1a"])-sd(D48CDES90$D48.CDES.90[D48CDES90$Standard == "Veinstrom"& D48CDES90$MassSpec == "Configuration 1a"]))/sd(D48CDES90$D48.CDES.90[D48CDES90$Standard == "Veinstrom"& D48CDES90$MassSpec == "Configuration 1a"]),            sig.level = 0.05, power = 0.95, type = "two.sample")# n = 7, length = 74# Carmel Chalkpwr.t.test(d = (mean(D48CDES90$D48.CDES.90[D48CDES90$Standard == "Carmel Chalk"& D48CDES90$MassSpec == "Configuration 1a"])-sd(D48CDES90$D48.CDES.90[D48CDES90$Standard == "Carmel Chalk"& D48CDES90$MassSpec == "Configuration 1a"]))/sd(D48CDES90$D48.CDES.90[D48CDES90$Standard == "Carmel Chalk"& D48CDES90$MassSpec == "Configuration 1a"]),            sig.level = 0.05, power = 0.95, type = "two.sample")# n = 4, length = 71# Carrara Marblepwr.t.test(d = (mean(D48CDES90$D48.CDES.90[D48CDES90$Standard == "Carrara Marble"& D48CDES90$MassSpec == "Configuration 1a"])-sd(D48CDES90$D48.CDES.90[D48CDES90$Standard == "Carrara Marble"& D48CDES90$MassSpec == "Configuration 1a"]))/sd(D48CDES90$D48.CDES.90[D48CDES90$Standard == "Carrara Marble"& D48CDES90$MassSpec == "Configuration 1a"]),            sig.level = 0.05, power = 0.95, type = "two.sample")# n = 29, length = 64# Config 1b and 2# ETH-1pwr.t.test(d = (mean(D48CDES90$D48.CDES.90[D48CDES90$Standard == "ETH-1"& D48CDES90$MassSpec != "Configuration 1a"])-sd(D48CDES90$D48.CDES.90[D48CDES90$Standard == "ETH-1"& D48CDES90$MassSpec != "Configuration 1a"]))/sd(D48CDES90$D48.CDES.90[D48CDES90$Standard == "ETH-1"& D48CDES90$MassSpec != "Configuration 1a"]), sig.level = 0.05, power = 0.95, type = "two.sample")# n = 11, length = 652# ETH-2pwr.t.test(d = (mean(D48CDES90$D48.CDES.90[D48CDES90$Standard == "ETH-2"& D48CDES90$MassSpec != "Configuration 1a"])-sd(D48CDES90$D48.CDES.90[D48CDES90$Standard == "ETH-2"& D48CDES90$MassSpec != "Configuration 1a"]))/sd(D48CDES90$D48.CDES.90[D48CDES90$Standard == "ETH-2"& D48CDES90$MassSpec != "Configuration 1a"]),            sig.level = 0.05, power = 0.95, type = "two.sample")# n = 17, length = 643# ETH-3pwr.t.test(d = (mean(D48CDES90$D48.CDES.90[D48CDES90$Standard == "ETH-3"& D48CDES90$MassSpec != "Configuration 1a"])-sd(D48CDES90$D48.CDES.90[D48CDES90$Standard == "ETH-3"& D48CDES90$MassSpec != "Configuration 1a"]))/sd(D48CDES90$D48.CDES.90[D48CDES90$Standard == "ETH-3"& D48CDES90$MassSpec != "Configuration 1a"]),            sig.level = 0.05, power = 0.95, type = "two.sample")# n = 4, length = 381# ETH-4pwr.t.test(d = (mean(D48CDES90$D48.CDES.90[D48CDES90$Standard == "ETH-4"& D48CDES90$MassSpec != "Configuration 1a"])-sd(D48CDES90$D48.CDES.90[D48CDES90$Standard == "ETH-4"& D48CDES90$MassSpec != "Configuration 1a"]))/sd(D48CDES90$D48.CDES.90[D48CDES90$Standard == "ETH-4"& D48CDES90$MassSpec != "Configuration 1a"]),            sig.level = 0.05, power = 0.95, type = "two.sample")# n = 6, length = 428# TV03pwr.t.test(d = (mean(D48CDES90$D48.CDES.90[D48CDES90$Standard == "TV03"& D48CDES90$MassSpec != "Configuration 1a"])-sd(D48CDES90$D48.CDES.90[D48CDES90$Standard == "TV03"& D48CDES90$MassSpec != "Configuration 1a"]))/sd(D48CDES90$D48.CDES.90[D48CDES90$Standard == "TV03"& D48CDES90$MassSpec != "Configuration 1a"]),            sig.level = 0.05, power = 0.95, type = "two.sample")# n = 3, length = 90# Veinstrompwr.t.test(d = (mean(D48CDES90$D48.CDES.90[D48CDES90$Standard == "Veinstrom"& D48CDES90$MassSpec != "Configuration 1a"])-sd(D48CDES90$D48.CDES.90[D48CDES90$Standard == "Veinstrom"& D48CDES90$MassSpec != "Configuration 1a"]))/sd(D48CDES90$D48.CDES.90[D48CDES90$Standard == "Veinstrom"& D48CDES90$MassSpec != "Configuration 1a"]),            sig.level = 0.05, power = 0.95, type = "two.sample")# n = 4, length = 629# Carmel Chalkpwr.t.test(d = (mean(D48CDES90$D48.CDES.90[D48CDES90$Standard == "Carmel Chalk"& D48CDES90$MassSpec != "Configuration 1a"])-sd(D48CDES90$D48.CDES.90[D48CDES90$Standard == "Carmel Chalk"& D48CDES90$MassSpec != "Configuration 1a"]))/sd(D48CDES90$D48.CDES.90[D48CDES90$Standard == "Carmel Chalk"& D48CDES90$MassSpec != "Configuration 1a"]),            sig.level = 0.05, power = 0.95, type = "two.sample")# n = 4, length = 485# Carrara Marblepwr.t.test(d = (mean(D48CDES90$D48.CDES.90[D48CDES90$Standard == "Carrara Marble"& D48CDES90$MassSpec != "Configuration 1a"])-sd(D48CDES90$D48.CDES.90[D48CDES90$Standard == "Carrara Marble"& D48CDES90$MassSpec != "Configuration 1a"]))/sd(D48CDES90$D48.CDES.90[D48CDES90$Standard == "Carrara Marble"& D48CDES90$MassSpec != "Configuration 1a"]),            sig.level = 0.05, power = 0.95, type = "two.sample")# n = 20, length 215# CMTilepwr.t.test(d = (mean(D48CDES90$D48.CDES.90[D48CDES90$Standard == "CMTile"& D48CDES90$MassSpec != "Configuration 1a"])-sd(D48CDES90$D48.CDES.90[D48CDES90$Standard == "CMTile"& D48CDES90$MassSpec != "Configuration 1a"]))/sd(D48CDES90$D48.CDES.90[D48CDES90$Standard == "CMTile"& D48CDES90$MassSpec != "Configuration 1a"]),            sig.level = 0.05, power = 0.95, type = "two.sample")# n = 13, length = 453# MERCKpwr.t.test(d = (mean(D48CDES90$D48.CDES.90[D48CDES90$Standard == "MERCK" & D48CDES90$MassSpec != "Configuration 1a"])-sd(D48CDES90$D48.CDES.90[D48CDES90$Standard == "MERCK"& D48CDES90$MassSpec != "Configuration 1a"]))/sd(D48CDES90$D48.CDES.90[D48CDES90$Standard == "MERCK"& D48CDES90$MassSpec != "Configuration 1a"]),            sig.level = 0.05, power = 0.95, type = "two.sample")# n = 4, length = 70# Configuration 3# ETH-1pwr.t.test(d = (mean(D48CDES90$D48.CDES.90[D48CDES90$Standard == "ETH-1"& D48CDES90$MassSpec == "Configuration 3"])-sd(D48CDES90$D48.CDES.90[D48CDES90$Standard == "ETH-1"& D48CDES90$MassSpec == "Configuration 3"]))/sd(D48CDES90$D48.CDES.90[D48CDES90$Standard == "ETH-1"& D48CDES90$MassSpec == "Configuration 3"]), sig.level = 0.05, power = 0.95, type = "two.sample")# n = 250, length 188# ETH-2pwr.t.test(d = (mean(D48CDES90$D48.CDES.90[D48CDES90$Standard == "ETH-2"& D48CDES90$MassSpec == "Configuration 3"])-sd(D48CDES90$D48.CDES.90[D48CDES90$Standard == "ETH-2"& D48CDES90$MassSpec == "Configuration 3"]))/sd(D48CDES90$D48.CDES.90[D48CDES90$Standard == "ETH-2"& D48CDES90$MassSpec == "Configuration 3"]),            sig.level = 0.05, power = 0.95, type = "two.sample")# n = 116, length = 204# ETH-3pwr.t.test(d = (mean(D48CDES90$D48.CDES.90[D48CDES90$Standard == "ETH-3"& D48CDES90$MassSpec == "Configuration 3"])-sd(D48CDES90$D48.CDES.90[D48CDES90$Standard == "ETH-3"& D48CDES90$MassSpec == "Configuration 3"]))/sd(D48CDES90$D48.CDES.90[D48CDES90$Standard == "ETH-3"& D48CDES90$MassSpec == "Configuration 3"]),            sig.level = 0.05, power = 0.95, type = "two.sample")# n = 8, length = 145# ETH-4pwr.t.test(d = (mean(D48CDES90$D48.CDES.90[D48CDES90$Standard == "ETH-4"& D48CDES90$MassSpec == "Configuration 3"])-sd(D48CDES90$D48.CDES.90[D48CDES90$Standard == "ETH-4"& D48CDES90$MassSpec == "Configuration 3"]))/sd(D48CDES90$D48.CDES.90[D48CDES90$Standard == "ETH-4"& D48CDES90$MassSpec == "Configuration 3"]),            sig.level = 0.05, power = 0.95, type = "two.sample")# n = 31, length = 171# TV03pwr.t.test(d = (mean(D48CDES90$D48.CDES.90[D48CDES90$Standard == "TV03"& D48CDES90$MassSpec == "Configuration 3"])-sd(D48CDES90$D48.CDES.90[D48CDES90$Standard == "TV03"& D48CDES90$MassSpec == "Configuration 3"]))/sd(D48CDES90$D48.CDES.90[D48CDES90$Standard == "TV03"& D48CDES90$MassSpec == "Configuration 3"]),            sig.level = 0.05, power = 0.95, type = "two.sample")# n = 6, length = 32# Veinstrompwr.t.test(d = (mean(D48CDES90$D48.CDES.90[D48CDES90$Standard == "Veinstrom"& D48CDES90$MassSpec == "Configuration 3"])-sd(D48CDES90$D48.CDES.90[D48CDES90$Standard == "Veinstrom"& D48CDES90$MassSpec == "Configuration 3"]))/sd(D48CDES90$D48.CDES.90[D48CDES90$Standard == "Veinstrom"& D48CDES90$MassSpec == "Configuration 3"]),            sig.level = 0.05, power = 0.95, type = "two.sample")# n = 7, length = 193# Carmel Chalkpwr.t.test(d = (mean(D48CDES90$D48.CDES.90[D48CDES90$Standard == "Carmel Chalk"& D48CDES90$MassSpec == "Configuration 3"])-sd(D48CDES90$D48.CDES.90[D48CDES90$Standard == "Carmel Chalk"& D48CDES90$MassSpec == "Configuration 3"]))/sd(D48CDES90$D48.CDES.90[D48CDES90$Standard == "Carmel Chalk"& D48CDES90$MassSpec == "Configuration 3"]),            sig.level = 0.05, power = 0.95, type = "two.sample")# n = 9, length = 166# Carrara Marblepwr.t.test(d = (mean(D48CDES90$D48.CDES.90[D48CDES90$Standard == "Carrara Marble"& D48CDES90$MassSpec == "Configuration 3"])-sd(D48CDES90$D48.CDES.90[D48CDES90$Standard == "Carrara Marble"& D48CDES90$MassSpec == "Configuration 3"]))/sd(D48CDES90$D48.CDES.90[D48CDES90$Standard == "Carrara Marble"& D48CDES90$MassSpec == "Configuration 3"]),            sig.level = 0.05, power = 0.95, type = "two.sample")# n = 3116, length = 80# CMTilepwr.t.test(d = (mean(D48CDES90$D48.CDES.90[D48CDES90$Standard == "CMTile"& D48CDES90$MassSpec == "Configuration 3"])-sd(D48CDES90$D48.CDES.90[D48CDES90$Standard == "CMTile"& D48CDES90$MassSpec == "Configuration 3"]))/sd(D48CDES90$D48.CDES.90[D48CDES90$Standard == "CMTile"& D48CDES90$MassSpec == "Configuration 3"]),            sig.level = 0.05, power = 0.95, type = "two.sample")# n = 77, length = 144# MERCKpwr.t.test(d = (mean(D48CDES90$D48.CDES.90[D48CDES90$Standard == "MERCK" & D48CDES90$MassSpec == "Configuration 3"])-sd(D48CDES90$D48.CDES.90[D48CDES90$Standard == "MERCK"& D48CDES90$MassSpec == "Configuration 3"]))/sd(D48CDES90$D48.CDES.90[D48CDES90$Standard == "MERCK"& D48CDES90$MassSpec == "Configuration 3"]),            sig.level = 0.05, power = 0.95, type = "two.sample")# n = 31103.84, length = 11# Normality of all final datasetssheets <- excel_sheets("Supplementary Tables.xlsx")list_all <- lapply(sheets, function(x) read_excel("Supplementary Tables.xlsx", sheet = x))str(list_all)finaldatall <- do.call(dplyr::bind_rows, list_all)str(finaldatall)names(finaldatall)finaldatall <- finaldatall %>%  mutate(Standard = case_when(    !grepl("ETH", `Sample Name`) ~ `Sample Name`,    grepl("ETH-1", `Sample Name`) ~ "ETH-1",    grepl("ETH-2", `Sample Name`) ~ "ETH-2",     grepl("ETH-3", `Sample Name`) ~ "ETH-3",     grepl("ETH-4", `Sample Name`) ~ "ETH-4"  )  )names(finaldatall)<-make.names(names(finaldatall),unique = TRUE) # Standardize names# split by analysisD48CDES90 <- finaldatall %>% filter(Analysis == "Δ8 CDES 90")D47CDES90 <- finaldatall %>% filter(Analysis == "Δ7 CDES 90")D47ICDES <- finaldatall %>% filter(Analysis == "Δ7 I-CDES")unique(D48CDES90$Mass.Spectrometer)unique(finaldatall$Standard)# D48 CDES 90D48CDES90_summary <- D48CDES90 %>%  group_by(`Mass.Spectrometer`, Standard) %>%  summarize(`Mean Δ8 CDES 90 (‰)` = round(mean(`Δ8.CDES.90....`), 3),            `Min Δ8 CDES 90 (‰)` = min(`Δ8.CDES.90....`),            `Max Δ8 CDES 90 (‰)` = max(`Δ8.CDES.90....`),            `SD Δ8 CDES 90 (‰)` = round(sd(`Δ8.CDES.90....`), 3),            `N Δ8 CDES 90 (‰)` = length(`Δ8.CDES.90....`),                        na.action(na.omit)                        )D48CDES90_shap <- D48CDES90 %>%  group_by(`Mass.Spectrometer`, Standard) %>%  filter(n() >=3) %>%  shapiro_test(`Δ8.CDES.90....`)D48CDES90_summary_final <- merge(D48CDES90_summary, D48CDES90_shap, all = TRUE)write.csv(D48CDES90_summary_final, "D48CDES90_summary_final2.csv", row.names = FALSE)D48CDES90 <- D48CDES90 %>%  group_by(`Mass.Spectrometer`, Standard) %>%  mutate(D48CDES90se = pracma::std_err(`Δ8.CDES.90....`), na.action(na.omit))lme_D48CDES90 <- lme(`Δ8.CDES.90....` ~ Mass.Spectrometer + Standard,                     data=D48CDES90[D48CDES90$Mass.Spectrometer != "Multiple" & !is.na(D48CDES90$D48CDES90se),], random = ~1|D48CDES90se , method = "REML", na.action = na.omit)summary(lme_D48CDES90)modpairwise3 <- emmeans(lme_D48CDES90, pairwise ~ `Mass.Spectrometer`)plot(modpairwise3,comparisons = TRUE, adjust = "mvt",      horizontal = FALSE, colors = "darkgreen") + theme_bw() +  xlab("Estimated marginal means") + ylab("")ggsave("D48CDES90_emmeansplot.tiff", dpi = 800, compression = "lzw", scale = 1.25)write.csv(modpairwise3, file = "D48CDES90_emmeans.csv", row.names = FALSE)# D47 CDES 90D47CDES90_summary <- D47CDES90 %>%  group_by(`Mass.Spectrometer`, Standard) %>%  summarize(`Mean Δ7 CDES 90 (‰)` = round(mean(`Δ7CDES.90....`), 3),            `Min Δ7 CDES 90 (‰)` = min(`Δ7CDES.90....`),            `Max Δ7 CDES 90 (‰)` = max(`Δ7CDES.90....`),            `SD Δ7 CDES 90 (‰)` = round(sd(`Δ7CDES.90....`), 3),            `N Δ7 CDES 90 (‰)` = length(`Δ7CDES.90....`),            na.action(na.omit)              )D47CDES90_shap <- D47CDES90 %>%  group_by(`Mass.Spectrometer`, Standard) %>%  filter(n() >=3) %>%  shapiro_test(`Δ7CDES.90....`)# Make a nice summary tableD47CDES90_summary_final <- merge(D47CDES90_summary, D47CDES90_shap, all = TRUE)write.csv(D47CDES90_summary_final, "D47CDES90_summary_final.csv", row.names = FALSE)# D47 ICDESD47ICDES_summary <- D47ICDES %>%  group_by(`Mass.Spectrometer`, Standard) %>%  summarize(`Mean Δ7 ICDES (‰)` = round(mean(`Δ7I.CDES....`), 3),            `Min Δ7 ICDES (‰)` = min(`Δ7I.CDES....`),            `Max Δ7 ICDES (‰)` = max(`Δ7I.CDES....`),            `SD Δ7 ICDES (‰)` = round(sd(`Δ7I.CDES....`), 3),            `N Δ7 ICDES (‰)` = length(`Δ7I.CDES....`),            na.action(na.omit)              )D47ICDES_shap <- D47ICDES %>%  group_by(`Mass.Spectrometer`, Standard) %>%  filter(n() >=3) %>%  shapiro_test(`Δ7I.CDES....`)D47ICDES <- D47ICDES %>%  group_by(`Mass.Spectrometer`, Standard) %>%  mutate(D47ICDESse = pracma::std_err(`Δ7I.CDES....`), na.action(na.omit))# Make a nice summary tableD47ICDES_summary_final <- merge(D47ICDES_summary, D47ICDES_shap, all = TRUE)write.csv(D47ICDES_summary_final, "D47ICDES_summary_final2.csv", row.names = FALSE)# Shorten the namesD47ICDES$MassSpec2 <- recode(D47ICDES$`Mass.Spectrometer`, "Config 1a" = "C1a", "Config 1b" = "C1b", "Config 1c" = "C1c", "Config 1d" = "C1d", "Config 2" = "C2",                              "Config 3" = "C3") names(D47ICDES)# Set up lmelme_D47ICDES <- lme(`Δ7I.CDES....` ~ `Mass.Spectrometer`+Standard,                     data=D47ICDES[D47ICDES$Mass.Spectrometer != "Multiple" & !is.na(D47ICDES$D47ICDESse),], random = ~1|D47ICDESse , method = "REML", na.action = na.omit)summary(lme_D47ICDES)# Get pairwise contrastsmodpairwise1 <- emmeans(lme_D47ICDES, pairwise ~ `Mass.Spectrometer`)modpairwise2 <- emmeans(lme_D47ICDES, pairwise ~ `Mass.Spectrometer`|Standard)plot(modpairwise1,comparisons = TRUE, adjust = "mvt",      horizontal = FALSE, colors = "darkgreen") + theme_bw() +  xlab("Estimated marginal means") + ylab("")ggsave("D47ICDES_emmeansplot.tiff", dpi = 800, compression = "lzw", scale = 1.25)write.csv(modpairwise1, file = "D47ICDES_emmeans.csv", row.names = FALSE)# Power analysis, Configs 1b and 3, D47 ICDES# ETH-1pwr.t.test(d = (mean(D47ICDES$`Δ7I.CDES....`[D47ICDES$`Mass.Spectrometer` == "Config 1b"| D47ICDES$`Mass.Spectrometer` == "Config 3" & D47ICDES$Standard == "ETH-1"])-sd(D47ICDES$`Δ7I.CDES....`[D47ICDES$`Mass.Spectrometer` == "Config 1b"|D47ICDES$`Mass.Spectrometer` == "Config 3" & D47ICDES$Standard == "ETH-1"]))/sd(D47ICDES$`Δ7I.CDES....`[D47ICDES$`Mass.Spectrometer` == "Config 1b"|D47ICDES$`Mass.Spectrometer` == "Config 3" & D47ICDES$Standard == "ETH-1"]), sig.level = 0.05, power = 0.95, type = "two.sample")# n = 27, length = 907# ETH-2pwr.t.test(d = (mean(D47ICDES$`Δ7I.CDES....`[D47ICDES$`Mass.Spectrometer` == "Config 1b"| D47ICDES$`Mass.Spectrometer` == "Config 3" & D47ICDES$Standard == "ETH-2"])-sd(D47ICDES$`Δ7I.CDES....`[D47ICDES$`Mass.Spectrometer` == "Config 1b"|D47ICDES$`Mass.Spectrometer` == "Config 3" & D47ICDES$Standard == "ETH-2"]))/sd(D47ICDES$`Δ7I.CDES....`[D47ICDES$`Mass.Spectrometer` == "Config 1b"|D47ICDES$`Mass.Spectrometer` == "Config 3" & D47ICDES$Standard == "ETH-2"]), sig.level = 0.05, power = 0.95, type = "two.sample")# n = 26, length = 894# ETH-3pwr.t.test(d = (mean(D47ICDES$`Δ7I.CDES....`[D47ICDES$`Mass.Spectrometer` == "Config 1b"| D47ICDES$`Mass.Spectrometer` == "Config 3" & D47ICDES$Standard == "ETH-3"])-sd(D47ICDES$`Δ7I.CDES....`[D47ICDES$`Mass.Spectrometer` == "Config 1b"|D47ICDES$`Mass.Spectrometer` == "Config 3" & D47ICDES$Standard == "ETH-3"]))/sd(D47ICDES$`Δ7I.CDES....`[D47ICDES$`Mass.Spectrometer` == "Config 1b"|D47ICDES$`Mass.Spectrometer` == "Config 3" & D47ICDES$Standard == "ETH-3"]), sig.level = 0.05, power = 0.95, type = "two.sample")# n = 8, length = 833# ETH-4pwr.t.test(d = (mean(D47ICDES$`Δ7I.CDES....`[D47ICDES$`Mass.Spectrometer` == "Config 1b"| D47ICDES$`Mass.Spectrometer` == "Config 3" & D47ICDES$Standard == "ETH-4"])-sd(D47ICDES$`Δ7I.CDES....`[D47ICDES$`Mass.Spectrometer` == "Config 1b"|D47ICDES$`Mass.Spectrometer` == "Config 3" & D47ICDES$Standard == "ETH-4"]))/sd(D47ICDES$`Δ7I.CDES....`[D47ICDES$`Mass.Spectrometer` == "Config 1b"|D47ICDES$`Mass.Spectrometer` == "Config 3" & D47ICDES$Standard == "ETH-4"]), sig.level = 0.05, power = 0.95, type = "two.sample")# n = 8, length = 831# TV03pwr.t.test(d = (mean(D47ICDES$`Δ7I.CDES....`[D47ICDES$`Mass.Spectrometer` == "Config 1b"| D47ICDES$`Mass.Spectrometer` == "Config 3" & D47ICDES$Standard == "TV03"])-sd(D47ICDES$`Δ7I.CDES....`[D47ICDES$`Mass.Spectrometer` == "Config 1b"|D47ICDES$`Mass.Spectrometer` == "Config 3" & D47ICDES$Standard == "TV03"]))/sd(D47ICDES$`Δ7I.CDES....`[D47ICDES$`Mass.Spectrometer` == "Config 1b"|D47ICDES$`Mass.Spectrometer` == "Config 3" & D47ICDES$Standard == "TV03"]), sig.level = 0.05, power = 0.95, type = "two.sample")# n = 10, length = 703# Veinstrompwr.t.test(d = (mean(D47ICDES$`Δ7I.CDES....`[D47ICDES$`Mass.Spectrometer` == "Config 1b"| D47ICDES$`Mass.Spectrometer` == "Config 3" & D47ICDES$Standard == "Veinstrom"])-sd(D47ICDES$`Δ7I.CDES....`[D47ICDES$`Mass.Spectrometer` == "Config 1b"|D47ICDES$`Mass.Spectrometer` == "Config 3" & D47ICDES$Standard == "Veinstrom"]))/sd(D47ICDES$`Δ7I.CDES....`[D47ICDES$`Mass.Spectrometer` == "Config 1b"|D47ICDES$`Mass.Spectrometer` == "Config 3" & D47ICDES$Standard == "Veinstrom"]), sig.level = 0.05, power = 0.95, type = "two.sample")# n = 7, length = 927# Carmel Chalkpwr.t.test(d = (mean(D47ICDES$`Δ7I.CDES....`[D47ICDES$`Mass.Spectrometer` == "Config 1b"| D47ICDES$`Mass.Spectrometer` == "Config 3" & D47ICDES$Standard == "Carmel Chalk"])-sd(D47ICDES$`Δ7I.CDES....`[D47ICDES$`Mass.Spectrometer` == "Config 1b"|D47ICDES$`Mass.Spectrometer` == "Config 3" & D47ICDES$Standard == "Carmel Chalk"]))/sd(D47ICDES$`Δ7I.CDES....`[D47ICDES$`Mass.Spectrometer` == "Config 1b"|D47ICDES$`Mass.Spectrometer` == "Config 3" & D47ICDES$Standard == "Carmel Chalk"]), sig.level = 0.05, power = 0.95, type = "two.sample")# n = 7, length = 905# Carrara Marblepwr.t.test(d = (mean(D47ICDES$`Δ7I.CDES....`[D47ICDES$`Mass.Spectrometer` == "Config 1b"| D47ICDES$`Mass.Spectrometer` == "Config 3" & D47ICDES$Standard == "Carrara Marble"])-sd(D47ICDES$`Δ7I.CDES....`[D47ICDES$`Mass.Spectrometer` == "Config 1b"|D47ICDES$`Mass.Spectrometer` == "Config 3" & D47ICDES$Standard == "Carrara Marble"]))/sd(D47ICDES$`Δ7I.CDES....`[D47ICDES$`Mass.Spectrometer` == "Config 1b"|D47ICDES$`Mass.Spectrometer` == "Config 3" & D47ICDES$Standard == "Carrara Marble"]), sig.level = 0.05, power = 0.95, type = "two.sample")# n = 13, length = 778# Config 2, D47 ICDES# ETH-1pwr.t.test(d = (mean(D47ICDES$`Δ7I.CDES....`[D47ICDES$`Mass.Spectrometer` == "Config 2" & D47ICDES$Standard == "ETH-1"])-sd(D47ICDES$`Δ7I.CDES....`[D47ICDES$`Mass.Spectrometer` == "Config 2" & D47ICDES$Standard == "ETH-1"]))/sd(D47ICDES$`Δ7I.CDES....`[D47ICDES$`Mass.Spectrometer` == "Config 2" & D47ICDES$Standard == "ETH-1"]), sig.level = 0.05, power = 0.95, type = "two.sample")# n = 3, length = 402# ETH-2pwr.t.test(d = (mean(D47ICDES$`Δ7I.CDES....`[D47ICDES$`Mass.Spectrometer` == "Config 2" & D47ICDES$Standard == "ETH-2"])-sd(D47ICDES$`Δ7I.CDES....`[D47ICDES$`Mass.Spectrometer` == "Config 2" & D47ICDES$Standard == "ETH-2"]))/sd(D47ICDES$`Δ7I.CDES....`[D47ICDES$`Mass.Spectrometer` == "Config 2" & D47ICDES$Standard == "ETH-2"]), sig.level = 0.05, power = 0.95, type = "two.sample")# n = 3, length = 386# ETH-3pwr.t.test(d = (mean(D47ICDES$`Δ7I.CDES....`[D47ICDES$`Mass.Spectrometer` == "Config 2" & D47ICDES$Standard == "ETH-3"])-sd(D47ICDES$`Δ7I.CDES....`[D47ICDES$`Mass.Spectrometer` == "Config 2" & D47ICDES$Standard == "ETH-3"]))/sd(D47ICDES$`Δ7I.CDES....`[D47ICDES$`Mass.Spectrometer` == "Config 2" & D47ICDES$Standard == "ETH-3"]), sig.level = 0.05, power = 0.95, type = "two.sample")# Error in uniroot(function(n) eval(p.body) - power, c(2 + 1e-10, 1e+09)) : # f() values at end points not of opposite sign# ETH-4pwr.t.test(d = (mean(D47ICDES$`Δ7I.CDES....`[D47ICDES$`Mass.Spectrometer` == "Config 2" & D47ICDES$Standard == "ETH-4"])-sd(D47ICDES$`Δ7I.CDES....`[D47ICDES$`Mass.Spectrometer` == "Config 2" & D47ICDES$Standard == "ETH-4"]))/sd(D47ICDES$`Δ7I.CDES....`[D47ICDES$`Mass.Spectrometer` == "Config 2" & D47ICDES$Standard == "ETH-4"]), sig.level = 0.05, power = 0.95, type = "two.sample")# Error in uniroot(function(n) eval(p.body) - power, c(2 + 1e-10, 1e+09)) : # f() values at end points not of opposite sign# TV03pwr.t.test(d = (mean(D47ICDES$`Δ7I.CDES....`[D47ICDES$`Mass.Spectrometer` == "Config 2" & D47ICDES$Standard == "TV03"])-sd(D47ICDES$`Δ7I.CDES....`[D47ICDES$`Mass.Spectrometer` == "Config 2" & D47ICDES$Standard == "TV03"]))/sd(D47ICDES$`Δ7I.CDES....`[D47ICDES$`Mass.Spectrometer` == "Config 2" & D47ICDES$Standard == "TV03"]), sig.level = 0.05, power = 0.95, type = "two.sample")# Error in uniroot(function(n) eval(p.body) - power, c(2 + 1e-10, 1e+09)) : # f() values at end points not of opposite sign# Veinstrompwr.t.test(d = (mean(D47ICDES$`Δ7I.CDES....`[D47ICDES$`Mass.Spectrometer` == "Config 2" & D47ICDES$Standard == "Veinstrom"])-sd(D47ICDES$`Δ7I.CDES....`[D47ICDES$`Mass.Spectrometer` == "Config 2" & D47ICDES$Standard == "Veinstrom"]))/sd(D47ICDES$`Δ7I.CDES....`[D47ICDES$`Mass.Spectrometer` == "Config 2" & D47ICDES$Standard == "Veinstrom"]), sig.level = 0.05, power = 0.95, type = "two.sample")# Error in uniroot(function(n) eval(p.body) - power, c(2 + 1e-10, 1e+09)) : # f() values at end points not of opposite sign# Carmel Chalkpwr.t.test(d = (mean(D47ICDES$`Δ7I.CDES....`[D47ICDES$`Mass.Spectrometer` == "Config 2" & D47ICDES$Standard == "Carmel Chalk"])-sd(D47ICDES$`Δ7I.CDES....`[D47ICDES$`Mass.Spectrometer` == "Config 2" & D47ICDES$Standard == "Carmel Chalk"]))/sd(D47ICDES$`Δ7I.CDES....`[D47ICDES$`Mass.Spectrometer` == "Config 2" & D47ICDES$Standard == "Carmel Chalk"]), sig.level = 0.05, power = 0.95, type = "two.sample")# Error in uniroot(function(n) eval(p.body) - power, c(2 + 1e-10, 1e+09)) : # f() values at end points not of opposite sign# Carrara Marblepwr.t.test(d = (mean(D47ICDES$`Δ7I.CDES....`[D47ICDES$`Mass.Spectrometer` == "Config 2" & D47ICDES$Standard == "Carrara Marble"])-sd(D47ICDES$`Δ7I.CDES....`[D47ICDES$`Mass.Spectrometer` == "Config 2" & D47ICDES$Standard == "Carrara Marble"]))/sd(D47ICDES$`Δ7I.CDES....`[D47ICDES$`Mass.Spectrometer` == "Config 2" & D47ICDES$Standard == "Carrara Marble"]), sig.level = 0.05, power = 0.95, type = "two.sample")# n = 3, length = 44# Config 1a# ETH-1pwr.t.test(d = (mean(D47CDES90$`Δ7CDES.90....`[D47CDES90$`Mass.Spectrometer` == "Config 1a" & D47CDES90$Standard == "ETH-1"])-sd(D47CDES90$`Δ7CDES.90....`[D47CDES90$`Mass.Spectrometer` == "Config 1a" & D47CDES90$Standard == "ETH-1"]))/sd(D47CDES90$`Δ7CDES.90....`[D47CDES90$`Mass.Spectrometer` == "Config 1a" & D47CDES90$Standard == "ETH-1"]), sig.level = 0.05, power = 0.95, type = "two.sample")# n = 3, length = 36# ETH-2pwr.t.test(d = (mean(D47CDES90$`Δ7CDES.90....`[D47CDES90$`Mass.Spectrometer` == "Config 1a" & D47CDES90$Standard == "ETH-2"])-sd(D47CDES90$`Δ7CDES.90....`[D47CDES90$`Mass.Spectrometer` == "Config 1a" & D47CDES90$Standard == "ETH-2"]))/sd(D47CDES90$`Δ7CDES.90....`[D47CDES90$`Mass.Spectrometer` == "Config 1a" & D47CDES90$Standard == "ETH-2"]), sig.level = 0.05, power = 0.95, type = "two.sample")# Error in uniroot(function(n) eval(p.body) - power, c(2 + 1e-10, 1e+09)) : # f() values at end points not of opposite sign# ETH-3pwr.t.test(d = (mean(D47CDES90$`Δ7CDES.90....`[D47CDES90$`Mass.Spectrometer` == "Config 1a" & D47CDES90$Standard == "ETH-3"])-sd(D47CDES90$`Δ7CDES.90....`[D47CDES90$`Mass.Spectrometer` == "Config 1a" & D47CDES90$Standard == "ETH-3"]))/sd(D47CDES90$`Δ7CDES.90....`[D47CDES90$`Mass.Spectrometer` == "Config 1a" & D47CDES90$Standard == "ETH-3"]), sig.level = 0.05, power = 0.95, type = "two.sample")# Error in uniroot(function(n) eval(p.body) - power, c(2 + 1e-10, 1e+09)) : # f() values at end points not of opposite sign# ETH-4pwr.t.test(d = (mean(D47CDES90$`Δ7CDES.90....`[D47CDES90$`Mass.Spectrometer` == "Config 1a" & D47CDES90$Standard == "ETH-4"])-sd(D47CDES90$`Δ7CDES.90....`[D47CDES90$`Mass.Spectrometer` == "Config 1a" & D47CDES90$Standard == "ETH-4"]))/sd(D47CDES90$`Δ7CDES.90....`[D47CDES90$`Mass.Spectrometer` == "Config 1a" & D47CDES90$Standard == "ETH-4"]), sig.level = 0.05, power = 0.95, type = "two.sample")# Error in uniroot(function(n) eval(p.body) - power, c(2 + 1e-10, 1e+09)) : # f() values at end points not of opposite sign# TV03pwr.t.test(d = (mean(D47CDES90$`Δ7CDES.90....`[D47CDES90$`Mass.Spectrometer` == "Config 1a" & D47CDES90$Standard == "TV03"])-sd(D47CDES90$`Δ7CDES.90....`[D47CDES90$`Mass.Spectrometer` == "Config 1a" & D47CDES90$Standard == "TV03"]))/sd(D47CDES90$`Δ7CDES.90....`[D47CDES90$`Mass.Spectrometer` == "Config 1a" & D47CDES90$Standard == "TV03"]), sig.level = 0.05, power = 0.95, type = "two.sample")# Error in uniroot(function(n) eval(p.body) - power, c(2 + 1e-10, 1e+09)) : # f() values at end points not of opposite sign# Veinstrompwr.t.test(d = (mean(D47CDES90$`Δ7CDES.90....`[D47CDES90$`Mass.Spectrometer` == "Config 1a" & D47CDES90$Standard == "Veinstrom"])-sd(D47CDES90$`Δ7CDES.90....`[D47CDES90$`Mass.Spectrometer` == "Config 1a" & D47CDES90$Standard == "Veinstrom"]))/sd(D47CDES90$`Δ7CDES.90....`[D47CDES90$`Mass.Spectrometer` == "Config 1a" & D47CDES90$Standard == "Veinstrom"]), sig.level = 0.05, power = 0.95, type = "two.sample")# Error in uniroot(function(n) eval(p.body) - power, c(2 + 1e-10, 1e+09)) : # f() values at end points not of opposite sign# Carmel Chalkpwr.t.test(d = (mean(D47CDES90$`Δ7CDES.90....`[D47CDES90$`Mass.Spectrometer` == "Config 1a" & D47CDES90$Standard == "Carmel Chalk"])-sd(D47CDES90$`Δ7CDES.90....`[D47CDES90$`Mass.Spectrometer` == "Config 1a" & D47CDES90$Standard == "Carmel Chalk"]))/sd(D47CDES90$`Δ7CDES.90....`[D47CDES90$`Mass.Spectrometer` == "Config 1a" & D47CDES90$Standard == "Carmel Chalk"]), sig.level = 0.05, power = 0.95, type = "two.sample")# Error in uniroot(function(n) eval(p.body) - power, c(2 + 1e-10, 1e+09)) : # f() values at end points not of opposite sign# Carrara Marblepwr.t.test(d = (mean(D47CDES90$`Δ7CDES.90....`[D47CDES90$`Mass.Spectrometer` == "Config 1a" & D47CDES90$Standard == "Carrara Marble"])-sd(D47CDES90$`Δ7CDES.90....`[D47CDES90$`Mass.Spectrometer` == "Config 1a" & D47CDES90$Standard == "Carrara Marble"]))/sd(D47CDES90$`Δ7CDES.90....`[D47CDES90$`Mass.Spectrometer` == "Config 1a" & D47CDES90$Standard == "Carrara Marble"]), sig.level = 0.05, power = 0.95, type = "two.sample")# Error in uniroot(function(n) eval(p.body) - power, c(2 + 1e-10, 1e+09)) : # f() values at end points not of opposite sign
