# Supplementary material for: Equilibrated Gas and Carbonate Standard-Derived Dual (Δ47 and Δ48) Clumped Isotope Values
Source: Geochem Geophys Geosyst. Author manuscript; Available in PMC 2023 Oct 12. (PMC10569407; doi:10.1029/2022gc010458)
Supplement: custom R functions [file NIHMS1842612-supplement-custom_R_functions.rtf]

findCutpoints <- function(x){    dens <- density(x) # Kernel density estimate of supplied data    # Min/max method   minima  <- which(diff(sign(diff(c(Inf, dens$y, Inf)))) == 2)    # index of max value  maxima <- which.max(dens$y)  maximum <- dens$x[maxima]    # combine indexes of local minima and the max  minmax <- sort(c(minima, maxima))    # select the two minima on either side of the max    nearestminima <- minmax[which(minmax == maxima) + c(-1, 1)]    # Numeric cutpoints  mincut <- dens$x[nearestminima[1]]  maxcut <- dens$x[nearestminima[2]]    # Zoomed in plot showing cut points (solid lines) and maximum (dotted line)    plot(dens, xlim = c((mincut-0.5), (maxcut+0.5)), main = "First recommended exclusions")  abline(v=mincut)  abline(v=maxcut)  abline(v=maximum, lty=2)    cuts <- c(mincut, maxcut, maximum)  return(cuts)  }findCutpointsDouble <- function(x){    dens <- density(x) # Kernel density estimate of supplied data    # Min/max method   minima  <- which(diff(sign(diff(c(Inf, dens$y, Inf)))) == 2)    # index of max value  maxima <- which.max(dens$y)  maximum <- dens$x[maxima]    # combine indexes of local minima and the max  minmax <- sort(c(minima, maxima))    # select the two minima on either side of the max    nearestminima <- minmax[which(minmax == maxima) + c(-2, 2)]    # Numeric cutpoints  mincut <- dens$x[nearestminima[1]]  maxcut <- dens$x[nearestminima[2]]    # Zoomed in plot showing cut points (solid lines) and maximum (dotted line)    plot(dens, xlim = c((mincut-0.5), (maxcut+0.5)), main = "First recommended exclusions")  abline(v=mincut)  abline(v=maxcut)  abline(v=maximum, lty=2)    cuts <- c(mincut, maxcut, maximum)  return(cuts)  }findCutpointsLeftShoulder <- function(x){    dens <- density(x) # Kernel density estimate of supplied data    # Min/max method   minima  <- which(diff(sign(diff(c(Inf, dens$y, Inf)))) == 2)    # index of max value  maxima <- which.max(dens$y)  maximum <- dens$x[maxima]    # combine indexes of local minima and the max  minmax <- sort(c(minima, maxima))    # select the two minima on either side of the max    nearestminima <- minmax[which(minmax == maxima) + c(-2, 1)]    # Numeric cutpoints  mincut <- dens$x[nearestminima[1]]  maxcut <- dens$x[nearestminima[2]]    # Zoomed in plot showing cut points (solid lines) and maximum (dotted line)    plot(dens, xlim = c((mincut-0.5), (maxcut+0.5)), main = "First recommended exclusions")  abline(v=mincut)  abline(v=maxcut)  abline(v=maximum, lty=2)    cuts <- c(mincut, maxcut, maximum)  return(cuts)  }findCutpointsRightShoulder <- function(x){    dens <- density(x) # Kernel density estimate of supplied data    # Min/max method   minima  <- which(diff(sign(diff(c(Inf, dens$y, Inf)))) == 2)    # index of max value  maxima <- which.max(dens$y)  maximum <- dens$x[maxima]    # combine indexes of local minima and the max  minmax <- sort(c(minima, maxima))    # select the two minima on either side of the max    nearestminima <- minmax[which(minmax == maxima) + c(-1, 2)]    # Numeric cutpoints  mincut <- dens$x[nearestminima[1]]  maxcut <- dens$x[nearestminima[2]]    # Zoomed in plot showing cut points (solid lines) and maximum (dotted line)    plot(dens, xlim = c((mincut-0.5), (maxcut+0.5)), main = "First recommended exclusions")  abline(v=mincut)  abline(v=maxcut)  abline(v=maximum, lty=2)    cuts <- c(mincut, maxcut, maximum)  return(cuts)  }
