# Supplementary material for: Equilibrated Gas and Carbonate Standard-Derived Dual (Δ47 and Δ48) Clumped Isotope Values
Source: Geochem Geophys Geosyst. Author manuscript; Available in PMC 2023 Oct 12. (PMC10569407; doi:10.1029/2022gc010458)
Supplement: analyses [file NIHMS1842612-supplement-analyses.rtf]

# Steps to perform all cuts and export final data. "Stnd" is a placeholder for the name of the standard being called.library(openxlsx)# Find initial cutpointsdata_cuts <- findCutpoints(data$D48CDES_Final)# Perform first cutdata_firstcut <- data[data$D48CDES_Final >= data_cuts[1] & data$D48CDES_Final <= data_cuts[2] ]# Get summary stats following first cutmean(data_firstcut)sd(data_firstcut)length(data_firstcut)tiff(file = "Data_firstcut.tiff", width = 5, height = 5, units = "in", res = 800, compression = "lzw")plot(density(data_firstcut), main = expression(paste("Standard name (",Delta[48]*" \u2030), Config number")))abline(v=mean(data_firstcut)+sd(data_firstcut), col = "#FCA50AFF")abline(v=mean(data_firstcut)-sd(data_firstcut), col = "#FCA50AFF")abline(v=mean(data_firstcut)+(2*sd(data_firstcut)), col = "#DD513AFF")abline(v=mean(data_firstcut)-(2*sd(data_firstcut)), col = "#DD513AFF")abline(v=mean(data_firstcut)+(3*sd(data_firstcut)), col = "#6B186EFF")abline(v=mean(data_firstcut)-(3*sd(data_firstcut)), col = "#6B186EFF")abline(v=mean(data_firstcut), lty=2, col = "#170C3AFF")dev.off()# Perform 3sigma exclusion as the second cut. If the Shapiro-Wilk test (line 31) indicates non-normality, use 2sigma (2*sd) or 1sigma (1*sd) as needed.Stnd_secondcut <- Stnd_firstcut[Stnd_firstcut >= (mean(Stnd_firstcut)-(3*sd(Stnd_firstcut))) &                                 Stnd_firstcut <= (mean(Stnd_firstcut)+(3*sd(Stnd_firstcut)))]# Perform a Shapiro-Wilk test for normality following the 3 sigma exclusionshapiro.test(Stnd_secondcut)# Get summary stats following second cutmean(Stnd_secondcut)sd(Stnd_secondcut)length(Stnd_secondcut)# Quick plot to visualize the final dataplot(density(Stnd_secondcut), main = "Stnd Final Data")# Apply the calculated cuts to the full datasetStnd_final <- data[data$Standard == "Stnd" & data$D48CDES_Final >= range(Stnd_secondcut)[1] & data$D48CDES_Final <= range(Stnd_secondcut)[2],]# Create an Excel spreadsheet for the datawb <- createWorkbook("FinalData") # Create an empty workbook in the Global EnvironmentaddWorksheet(wb, "Stnd") # Add a blank sheet for the standard being cleanedwriteData(wb, sheet = "Stnd", Stnd_final) # Write the final, cleaned data to the blank sheet# Repeat above for each standard, then:# Final step is to save the complete workbook of datasaveWorkbook(wb, "Final Data.xlsx")# We suggest tracking basic summary stats, cuts used, final sigma, etc. for each standard following the example provided in All_standards_exclusions_config1.xlsx
