# Supplementary material for: Equilibrated Gas and Carbonate Standard-Derived Dual (Δ47 and Δ48) Clumped Isotope Values
Source: Geochem Geophys Geosyst. Author manuscript; Available in PMC 2023 Oct 12. (PMC10569407; doi:10.1029/2022gc010458)
Supplement: user manual [file NIHMS1842612-supplement-user_manual.pdf]

User manual

for Equilibrated gas and carbonate standard-derived dual (Δ47 and Δ48) clumped isotope values

Jamie K. Lucarelli, Hannah M. Carroll, Robert N. Ulrich1, Ben M. Elliott, Tyler B. Coplen, Robert A. Eagle, Aradhna Tripati

Implementation of replicate quality assurance methods

This section contains step-by-step instructions with examples for working with the custom R script used here for replicate data quality assurance. Note that replicate data presented here is unusually noisy for demonstration purposes, and more replicates are removed from this hypothetical dataset than what is typically observed in a real dataset. This hypothetical dataset is used to clearly demonstrate the steps involved and visualize changes in the replicate distribution.

Standardization

Standards with multiple aliquots should be grouped. For example, many labs will utilize ETH-1. Aliquots available are 1-4. If these are coded by aliquot number, such as ETH-1-1, ETH-1-2, etc., these should be grouped together and named ETH-1. As an example:

```
unique(data$Easotope_Name)

##  [1] "TV03"           "Veinstrom"      "Carmel Chalk"   "ETH-1-1"
##  [5] "ETH-2-1"        "ETH-3-1"        "ETH-4-1"        "Carrara Marble"
##  [9] "ETH-2-2"        "ETH-3-3"        "ETH-4-2"        "ETH-1-2"
## [13] "ETH-1-3"        "ETH-2-3"
```

We have multiple aliquots of ETH-1, ETH-2, ETH-3, and ETH-4. The rest of the standards are not given aliquot numbers. We just need to group the ETH standards. We use partial string matching for this purpose:

```
library(dplyr)

data <- data %>%
  mutate(Standard = case_when(
    !grepl("ETH", Easotope_Name) ~ Easotope_Name,
    grepl("ETH-1", Easotope_Name) ~ "ETH-1",
    grepl("ETH-2", Easotope_Name) ~ "ETH-2",
    grepl("ETH-3", Easotope_Name) ~ "ETH-3",
    grepl("ETH-4", Easotope_Name) ~ "ETH-4"
    )
  )
```

We now have standardized names as follows. Note that the original name is not overwritten - we add a new column and preserve the original designations.

```r
unique(data$Standard)

## [1] "TV03"          "Veinstrom"     "Carmel Chalk"  "ETH-1"
## [5] "ETH-2"         "ETH-3"         "ETH-4"         "Carrara Marble"
```

Finding initial cutpoints

Kernel density estimation is used to find initial cutpoints for the removal of extreme outliers from replicate pools. Bandwidth selection follows Silverman's rule of thumb (Silverman 1986).

When there is a singular peak, the findCutpoints function is employed as follows:

```r
ETH2_cuts <- findCutpoints(data$D48CDES_Final[data$Standard == "ETH-2"])
```

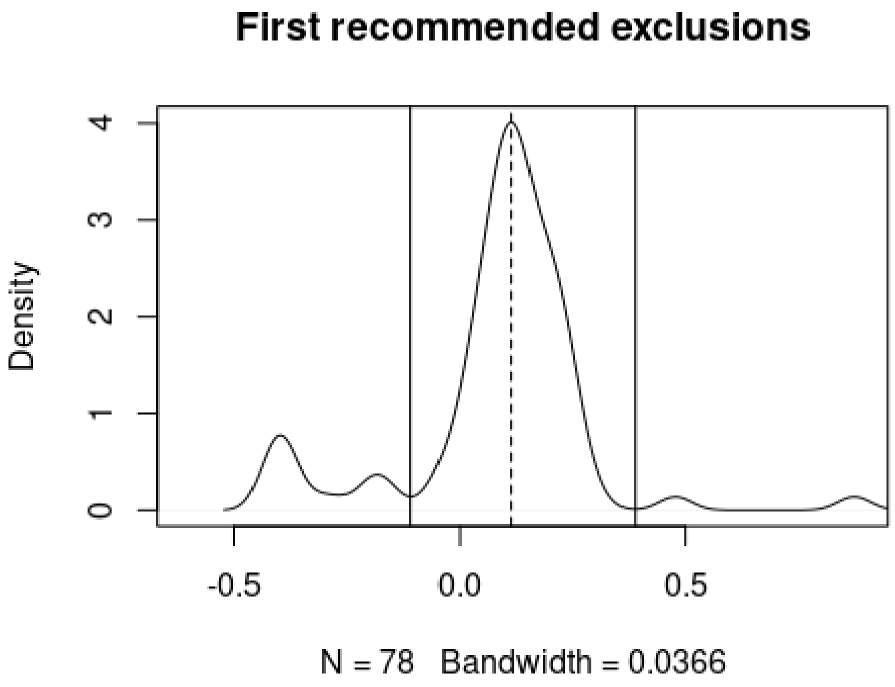

The recommended cuts (nearest minima on either side of the maxima) are marked in solid black vertical lines. In this case, recommended cuts are:

```r
ETH2_cuts[1]

## [1] -0.1093086

ETH2_cuts[2]

## [1] 0.3886321
```

Note that the functions do not round values, as we wish to avoid accumulating rounding error.

Those suggested cuts are stored in the object **ETH2_cuts**, then applied to the raw data as follows:

```
ETH2_firstcut <- data$D48CDES_Final[data$Standard == "ETH-2" &
                                    data$D48CDES_Final >= ETH2_cuts[1] &
                                    data$D48CDES_Final <= ETH2_cuts[2] ]
```

Following the exclusion of poorly constrained replicates, our data are as follows:

```
mean(ETH2_firstcut)

## [1] 0.1275455

sd(ETH2_firstcut)

## [1] 0.07563763

length(ETH2_firstcut)

## [1] 66
```

Final exclusions

A visual representation of density curve to three standard deviations:

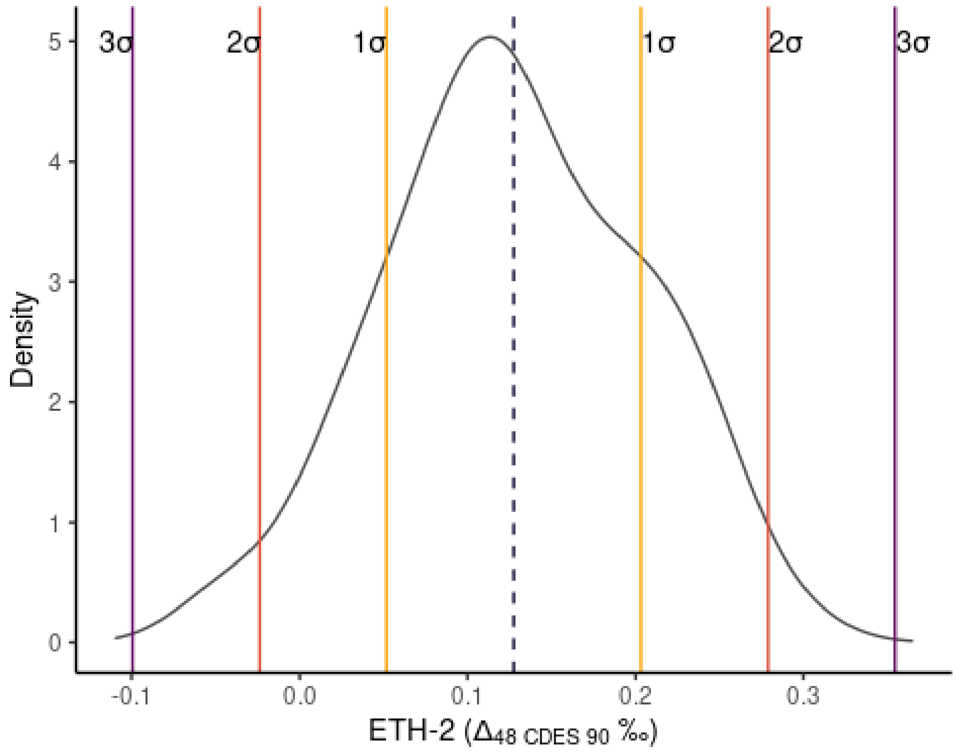

We then employ a 3σ exclusion (outermost pair of solid vertical lines in purple) to complete the quality control process:

```
ETH2_secondcut <- ETH2_firstcut[ETH2_firstcut >= (mean(ETH2_firstcut)-(3*sd(ETH2_firstcut))) &
                                ETH2_firstcut <= (mean(ETH2_firstcut)+(3*sd(ETH2_firstcut)))]
mean(ETH2_secondcut)

## [1] 0.1275455

sd(ETH2_secondcut)

## [1] 0.07563763

length(ETH2_secondcut)

## [1] 66
```

These final cuts are then applied to the full dataset:

```
ETH2_final <- data[data$Standard == "ETH-2" &
             data$D48CDES_Final >= range(ETH2_secondcut)[1] &
             data$D48CDES_Final <= range(ETH2_secondcut)[2],]
```

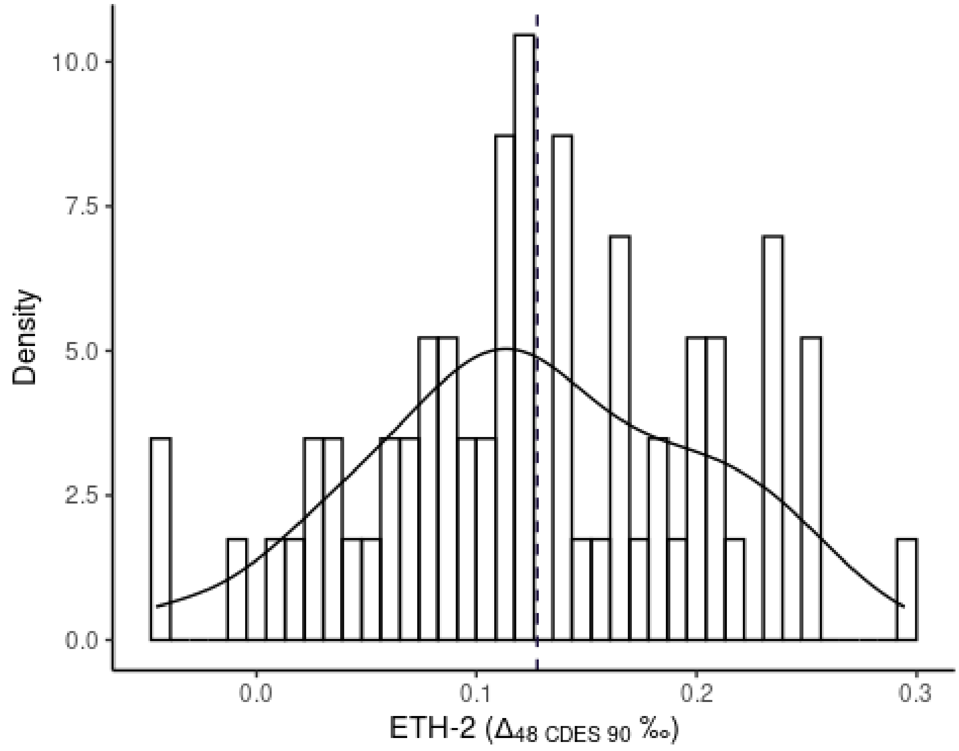

We test for normality of the final dataset as follows:

```
shapiro.test(ETH2_final$D48CDES_Final)

##
##  Shapiro-Wilk normality test
##
## data:  ETH2_final$D48CDES_Final
## W = 0.98847, p-value = 0.8011
```

The final data are consistent with a normal distribution.

Cleaning data when there are double peaks or shoulders

We provide an additional set of functions to address more complex situations. For double or shouldered peaks, the nearest minima will be inaccurate. Where a second peak or shoulder is approximately a third as high as the highest peak, these data should be included in the initial cut. This is most likely to occur when replicate pools are relatively small or when older instrumentation, such as Config. 3, is used to measure Δ48.

An example where the second peak is on the lefthand side of the maxima:

```
leftcut <- findCutpoints(data2$D47CDES_90[data2$Standard == "IAEA-C2"])
```

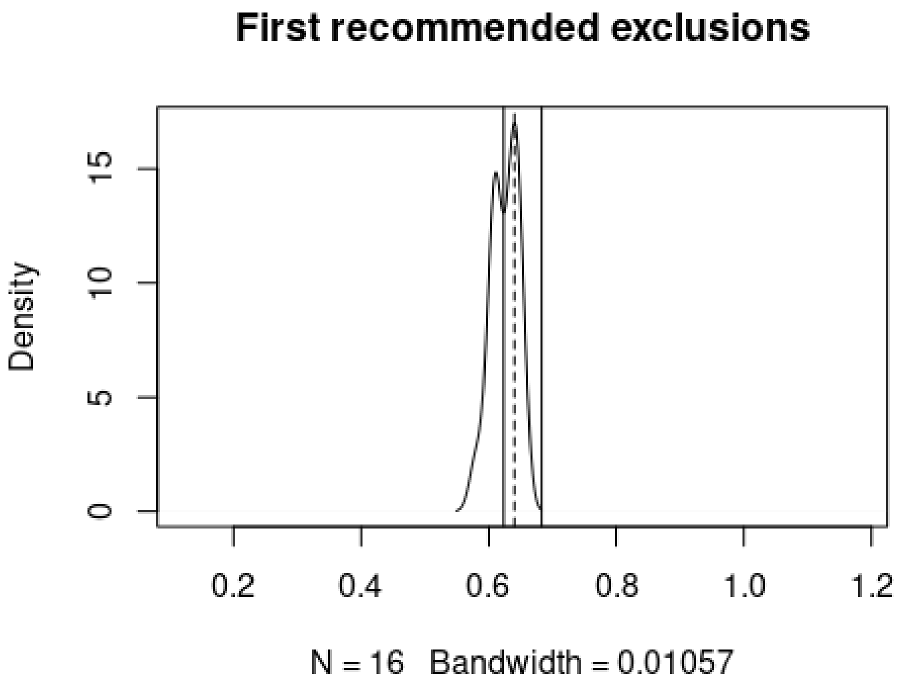

This will require the function findCutpointsLeftShoulder:

```
IAEA_C2_cuts <- findCutpointsLeftShoulder(data2$D47CDES_90[data2$Standard ==
"IAEA-C2"])
```

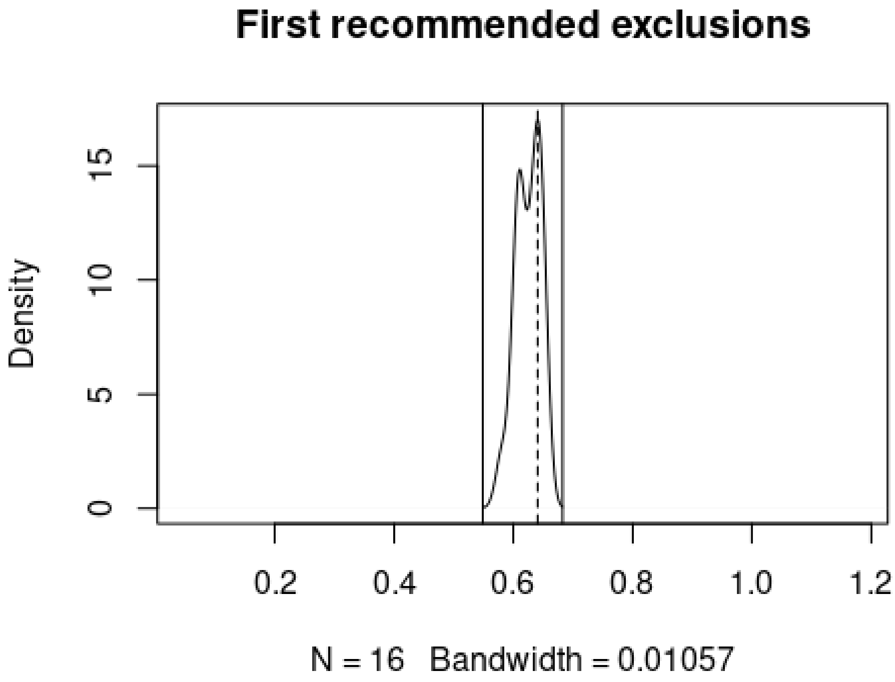

The findCutpointsRightShoulder function is provided for cases such as the example below:

```
c102GCAZ01_cuts <-
findCutpointsRightShoulder(data2$D47CDES_Final[data2$Standard == "102-GC-
AZ01"])
```

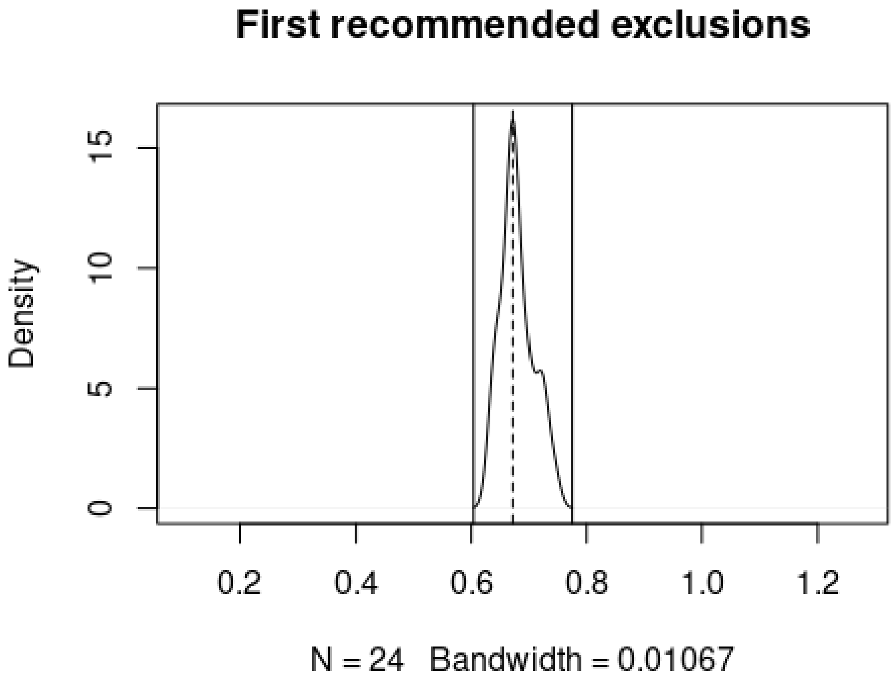

Peaks with shoulders on both sides:

```
ETH1_cuts <- findCutpointsDouble(data3$D48CDES_Final[data3$Standard == "ETH-
1"])
```

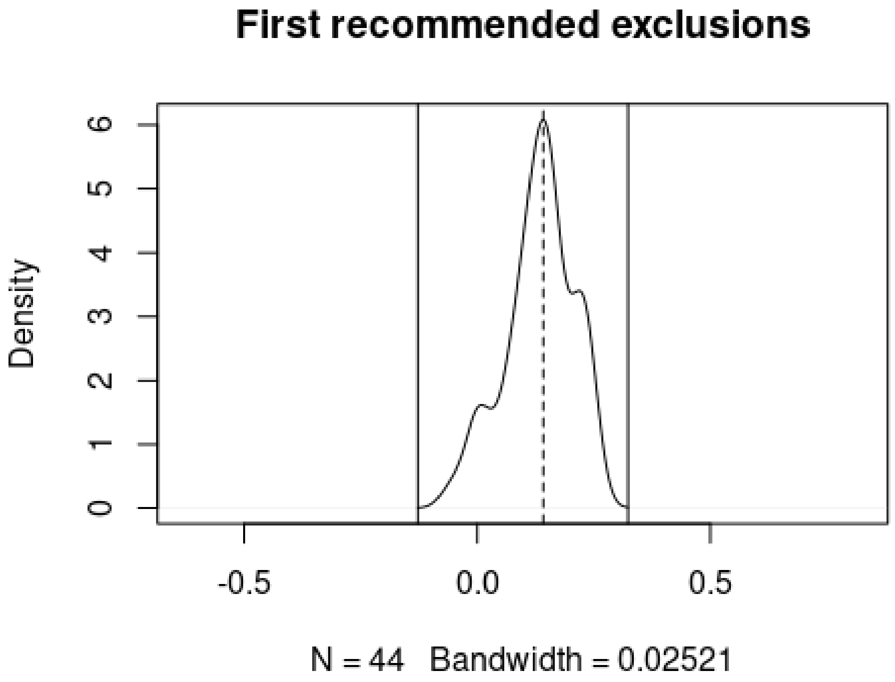

Following the initial exclusions, we then proceed with the final exclusions as described above.
