# Supplementary material for: Equilibrated Gas and Carbonate Standard-Derived Dual (Δ47 and Δ48) Clumped Isotope Values
Source: Geochem Geophys Geosyst. Author manuscript; Available in PMC 2023 Oct 12. (PMC10569407; doi:10.1029/2022gc010458)
Supplement: Text S1 to S3; Figures S1 to S6 [file NIHMS1842612-supplement-Text_S1_to_S3__Figures_S1_to_S6.pdf]

Geochemistry, Geophysics, Geosystems

Supporting Information for

Equilibrated gas and carbonate standard-derived dual (Δ_{47} and Δ_{48}) clumped isotope values

Jamie K. Lucarelli[1], Hannah M. Carroll[1], Robert N. Ulrich[1], Ben M. Elliott[1], Tyler B. Coplen[2], Robert A. Eagle[1], Aradhna Tripati[1]

[1]Department of Earth, Planetary, and Space Sciences, Department of Atmospheric and Oceanic Sciences, Institute of the Environment and Sustainability, Center for Diverse Leadership in Science, UCLA, Los Angeles, CA 90095 USA, [2]US Geological Survey, 12201 Sunrise Valley Drive, Reston, VA, 20192, USA

Contents of this file

Text S1 to S3
Figures S1 to S6

Additional Supporting Information (files uploaded separately)

Tables S1 to S27

Introduction

The Supporting Information included in this document are text detailing statistical methods used for data processing and calculation of acid fractionation factors. There are also figures that demonstrate mass spectrometer reproducibility, including data distributions and corrections applied to raw data.

The Supporting Information contained in a separate file, titled Supporting Information S2, includes results of statistical analyses and acid fractionation factor calculations, and all replicate data.

Text S1. Background of statistical methods used for sample replicate data

Term	Definition	Abbreviation
Estimated marginal mean	Mean calculated from a model fit to replicate data	EMM
Ordinary mean	Mean calculated directly from replicate data	OM
Standard deviation	σ (sigma)	SD
Standard error	Standard deviation divided by the square root of the number of replicates: $\frac{\sigma}{\sqrt{N}}$	SE

For Δ_{47} and Δ_{48} quality assurance, we adapted screening criteria used in other disciplines, implementing kernel density estimation as a statistical technique for use with clumped isotope data. Kernel density estimation has a broad range of research applications in the geosciences and related fields, including climate modeling (Pulkkinen, 2016), weather forecasting (Wahiduzzaman & Yeasmin, 2020), and ecology (Seaman & Powell, 1996), and in other fields such as economics (Correa-Quezada et al., 2020), and manufacturing (Lee et al., 2020), among others. For each sample replicate pool, we calculated a kernel density estimate using the generic S3 method 'density' included in base R's stats package (R Core Team, 2021). Kernel density estimation is used to examine the underlying probability density function (PDF) for a given variable. Each clumped isotope sample value is not a single definite point, due to the uncertainty inherent in replicate measurements, but is rather a finite probable range of values. This can be visualized as a peak where the most probable values for a given variable cluster together to produce the peak's maxima; this is the probability density function. This is similar to the way in which histograms illustrate data distributions based on counts (Figure 2A in main text).

In quality screening of clumped isotope data, a nonparametric approach, such as kernel density estimation, is preferred because we often have no *a priori* knowledge of the statistical properties of the raw clumped isotope replicate pool. In density estimation, a weighting function, known as a kernel, is applied to the replicate data; in the R implementation of kernel density estimation, the default is to use a normally distributed (Gaussian) kernel, *K*, applied to a variable, *u*. The normal distribution takes the form:

$$\mathrm{K}(u)=\frac{1}{\sqrt{2\pi}}e^{\frac{-1}{2}u^2}$$

The smoothing parameter, known as bandwidth, follows Silverman's rule of thumb (Silverman 1986). Bandwidth, *h*, is calculated thus:

$$h = 0.9 \cdot \min\left(\bar{\sigma}, \frac{IQR}{1.35}\right) N^{\frac{1}{5}}$$

where *IQR* is the interquartile range and *N* is the number of replicates. Silverman's rule of thumb is appropriate for the vast majority of replicate pools in our study, as they are primarily unimodal and reasonably close to normally distributed. Silverman's rule is relatively robust to violations of the assumption of normal distribution (Silverman 1986), which is especially important when the distributions of replicate pools may not be known *a priori*. When raw replicate pools present severe violations of the assumptions of Silverman's rule of thumb, other bandwidth choices may need to be substituted, such as that developed by Sheather & Jones (1991). The kernel is scaled so that *h* is the standard deviation of the kernel, or weighting function, itself (R Core Team, 2021). The kernel then becomes a curve that integrates to 1 with the statistical properties:

$$\sigma^2(K) = t^2 K(t)\, dt$$

For a full explanation of bandwidth selection in nonparametric probability density estimation, see Silverman (1986) and Sheather & Jones (1991); for a full explanation of kernel density estimation as implemented in R, see Deng & Wickham (2011).

After determining the PDF peak for each sample replicate pool, we found the nearest minima, or least probable values of the possible range, on either side of the maxima, or most probable value, and defined those minima as the initial cutpoints for exclusion of very large outliers (Figure 2B). In rare cases where the PDF revealed a double peak or a shoulder at least a third as high as the true maxima, we used the second nearest minima or left/right minima according to the shape of the density peak (examples given in the user manual). The choice to cut off peaks and shoulders <1/3 and include peaks and shoulders >1/3 as high as the maxima allowed for the inclusion of quality replicate values while excluding outlier replicates and produced final Δ_{47} sample mean values in good agreement with data processed using other methods (Table S5). Following the initial exclusion of very large outliers, replicates with values >3σ from the sample mean were excluded to yield final Δ_{47} and Δ_{48} replicate pools. A Shapiro-Wilk test was used to determine whether the resulting data were consistent with a normal distribution (Table S6, S7).

The PDF-based exclusion method has been included as a custom function in a publicly available R script and a brief user manual provided for those who wish to apply this method (https://doi.org/10.5281/zenodo.7311624).

Text S2. Inter-instrumental comparisons and pooling of replicate data

Five mass spectrometer configurations (Table 2) were used to measure clumped isotope replicate values in this study. To ensure it was appropriate to pool replicates produced using different mass spectrometer configurations, we performed statistical tests comparing individual sample mean values produced on each configuration and the overall cumulative comparability of each configuration.

To test for any differences between configurations that would preclude pooling replicate data, we first modeled each configuration's final sample mean Δ_{47} and Δ_{48} values by the additive effects of configuration and sample using a linear mixed effects model from package *nlme* version 3.1-152 (J Pinheiro et al., 2021), similar to what was performed for Δ_{47} data in Upadhyay et al. (2021). Linear mixed effects models allow for both fixed effects (the independent variables) and random effects (additional variables which may affect the dependent variables, but which are not being explicitly modeled). The SE of the final replicate pool was included as a random effect in the model. Including SE as a random effect in the model is done to capture the effect of differences in precision from measurement to measurement, and account for them in the calculation of model coefficients. SE is considered a random effect instead of a fixed effect (i.e., a predictor or independent variable) because it is not constant across replicates and allows the model to account for the non-uniform random error across replicates and replicate pools. Note that models did not include samples for which we have relatively few replicates, including ISTB-1, TB-1, TB-2, CIT Carrara, DH-2-10, DH-2-11, DH-2-12, DH-2-13, TV01, 47407 Coral, Spel-2-8-E, and 102-GC-AZ01.

Then, differences between configurations were assessed using estimated marginal means (EMMs) determined with package *emmeans* version 1.5.4 (Lenth, 2021). EMMs are based on a model fit to the data, rather than the raw data. This has the effect of controlling for unequal numbers of observations per group, such that the final value for a given sample produced by an instrument which has an N = 50 is not downweighted relative to an instrument which has an N = 150. This is useful when comparing the values produced by one instrument to another, as unequal numbers of replicates violate the assumptions of most parametric statistical tests and may lead to erroneous conclusions. As the estimated marginal means differ slightly from the more commonly reported ordinary means (average of all replicate values), we use them strictly for inter-instrumental comparisons, and report ordinary means throughout the rest of the manuscript. Data were pooled for further analyses only if there was no evidence of a statistically significant difference between configurations.

Text S3. Regression-form acid digestion fractionation factors, $\Delta^*_{63\text{-}47}$ and $\Delta^*_{64\text{-}48}$

Model calculations from Guo et al. (2009) predicted that acid digestion fractionation factors (AFFs), $\Delta^*_{63\text{-}47}$ and $\Delta^*_{64\text{-}48}$, for when an O atom is cleaved from CO_3^{2-} when calcite is digested in phosphoric acid, should depend on the Δ_{63} and Δ_{64} values of the reactant carbonate, respectively. To calculate this dependence, we first calculated nonlinear regressions of the theoretical calcite equilibrium Δ_{63} and Δ_{64} values versus temperature (Hill et al., 2014; Tripati et al., 2015). These regressions were used to calculate Δ_{63} and Δ_{64} values for the precipitation temperature of 33.7 °C ($\Delta_{63} \approx 0.3707$ ‰; $\Delta_{64} \approx 0.1092$ ‰) and 600 °C ($\Delta_{63} \approx 0.0179$ ‰; $\Delta_{64} \approx 0.0022$ ‰). Then, the difference was calculated between the theoretical Δ_{63} and Δ_{64} values for 33.7 °C and the measured Δ_{47} and Δ_{48} values, respectively, for pooled replicate values from Devils Hole calcite samples DH-2-10, DH-2-11, DH-2-12, and DH-2-13 (Table 5), which have a known precipitation temperature of 33.7 ± 0.8 °C (Winograd et al., 1992). Additionally, the

difference was calculated between the theoretical Δ_{63} and Δ_{64} values for 600 °C and the measured Δ_{47} and Δ_{48} values, respectively, for pooled replicate values from ETH-1 and ETH-2 (Table 4), which have an equilibration temperature of 600 °C (Bernasconi et al., 2018). These calculated differences yielded the AFFs for calcite at 33.7 °C ($\Delta^*_{63\text{-}47}$ = 0.1949 ‰; $\Delta^*_{64\text{-}48}$ = 0.1308 ‰) and 600 °C ($\Delta^*_{63\text{-}47}$ = 0.1881 ‰; $\Delta^*_{64\text{-}48}$ = 0.1300 ‰) using equations S1 and S2.

$$\Delta^*_{63\text{-}47} = \Delta_{47\ \text{I-CDES}} - \Delta_{63} \qquad \text{S1}$$

$$\Delta^*_{63\text{-}47} = \Delta_{48\ \text{CDES 90}} - \Delta_{64} \qquad \text{S2}$$

Devils Hole calcite was used to calculate the AFFs because it is assumed to have precipitated near isotopic equilibrium due to an extremely slow precipitation rate (0.1-0.8 μm year-1), low calcite saturation index (0.16-0.21), and a stable temperature of 33.7 (±0.8) °C throughout the Holocene (Coplen, 2007; Winograd et al., 1992, 2006). ETH-1 and ETH-2 were used because their Δ_{47} and Δ_{48} values were statistically indistinguishable, and samples equilibrated at high temperatures are less likely to have measurable kinetic effects due to decreased isotopic equilibration time.

Linear regressions were then calculated for $\Delta^*_{63\text{-}47}$ versus Δ_{63}, and $\Delta^*_{64\text{-}48}$ versus Δ_{64} for 33.7 °C and 600 °C (Figure S1a-b). The slope and intercept from these regressions were used to calculate $\Delta^*_{63\text{-}47}$ and $\Delta^*_{64\text{-}48}$ for 0-1000 °C (Table S9), using equations S3 and S4.

$$\Delta^*_{63\text{-}47} = 0.0193\ \Delta_{63} + 0.1878 \qquad \text{S3}$$

$$\Delta^*_{64\text{-}48} = 0.0077\ \Delta_{64} + 0.1300 \qquad \text{S4}$$

For samples with unknown precipitation temperature, $\Delta^*_{63\text{-}47}$ and $\Delta^*_{64\text{-}48}$ can be calculated using equations S5 and S6 (Figure S1c-d).

$$\Delta^*_{63\text{-}47} = 0.0190\ \Delta_{47\ \text{I-CDES}} + 0.1842 \qquad \text{S5}$$

$$\Delta^*_{64\text{-}48} = 0.0077\ \Delta_{48\ \text{CDES 90}} + 0.1290 \qquad \text{S6}$$

The relationship between precipitation temperature and $\Delta^*_{63\text{-}47}$ from 0-600 °C (Figure S1e) is represented by equation S7 (r^2 =0.99). The relationship between precipitation temperature and $\Delta^*_{64\text{-}48}$ (Figure S1f) from 0-600 °C is represented by equation S8 (r^2 = 0.99). Equations S7 and S8 use degrees in Celsius.

S7

$$\Delta^*_{63\text{-}47} = (0.1968 \pm 1.805 \times 10^{-5}) - (6.111 \times 10^{-5} \pm 5.894 \times 10^{-7})\ T + (1.922 \times 10^{-7} \pm 4.733 \times 10^{-9})\ T^2 - (2.965 \times 10^{-10} \pm 1.304 \times 10^{-11})\ T^3 + (1.762 \times 10^{-13} \pm 1.126 \times 10^{-14})\ T^4$$

S8

$\Delta^*_{64\text{-}48}$ = (0.1312 ± 6.955 × 10^{-6}) - (1.113 × 10^{-5} ± 2.271 × 10^{-7}) T + (4.398 × 10^{-8} ± 1.824 x 10^{-9}) T^2 - (7.799 × 10^{-11} ± 5.025 × 10^{-12}) T^3 + (5.041 × 10^{-14} ± 4.340 × 10^{-15}) T^4

The relationship between $\Delta^*_{63\text{-}47}$ and $\Delta^*_{64\text{-}48}$ is represented by equation S9.

S9

$\Delta^*_{64\text{-}48}$ = (0.3964 ± 0.0033) + (-2.898 ± 0.0340) $\Delta^*_{63\text{-}47}$ + (7.88 ± 0.0887) $\Delta^{*2}_{63\text{-}47}$

Equations S10 and S11 may be used to calculate Δ_{63} and Δ_{64} from Δ_{47} and Δ_{48} values.

S10

Δ_{63} = (-0.1845 ± 0.0007) + (0.9839 ± 0.0078) $\Delta_{47\ \text{I-CDES}}$ + (-0.0121 ± 0.0299) $\Delta_{47\ \text{I-CDES}}^2$ + (0.0207 ± 0.0483) $\Delta_{47\ \text{I-CDES}}^3$ + (-0.0125 ± 0.0281) $\Delta_{47\ \text{I-CDES}}^4$

S11

Δ_{64} = (-0.1377 ± 0.0048) + (1.166 ± 0.0981) $\Delta_{48\ \text{CDES 90}}$ + (-1.267 ± 0.7306) $\Delta_{48\ \text{CDES 90}}^2$ + (4.007 ± 2.363) $\Delta_{48\ \text{CDES 90}}^3$ + (-4.645 ± 2.807) $\Delta_{48\ \text{CDES 90}}^4$

The $\Delta^*_{63\text{-}47}$ versus Δ_{63} slope of 0.0193 determined here (Figure S1a) differs by -0.0112 from the model predicted slope from Guo et al. (2009) of 0.0305. The model calculated the dependence based on carbonates with $\delta^{13}C$ = 0 ‰ and $\delta^{18}O$ = 0 ‰, however, this may not be the source of the offset because the slope is only predicted to change by ~0.002 ‰ and ~-0.0005 ‰ for a 50 ‰ increase in $\delta^{13}C$ and $\delta^{18}O$, respectively (Guo et al., 2009). The slope offset may in-part arise from approximations made in the model calculations for isotopologues containing ^{17}O, and uncertainty in the slope determined in this study from the use of only two endpoint temperatures.

Fiebig et al. (2019) used a similar method to determine AFFs at 600 °C. Our 600 °C $\Delta^*_{63\text{-}47}$ and $\Delta^*_{64\text{-}48}$ values differed by 0.008 ‰ and 0.006 ‰, respectively, from their values of 0.196 ‰ and 0.136 ‰. Because the calculation of AFFs relies on the measured Δ_{47} and Δ_{48} values of ETH-1 and ETH-2, the difference in AFFs is equivalent to the difference in the pooled replicate average of ETH-1 and ETH-2 Δ_{47} and Δ_{48} values from this study ($\Delta_{47\ \text{I-CDES}}$ = 0.206 ± 0.001 ‰; $\Delta_{48\ \text{CDES 90}}$ = 0.132 ± 0.002 ‰) versus Fiebig et al. (2019) ($\Delta_{47\ \text{I-CDES}}$ = 0.214 ± 0.005 ‰; $\Delta_{48\ \text{CDES 90}}$ = 0.138 ± 0.015 ‰).

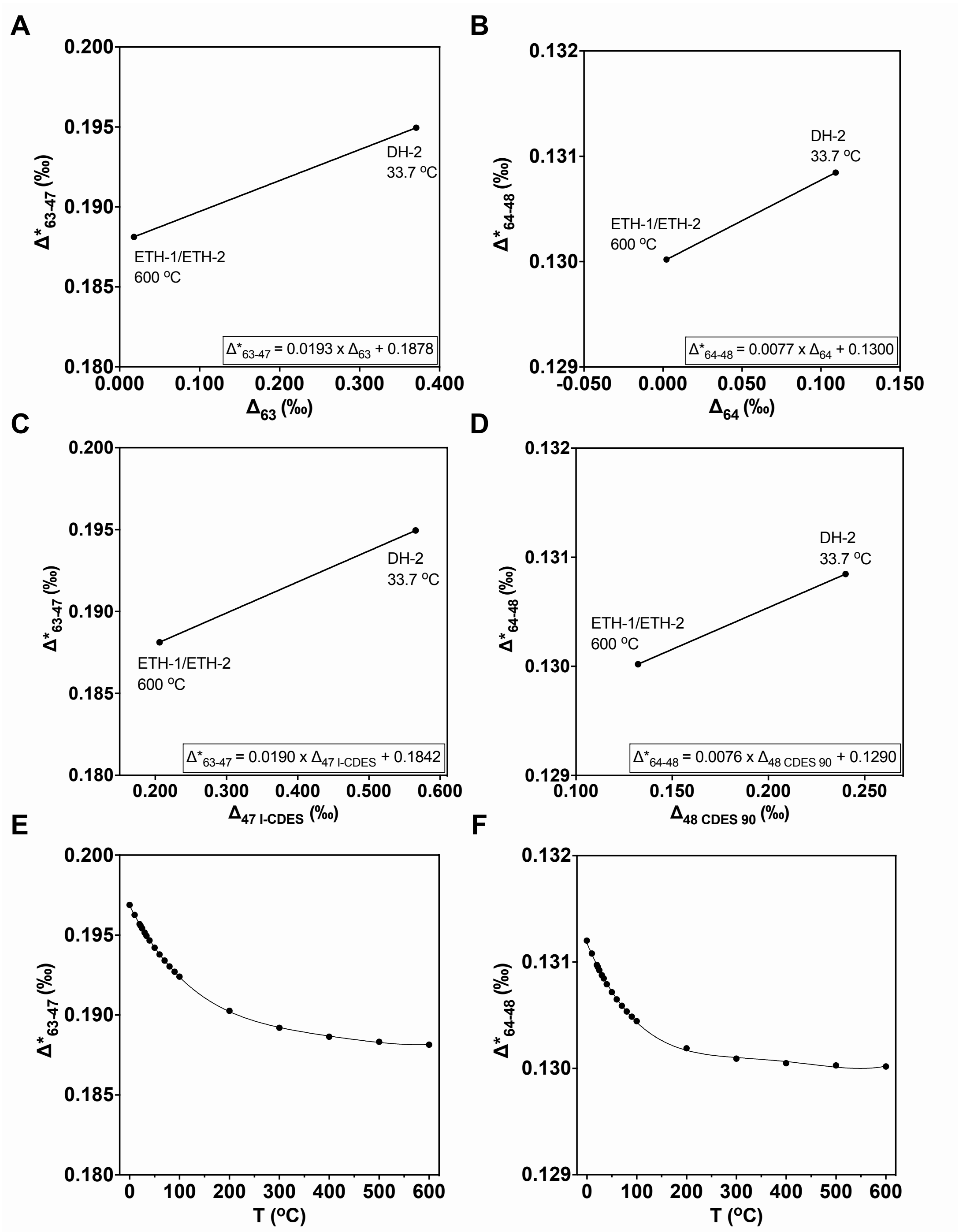

Figure S1. Constraints on acid digestion fractionation factors, $\Delta^*_{63\text{-}47}$ and $\Delta^*_{64\text{-}48}$. Regressions for A) $\Delta^*_{63\text{-}47}$ versus theoretical calcite Δ_{63} (Hill et al., 2014; Tripati et al., 2015); B) $\Delta^*_{64\text{-}48}$ versus theoretical calcite Δ_{64} (Hill et al., 2014; Tripati et al., 2015); C) $\Delta^*_{63\text{-}47}$ versus measured Δ_{47}; D) $\Delta^*_{64\text{-}48}$ versus measured Δ_{48}; E) $\Delta^*_{63\text{-}47}$ versus calculated precipitation temperature (r^2 = 0.99); F) $\Delta^*_{64\text{-}48}$ versus calculated precipitation temperature (r^2 = 0.99).

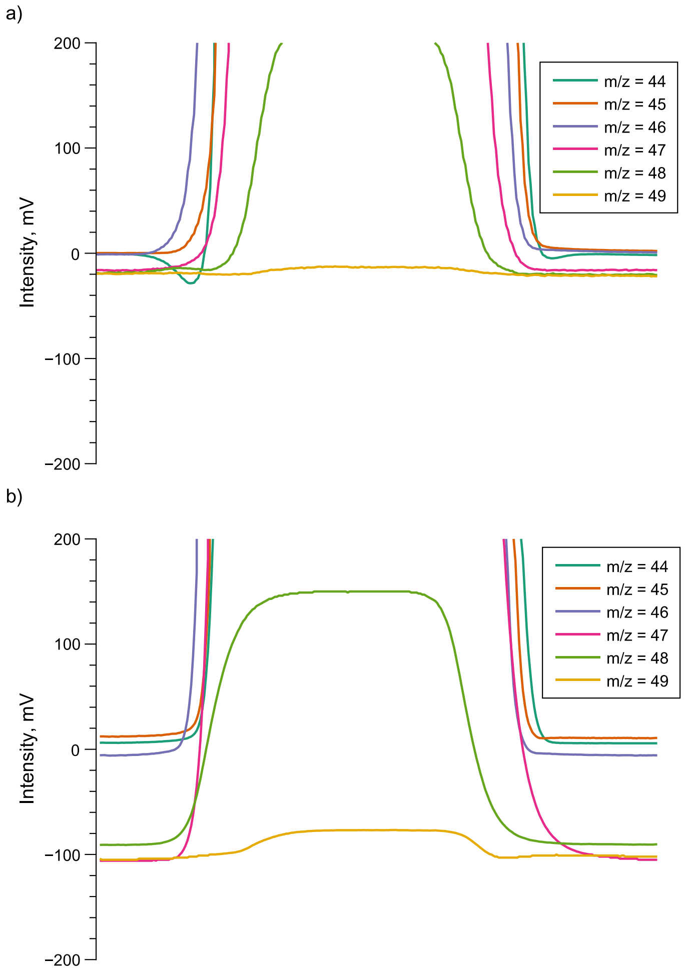

Figure S2. Simultaneous peakshape scans of the CO_2 mass peaks on A) the MAT 253, and B) Nu Perspective-1. The Nu Perspective-1 scans have been converted to mV so that the scans are immediately comparable. The older generation MAT 253 shows negatively sloped baselines, most readily seen on the *m/z* = 44 scan, that is not present in Nu Perspective-1. This is from negative interference from electrons which causes a decrease in the overall peak height. The flatter baselines of the Nu Perspectives allow for larger and more stable intensities of the higher masses (*m/z* = 47-49) to be recorded.

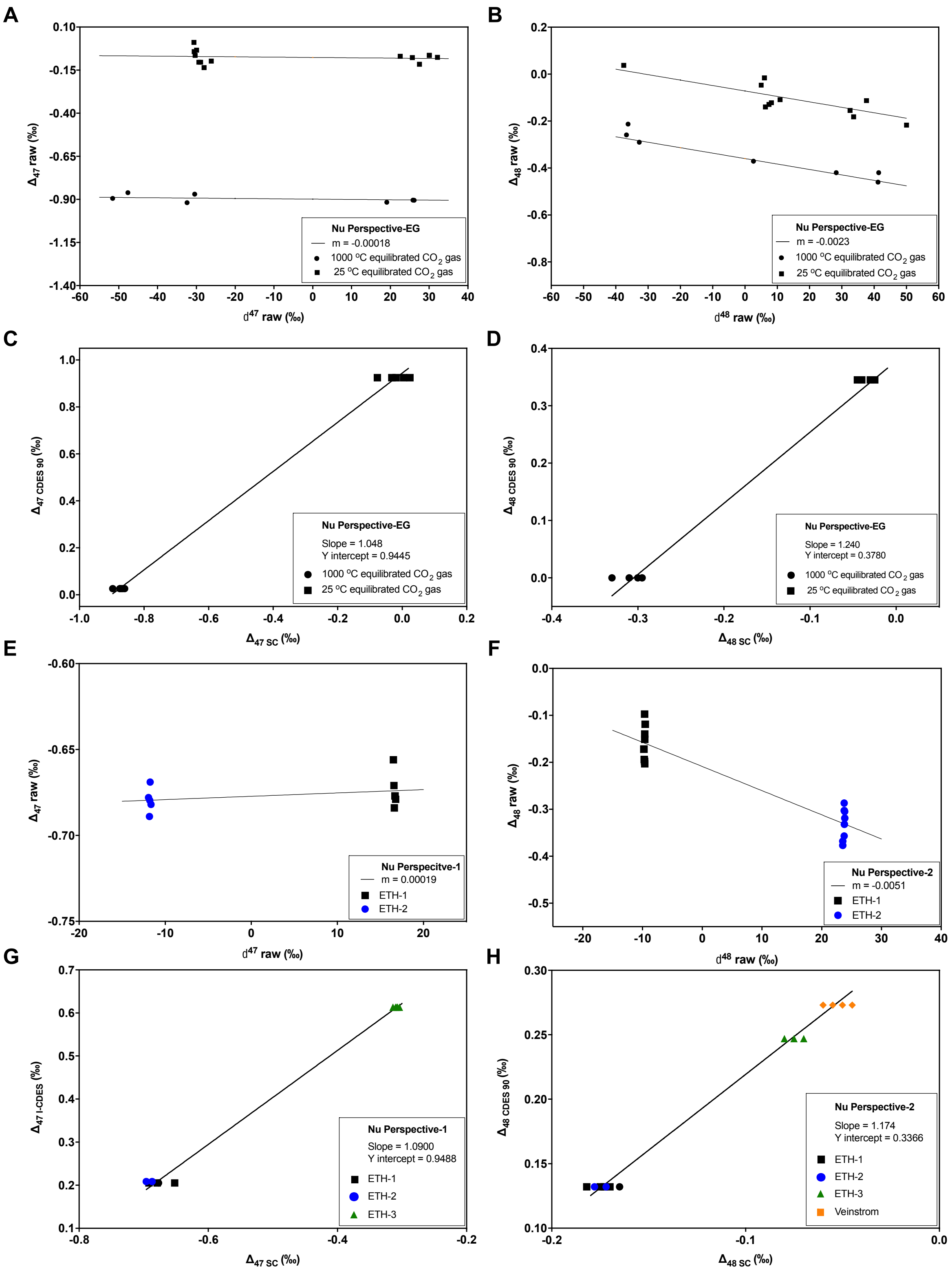

Figure S3. Representative examples of slope corrections and transfer functions performed for data normalization. A) and B) are the slope corrections using equilibrated gas standards on Nu Perspective-EG for Δ_{47} and Δ_{48}, respectively. C) and D) are transfer

functions using equilibrated gas standards on Nu Perspective-EG for Δ_{47} and Δ_{48}, respectively. E) and F) are the slope corrections using carbonate standards ETH-1 and ETH-2 on Nu Perspective-1 for Δ_{47} and Δ_{48}, respectively. G) and H) are transfer functions using carbonate standards ETH-1, ETH-2, and ETH-3, and Veinstrom (for Δ_{48} only) on Nu Perspective-1 for Δ_{47} and Δ_{48}, respectively. The slopes are determined on a $\pm$10-replicate moving interval to account for instrument drift and applied to standards and samples. Data normalization is performed similarly on all instruments.

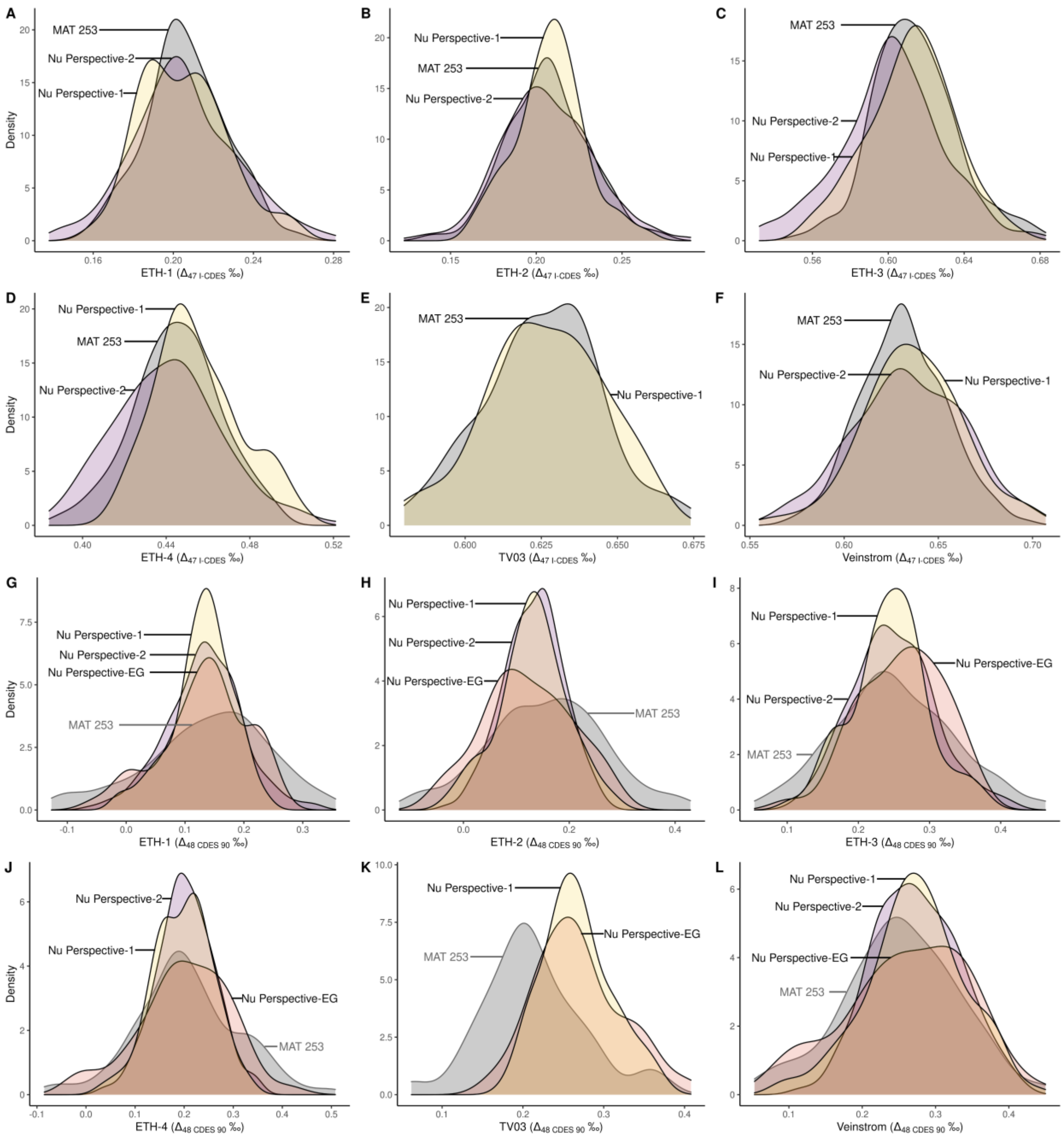

Figure S4. Final sample replicate density distributions for ETH-1, ETH-2, ETH-3, ETH-4, TV03, and Veinstrom, measured on multiple instruments and configurations for Δ_{47} values (A-F) and Δ_{48} values (G-L). We found no statistically significant differences in final mean values. A) Δ_{47} values for ETH-1; B) Δ_{47} values for ETH-2; C) Δ_{47} values for ETH-3; D) Δ_{47} values for ETH-4; E) Δ_{47} values for TV03; F) Δ_{47} values for Veinstrom; G) Δ_{48} values for ETH-1; H) Δ_{48} values for ETH-2; I) Δ_{48} values for ETH-3; J) Δ_{48} values for ETH-4; K) Δ_{48} values for TV03; L) Δ_{48} values for Veinstrom.

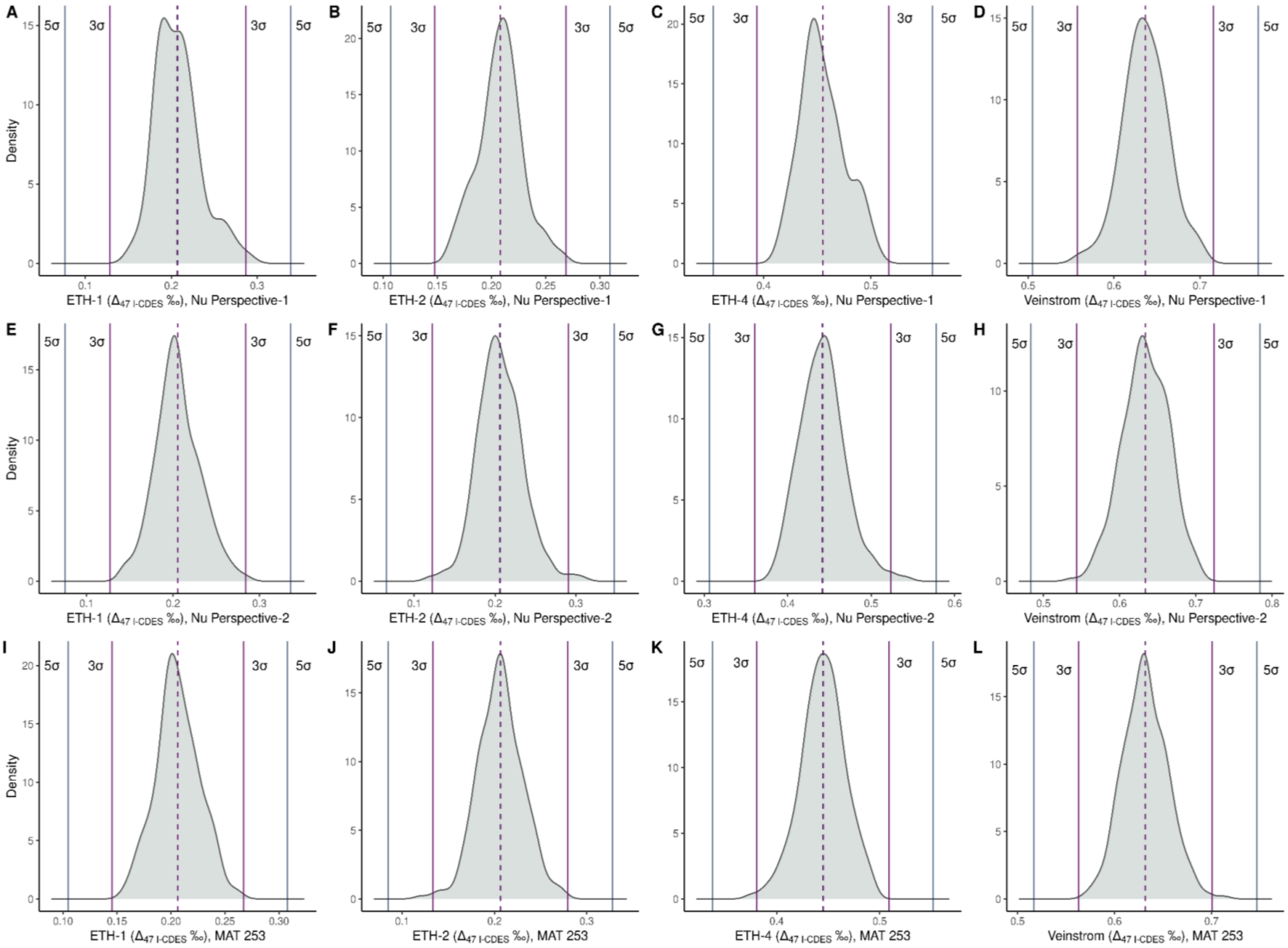

Figure S5. Density curves for Δ_{47} replicate pools for ETH-1, ETH-2, ETH-4 and Veinstrom on Nu-Perspective-1 (A-D), Nu-Perspective-2 (E-H), and MAT 253 (I-L). In all plots, dashed vertical lines indicate the replicate pool mean using a 3σ cutoff (purple) and 5σ cutoff (blue). The small differences in mean values using a 3σ versus 5σ cutoff are the result of large outliers being removed during the initial exclusions (see Figure 2). Note that these lines are too close together to be visually distinguished and so the mean values are reported in the following text: A) 3σ cutoff, final mean = 0.2066 ‰, SD = 0.025, N = 85; 5σ cutoff, final mean = 0.2076 ‰, SD = 0.026, N = 86; B) 3σ or 5σ cutoff, final mean = 0.2081 ‰, SD = 0.020, N = 69; C) 3σ or 5σ cutoff, final mean = 0.4552 ‰, SD = 0.020, N = 64; D) 3σ or 5σ cutoff, final mean = 0.6365 ‰, SD = 0.026, N = 102; E) 3σ cutoff, final mean = 0.2053 ‰, SD = 0.026, N = 402; 5σ cutoff, final mean = 0.2055 ‰, SD = 0.026, N = 403; F) 3σ cutoff, final mean = 0.2060 ‰, SD = 0.026, N = 386; 5σ cutoff, final mean = 0.2065 ‰, SD = 0.028, N =390. G) 3σ cutoff, final mean = 0.4411 ‰, SD = 0.026, N = 191; 5σ cutoff, final mean = 0.4420 ‰, SD = 0.027, N = 193; H) 3σ cutoff, final mean = 0.6341 ‰, SD = 0.030, N = 322; 5σ cutoff, final mean = 0.6338 ‰,

SD = 0.030, N = 323; I) 3σ or 5σ cutoff, final mean = 0.2063 ‰, SD = 0.020, N = 284; J) 3σ cutoff, final mean = 0.2066 ‰, SD = 0.024, N = 271; 5σ cutoff, final mean = 0.2063 ‰, SD = 0.024, N = 272. K) 3σ cutoff, final mean = 0.4451 ‰, SD = 0.021, N = 208; 5σ cutoff, final mean = 0.4448 ‰, SD = 0.021, N = 209; L) 3σ cutoff, final mean = 0.6315 ‰, SD = 0.022, N = 304; 5σ cutoff, final mean = 0.6318 ‰, SD = 0.023, N = 305.

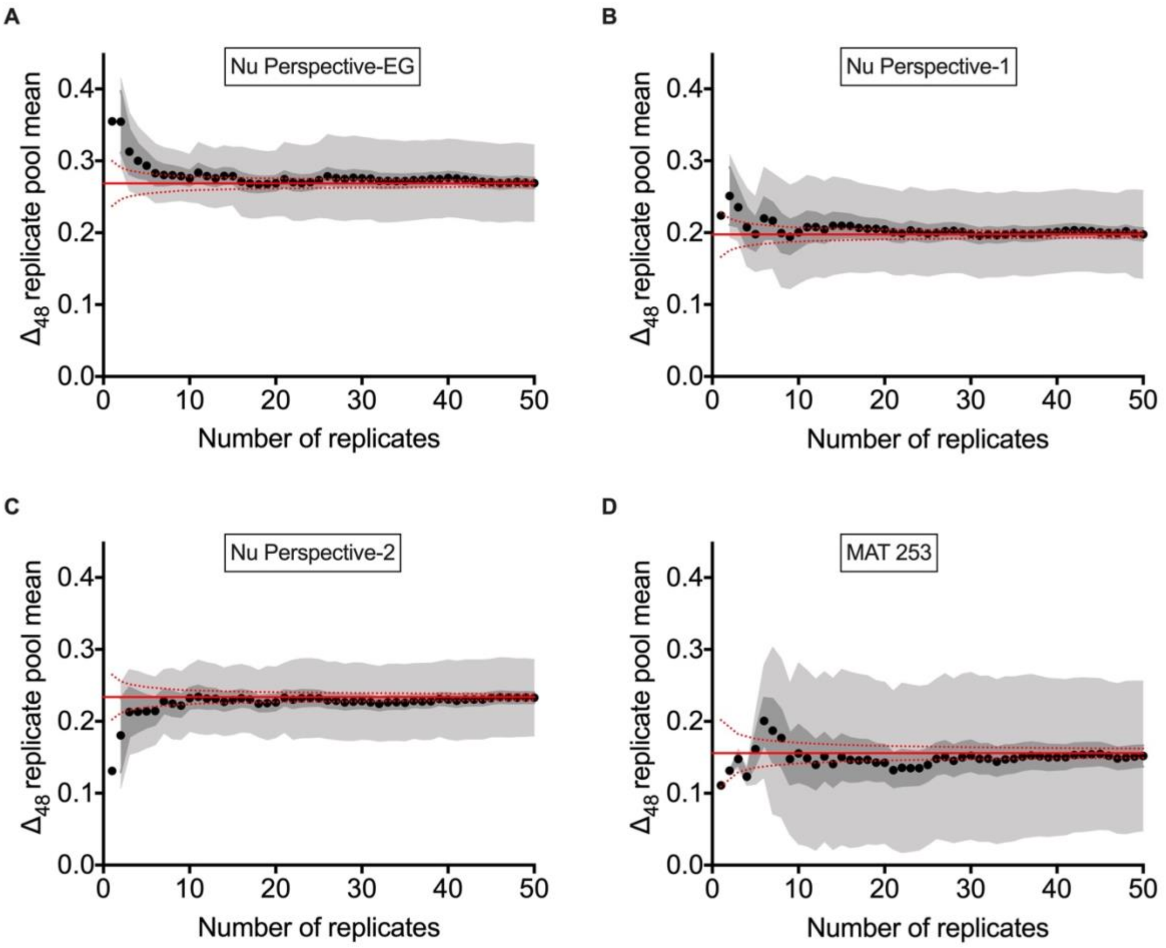

Figure S6. Representative examples of long term Δ_{48} replicate reproducibility for the instrument configurations used in this study. Shown in each panel are replicates (black circles), the long-term replicate pool average (solid red line), the upper and lower bounds of the shot noise limit (dotted red lines), replicate pool SE (dark gray shading), and replicate pool SD (light gray shading). None of the samples shown were used for standardization. A) TV03 analyzed on Nu Perspective-EG; B) ETH-4 analyzed on Nu-Perspective-1; C) Merck analyzed on Nu Perspective-2; CM Tile analyzed on MAT 253.
